# Recent Advances in Regulation Strategy and Catalytic Mechanism of Bi-Based Catalysts for CO_2_ Reduction Reaction

**DOI:** 10.1007/s40820-025-01860-8

**Published:** 2025-08-08

**Authors:** Jianglong Liu, Yunpeng Liu, Shunzheng Zhao, Baotong Chen, Guang Mo, Zhongjun Chen, Yuechang Wei, Zhonghua Wu

**Affiliations:** 1 College of Science, China University of Petroleum (Beijing), Beijing, 102249 People’s Republic of China; 2 Multi-Discipline Research Center, Institute of High Energy Physics, Chinese Academy of Sciences, Beijing, 100049 People’s Republic of China; 3 University of Chinese Academy of Sciences, Chinese Academy of Sciences, Beijing, 100049 People’s Republic of China; 4 Key Laboratory of Advanced Energy Materials Chemistry (Ministry of Education), Nankai University, Tianjin, 300071 People’s Republic of China; 5 University of Science and Technology Beijing, Beijing, 100083 People’s Republic of China

**Keywords:** Bismuth-based catalysts, CO_2_ reduction reaction, Regulation strategy, Catalytic mechanism, Review

## Abstract

Using photoelectrocatalytic CO_2_ reduction reaction (CO_2_RR) to produce valuable fuels is a fascinating way to alleviate environmental issues and energy crises. Bismuth-based (Bi-based) catalysts have attracted widespread attention for CO_2_RR due to their high catalytic activity, selectivity, excellent stability, and low cost. However, they still need to be further improved to meet the needs of industrial applications. This review article comprehensively summarizes the recent advances in regulation strategies of Bi-based catalysts and can be divided into six categories: (1) defect engineering, (2) atomic doping engineering, (3) organic framework engineering, (4) inorganic heterojunction engineering, (5) crystal face engineering, and (6) alloying and polarization engineering. Meanwhile, the corresponding catalytic mechanisms of each regulation strategy will also be discussed in detail, aiming to enable researchers to understand the structure–property relationship of the improved Bi-based catalysts fundamentally. Finally, the challenges and future opportunities of the Bi-based catalysts in the photoelectrocatalytic CO_2_RR application field will also be featured from the perspectives of the (1) combination or synergy of multiple regulatory strategies, (2) revealing formation mechanism and realizing controllable synthesis, and (3) in situ multiscale investigation of activation pathways and uncovering the catalytic mechanisms. On the one hand, through the comparative analysis and mechanism explanation of the six major regulatory strategies, a multidimensional knowledge framework of the structure–activity relationship of Bi-based catalysts can be constructed for researchers, which not only deepens the atomic-level understanding of catalytic active sites, charge transport paths, and the adsorption behavior of intermediate products, but also provides theoretical guiding principles for the controllable design of new catalysts; on the other hand, the promising collaborative regulation strategies, controllable synthetic paths, and the in situ multiscale characterization techniques presented in this work provides a paradigm reference for shortening the research and development cycle of high-performance catalysts, conducive to facilitating the transition of photoelectrocatalytic CO_2_RR technology from the laboratory routes to industrial application.

## Highlights

- Six major types of structural regulation strategies of various Bi-based catalysts used in photoelectrocatalytic CO_2_ reduction reaction (CO_2_RR) in recent years are comprehensively summarized.
- The corresponding catalytic mechanisms of each regulation strategy are discussed in detail, aiming to enable researchers to understand the structure–property relationship of the improved Bi-based catalysts fundamentally.
- The challenges and future opportunities of the Bi-based catalysts in the photoelectrocatalytic CO_2_RR application field are featured from the perspectives of the combination of multiple regulatory strategies, revealing formation mechanism and realizing controllable synthesis, and in situ multiscale investigation of activation pathways and uncovering the catalytic mechanisms.

## Graphical abstract

**Figure:**
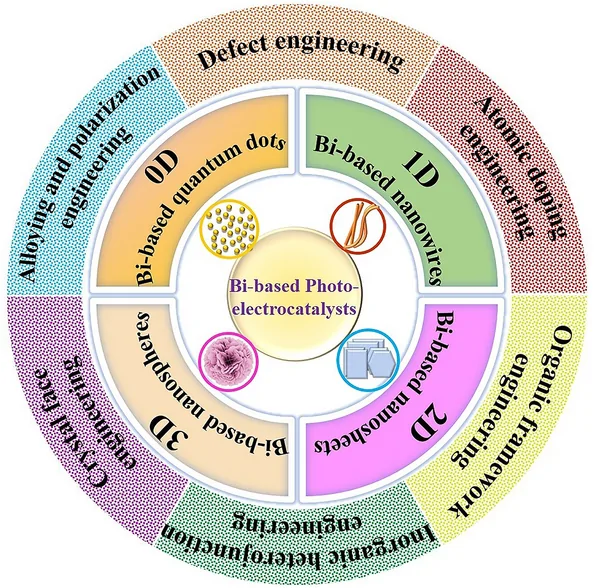

## Introduction

Nowadays, the concentration of carbon dioxide (CO_2_) in the atmosphere is rising significantly due to the combustion of fossil fuels during the rapid process of industrialization. This trend contributes to humans' major challenges in environmental pollution [1] and energy crises [2, 3]. Reducing CO_2_ emissions is a critical strategy for mitigating global warming. Still, from another perspective, CO_2_ also serves as a valuable carbon source; it can be transformed into useful chemicals, fuels, or other materials [4–6] through conversion methods like photocatalysis [7] and electrocatalysis [8, 9]. By employing these innovative techniques to convert excess CO_2_ into high-value-added fuels or chemicals, such as CO [10, 11], CH_4_ [12], HCOOH [13–15], CH_3_OH [16], and C_2_H_4_ [17], we can alleviate the energy crisis, reduce the reliance on traditional fossil fuels, and then promote the sustainable development for human society [18]. Therefore, it is urgent and essential to design and develop highly efficient catalysts to enhance the reduction and conversion of CO_2_.

Currently, bismuth-based (Bi-based) catalysts [19] from zero-dimensional (0D) to three-dimensional (3D) have garnered significant attention and exhibit excellent photoelectrocatalytic performance in the CO_2_ reduction reaction (CO_2_RR) due to their intrinsic structural properties, including a band gap suitable for visible light response, a unique layered structure, and a controllable electronic structure as well as interfacial microenvironment. Typically, Bi is utilized in two forms for CO_2_RR: Bi^0^ and Bi^3+^. The former, such as some pure Bi nanoparticles [20] and Bi nanosheets [21],can be directly used as semiconductor photoelectrocatalysts, or form alloy materials with other elements, such as Bi–Sn alloys [22] and Bi–Cu alloys [23], and can also effectively optimize the photoelectrocatalytic behavior of the target material; the latter, Bi^3+^, is the most common form in Bi-based catalysts, such as sulfides (Bi_2_S_3_ [24]), oxides (Bi_2_O_3_ [25]), polyoxides (Bi_2_O_2_CO_3_ [26], Bi_2_WO_6_ [27], BiFeO_3_ [28]), halogen oxides (BiOI [29], BiOBr [1], BiOF [30]), and perovskite materials (Cs_3_BiBr_9_ [31]), and possesses stronger redox capabilities, which enables it to capture and utilize electrons more effectively in the photoelectrocatalytic processes, thus promoting the CO_2_RR. The main advantages of Bi-based photoelectrocatalysts in the field of CO_2_RR can be attributed to four aspects. (i) Tunable electronic properties due to the unique 6*s*^2^6*p*^3^ electronic configuration, which enhances charge transfer [32, 33]. The lone 6*s*^2^ electrons may form localized active sites, while the 6*p* orbitals are involved in the charge transfer process. This mixed valence state characteristic (such as the coexistence of Bi^3+^ and Bi^0^) can dynamically regulate the surface charge distribution of the catalyst and promote the adsorption and activation of CO_2_. The Bi site stabilizes the CO_2_ intermediate through strong electron affinity, while the metallic property of Bi^0^ accelerates electron transport. (ii) High selectivity for C_1_ products due to favorable adsorption energies for CO_2_ intermediates. Bi-based catalysts have moderate adsorption energy for CO_2_ reduction intermediates (such as *OCHO^−^, COOH), and tend to generate C_1_ products such as formic acid (HCOOH) or CO [34], rather than polycarbon products (such as ethylene, ethanol). This is due to the relatively weak binding strength between Bi and the intermediate, avoiding the difficulty of C–C coupling caused by excessive reduction. (iii) Stability under reduction conditions. Bi-based catalysts can still maintain structural stability at the reduction potential [35], which is related to their corrosion resistance and dynamic surface reconstruction ability. For example, Bi_2_O_3_ may partially transform into Bi^0^ during the reduction process [36], but the Bi–O–Bi bridge bonds formed on the surface can inhibit excessive reduction. Furthermore, the low toxicity of Bi makes it suitable for long-term operation without the need for frequent catalyst replacement [37, 38]. (iv) Good compatibility with photoelectrocatalytic systems. Bi-based semiconductors have an appropriate bandgap width and can absorb visible light to drive photocatalytic CO_2_ reduction [39]. Meanwhile, its high carrier mobility enables it to perform excellently in electrocatalysis.

The structural characteristics of the material determine its catalytic properties. For the Bi-based photoelectrocatalysts, based on the recent reported literature, key factors influencing their performance typically include surface structure, oxidation state, and coordination environment. The surface structure directly affects the kinetic process of catalytic reactions through geometric and electronic effects. Nanosheets, porous structures, or hierarchical structures can significantly increase the specific surface area and expose more active sites [40]. The differences in surface energy among different crystal planes can also lead to different adsorption capacities of reactants [41]. The presence of vacancies can act as electron traps, inhibiting the recombination of photogenerated carriers, thereby further affecting the catalyst activity [42]. In addition, the oxidation state of Bi directly affects the energy band structure and REDOX capacity of the material [43]. The high-valent Bi^3+^ can enhance the oxidation capacity of the catalyst and generate more holes, but may sacrifice the light absorption range. An excessively high Bi^0^ ratio can also lead to metal agglomeration and reduce stability. The coexistence system of mixed valence states can form an internal electric field, accelerate the separation of electrons and holes, and effectively enhance the CO_2_RR activity [44]. Besides, the coordination environment, such as the type, number, and spatial arrangement of coordination atoms/ligand, determines the electron distribution of the active center. For instance, the electronegativity of coordination atoms affects the electron supply [45], and the coordination with different halogen atoms regulates the product distribution [46], thereby influencing the CO_2_RR rate as well as the yield and selectivity of the target product.

**Scheme 1:**
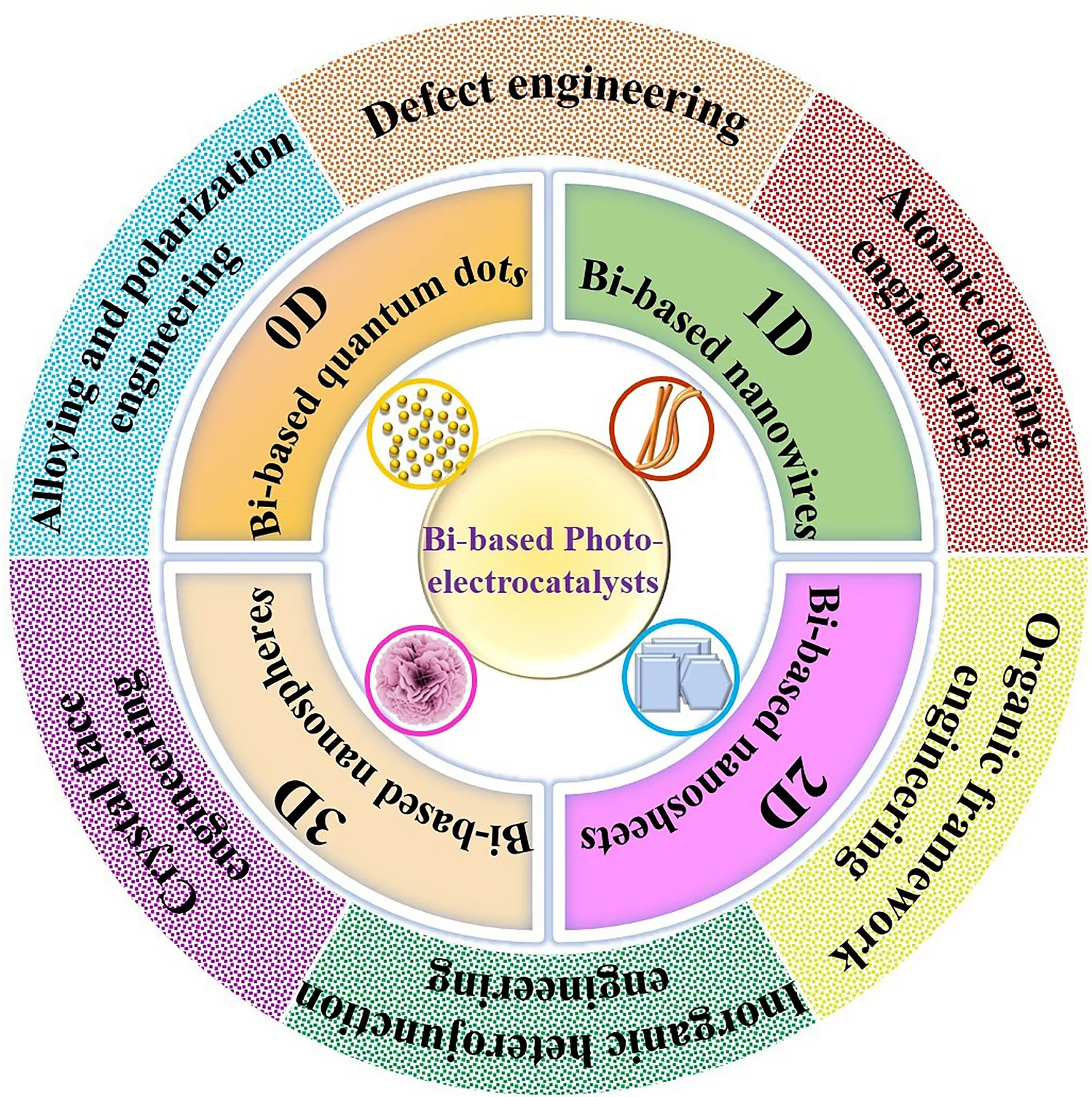
Recent major regulation strategies of Bi-based photoelectrocatalysts, which can be typically divided into six categories: defect engineering, atomic doping engineering, organic framework engineering, inorganic heterojunction engineering, crystal face engineering, and alloying and polarization engineering

Although Bi-based catalysts have many of the excellent photoelectric properties described above, several challenges persist in practical large-scale applications. Firstly, Bi-based photoelectrocatalysts can simultaneously catalyze multiple reactions [47] in the process of CO_2_RR, leading to poor product selectivity. For instance, in the electrocatalytic route, the side reaction of the hydrogen evolution reaction [48, 49] (HER) except CO_2_RR also occurred, which competed with CO_2_RR to generate hydrogen (H_2_) and reduced the efficiency of CO_2_RR. Secondly, the catalytic efficiency of these Bi-based catalysts may also be constrained by reaction conditions [50], such as temperature, pressure, and catalyst concentration. For instance, under low temperature and pressure, the activation energy barrier of CO_2_ molecules increases, leading to the blocking of adsorption and cleavage (such as C=O bond breakage); and then, the formation rate of *CO_2_^−^ on the surface of Bi-based catalyst surface significantly decreases, which directly affects the path of subsequent protonation to form HCOOH or CO [51, 52]. Thirdly, but definitely not least, is the structural properties to be improved of Bi-based catalyst itself, such as particle size, morphology, crystallinity, oxidation state, and interfacial microenvironment [25, 26], which will directly determine its catalytic properties. For instance, some Bi-based photoelectrocatalysts have a wide band gap in photocatalyzed CO_2_RR, which means that they can only absorb specific wavelengths of light and cannot effectively utilize most of the visible light in sunlight, significantly hampering their photocatalytic efficiency [53]. Also, the insufficient active sites, the slower rate of electron transfer, and the poor surface microenvironment will lead to a decrease in selectivity and current density. In a word, compared to the single structure of its pure phase, the improved Bi-based catalysts with many excellent structural characteristics contribute to enhanced performance. Therefore, it is very important to improve the multiple structural features by some rational modulation method. Recently, researchers have developed many modification strategies [54–57] in Bi-based catalysts to increase the catalyst’s active site [58–60], accelerate the electron transport rate [61], enlarge the specific surface area [62], and finally improve the selectivity and catalytic performance of CO_2_ reduction, which were well summarized and are presented in Scheme 1.

To our best knowledge, in the previous review articles, most researchers have either reported regulatory strategies for a specific class of Bi-based catalysts, such as Bi_2_Sn_2_O_7_ [63], Bi_2_WO_6_ [64], Bi-based metal–organic frameworks (MOFs) [65],or have focused on a specific class of CO_2_RR processes, such as photocatalytic CO_2_RR [66, 67] and electrocatalytic CO_2_RR [68]. Compared with the current existed reviews, in this paper, from the perspective of structure improvement and performance control, six main structural regulation strategies of various Bi-based catalysts for the photocatalytic and electrocatalytic CO_2_RR in recent years are summarized comprehensively, which can be divided into defect engineering, atomic doping engineering, organic framework engineering, inorganic heterojunction engineering, crystal surface engineering, alloying, and polarization engineering. Furthermore, the corresponding catalytic mechanisms for each regulation strategy will also be discussed in detail, which provides materials and chemistry researchers with new insights into how to improve the activity, selectivity, and stability of Bi-based catalysts. In addition, based on the current development of Bi-based catalysts in the field of photoelectrocatalytic CO_2_RR applications, the challenges and future opportunities will be featured in this paper. Three reasonable prospects or important research directions worthy of further in-depth study will be proposed, that is, the combination or synergy of multiple regulatory strategies, revealing the formation mechanism and realizing controllable synthesis, and in situ multiscale investigation of activation pathways and uncovering the catalytic mechanisms, aiming to enable researchers to understand the structure–property relationship of catalysts, realizing the controllable synthesis and performance regulation of Bi-based catalysts and even other catalysts.

## Regulation Strategies and Mechanisms

Compared to the counterpart of Bi-based catalysts with a pure phase perfect structure, their CO_2_RR performance could be further improved to meet the needs of industrial applications by reasonable means of modification. In this section, we summarize the latest and representative regulation strategies in recent years. That can be mainly divided into the following six categories: (1) defect engineering, (2) atomic doping engineering, (3) organic framework engineering, (4) inorganic heterojunction engineering, (5) crystal face engineering, and (6) alloying and polarization engineering. Meanwhile, the corresponding catalytic mechanisms of each regulation strategy will also be discussed in detail, aiming to enable researchers to understand the structure–property relationship of the improved Bi-based catalysts fundamentally.

### Defect Engineering

**Fig. 1:**
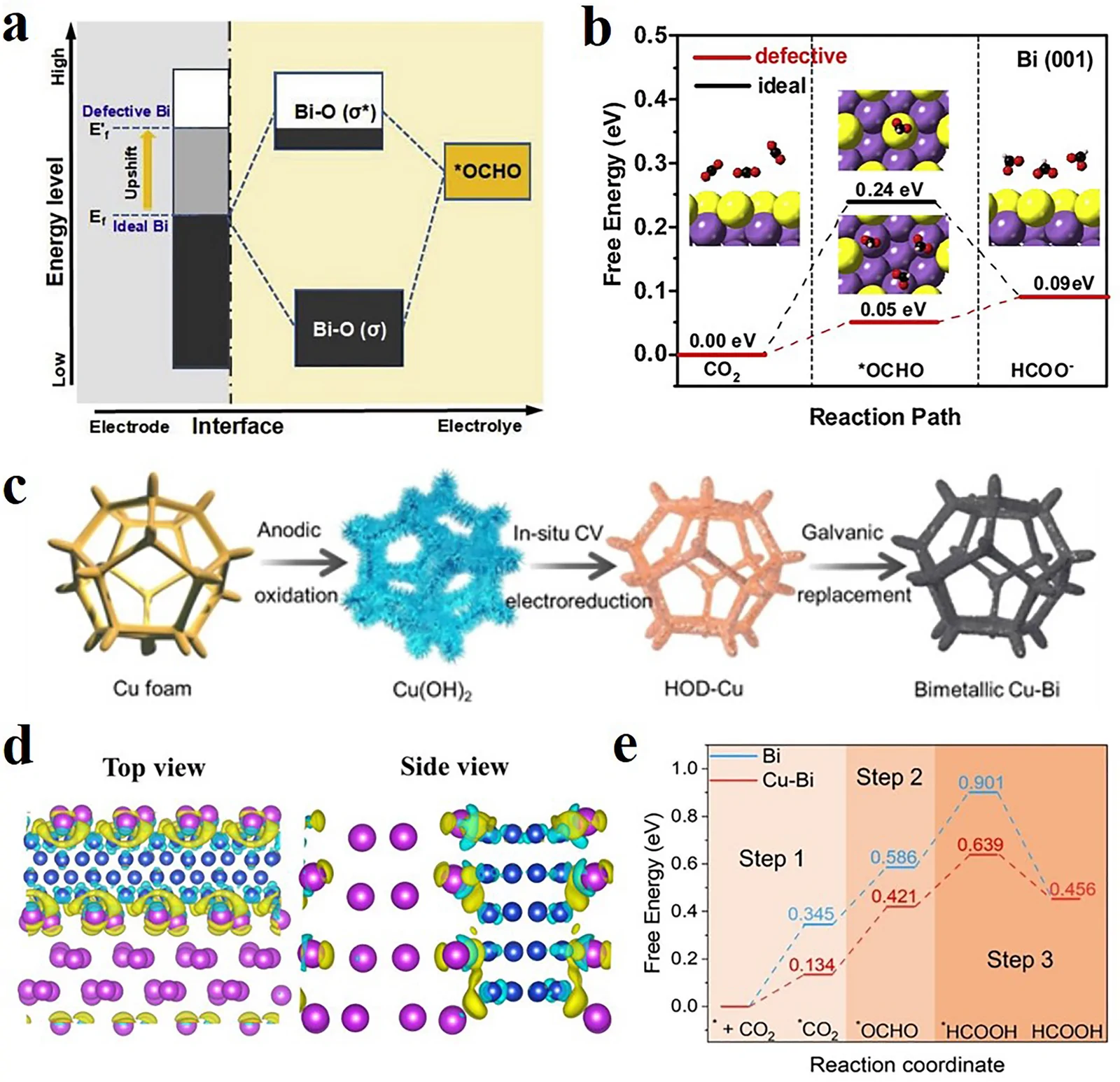
Regulation mechanism of atomic Bi vacancy on the activity and reaction path of CO_2_RR. **a** Schematic diagram of upshifting Fermi level by forming atom vacancies in Bi catalyst to decrease the CO_2_RR overpotentials. The dot-dash line represents the interface between Bi electrode and electrolyte; **b** free energy for *OCHO generation on ideal and defective Bi (001) surfaces [71]; © 2020 Elsevier B.V. **c** A schematic illustration for synthetic procedure of bimetallic Cu–Bi nanostructures; **d** charge density of bimetallic Cu–Bi. The yellow area represents the accumulation of electrons, and the cyan area represents the reduction of electrons; **e** Gibbs free energy profiles of formate production pathways on pure Bi and bimetallic Cu–Bi [72]. © 2023 Wiley–VCH GmbH

The first and foremost strategy is defect engineering. Recently, it was found that designing defects [69] may be an effective way of regulating the physical, chemical, and electronic properties of catalysts, thereby improving the catalytic activity of the catalyst. Intrinsic defects have a significant effect on the electronic structure and surface atomic structure of Bi-based photoelectrocatalysts, which in turn determines the charge separation and transport process [70]. Also, the defect engineering of the material can regulate the energy difference between the antibonding state and the Fermi level, thus strengthening the chemical bond of the adsorbed material on the surface and promoting a stable electrochemical reaction. Atomic vacancy and surface electron-rich properties are important for CO_2_ activation. The CO_2_ conversion efficiency can be significantly improved by using an electrochemical reduction method to prepare Bi nanosheets with a specific structure. Zhao et al. [71] proposed an electrochemical topological phase transition method for the electrochemical reduction of layered Bi_2_O_2_CO_3_ to Bi nanosheets with (001) dominant surfaces and atomic vacancies. As shown in Fig. 1a, b, density functional theory (DFT) calculation confirmed that the atomic vacancy of Bi induced the formation of an electron-rich surface, leading to the shift of the *P-state* to Fermi level, reducing the activation energy of CO_2_ to CO_2_* radical, and promoting the stability of OCHO* intermediate through the *p* orbital hybridization between the O and Bi electrodes of the carbon-containing intermediate. The conversion rate of CO_2_ to HCOOH is greatly improved. To achieve high selectivity, high activity, and high stability of CO_2_RR to HCOOH, Cheng et al. [72] also used bimetallic Cu–Bi to transfer electrons from Cu donor to Bi acceptor, thus forming an electron-rich Bi nanocatalyst, as shown in Fig. 1c. The charge density distribution of bimetallic Cu–Bi illustrated in Fig. 1d demonstrates that the charge density is depleted around the central Cu atom. The accumulation around adjacent Bi atoms indicates that electrons are continuously transferred from Cu atoms to Bi atoms, resulting in the formation of electron-rich Bi atoms. Figure 1e shows the Gibbs free energy spectrum of the formic acid synthesis pathway of pure Bi and bimetallic Cu–Bi. In the first step, the free energy of *CO_2_ formation on bimetallic Cu–Bi (0.13 eV) is much lower than that of pure Bi (0.35 eV) to confirm that the bimetallic Cu–Bi catalyst can adsorb CO_2_ well on electron-rich Bi. In addition, bimetallic Cu–Bi (0.42 eV) has a lower reaction free energy of *OCHO intermediates than pure Bi (0.59 eV), indicating that electron-rich Bi (bimetallic Cu–Bi) catalysts are more favorable to *OCHO intermediates than electron-neutral Bi (pure Bi) catalysts. Therefore, the reasonable construction of Cu–Bi bimetallic electrocatalyst can form an electron-rich Bi active site, promote the activation of CO_2_ molecules, enhance the adsorption strength of *OCHO intermediates, and finally contribute to a superior CO_2_RR performance in the formation of HCOOH. Thus, from the point of view of an electron-rich surface, it provides us with a new idea for the development of efficient and highly selective CO_2_ conversion Bi-based catalysts.

**Fig. 2:**
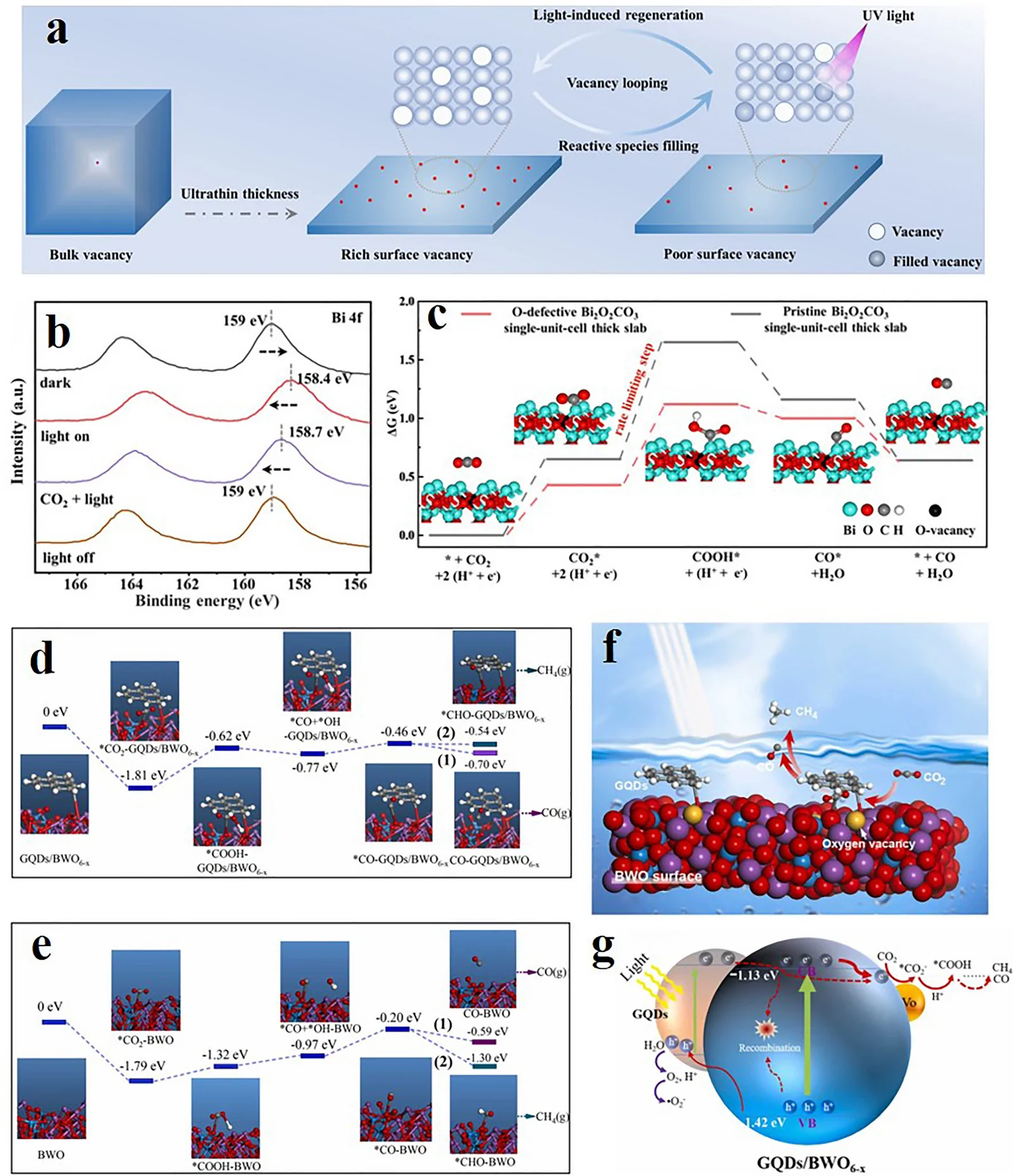
Fabrication, characterization, and reaction pathways of oxygen vacancy (Vo). **a** Synthesis of renewable oxygen vacancy defect single-cell BOC nanosheets by ultraviolet irradiation; **b** Quasi in situ XPS spectra of Bi 4f for the UV-10-BOC nanosheets.; **c** calculated free energy diagrams and schematic illustration of CO_2_ photoreduction to CO [75]. © 2021 Wiley–VCH GmbH. **d**, **e** Free energy diagrams for the reduction of CO_2_ to CO or *CHO over GQDs/BWO_6-x_ and BWO; **f** illustration of GQDs/BWO_6-x_ surface microstructure; **g** illustration of a possible mechanism for photocatalytic CO_2_RR of GQDs/BWO_6-x_ [76]. © 2021 Elsevier B.V. All rights reserved

Oxygen vacancy (Vo) [73], as the most thermodynamically stable anionic defect type in metal–oxide surface systems, has an extensive research basis in materials science. Experimental characterization and theoretical calculation results show that the defect state can introduce intermediate energy levels into the band gap of the material and significantly regulate the optical absorption band gap to the visible–infrared region. At the same time, the localized defect state formed by the surface Vo [74] can be used as an effective carrier trapping center, and the interface charge separation efficiency of the Bi-based semiconductor material can be significantly improved by inhibiting the photogenerated electron–hole pair recombination kinetic process, thus enhancing its photocatalytic reaction activity. Sun’s group [75] designed a method of fast low-voltage ultraviolet irradiation and successfully synthesized defective single-cell Bi_2_O_2_CO_3_ (BOC) nanosheets with renewable oxygen vacancies, as shown in Fig. 2a. It was found that under the condition of visible light irradiation, the O defect sites in BOC nanosheets significantly enhance the ability to capture photoexcited electrons, and then transfer these electrons to highly active CO_2_ molecules shown in Fig. 2b. Compared with untreated BOC nanosheets, the formation of COOH* intermediates is significantly reduced, from 1.64 to 1.13 eV, as shown in Fig. 2c. The study revealed that the oxygen defect site can effectively enhance the photoexcited electron capture ability and decrease the COOH* intermediate formation energy, which has important implications for understanding the reaction mechanism and improving the catalytic efficiency. Besides, Xiong et al. [76] prepared a graphene quantum dots (GQDs) modified Vo-containing Bi_2_WO_6_ (BWO) composite (GQDs/BWO_6-x_) by microwave-assisted hydrothermal method and used it for photocatalytic CO_2_RR. Based on the DFT calculation results shown in Fig. 2d and e, GQDs/BWO_6-x_ has a lower energy barrier (0.16 eV) than Bi_2_WO_6_ (1.12 eV) for converting intermediate product *COOH into *CO. Through the introduction of oxygen vacancy defects, the photogenerated carrier presents a spatial localization distribution, which significantly reduces the probability of carrier recombination and effectively improves the separation efficiency of photogenerated electron–hole pairs. Furthermore, the surface modification of GQDs significantly broadened the absorption range of the composite in the visible light band (400 ~ 800 nm). It is worth noting that the synergic modification of GQDs and Vo causes a significant shift in the conduction band position of BWO, and the conduction potential of Bi_2_WO_6_ negatively shifts from −0.12 eV of the original sample to −1.13 eV (GQDs/Bi_2_WO_6-x_). This optimization of the band structure makes the composite system exhibit a stronger reduction potential, which provides more favorable thermodynamic conditions for photocatalytic CO_2_RR. According to these results, a potential reaction mechanism for photocatalytic CO_2_RR was proposed and shown in Fig. 2f and g. When exposed to light, both BWO and GQDs release a large number of electrons, and these photogenerated electrons tend to migrate to neighboring atoms of Vo, thus accelerating the charge separation process. The introduction of GQDs makes the conduction band of BWO more negatively charged, which significantly improves the material's response to visible light [77]. The increase in the negative carbon black content in the composite is conducive to improving its overall reduction performance [78]. The active sites constructed by surface oxygen vacancies dominate the chemisorption and activation processes of CO_2_ molecules. Theoretical calculation and experimental characterization confirmed that CO_2_ molecules preferentially adsorbed in the surface local electron state region induced by Vo, forming the chemisorbed CO_2_ intermediate species. Under the condition of photoexcitation, the photogenerated electrons are effectively captured by the defect state and injected into the adsorptive CO_2_ molecular orbital, which triggers the single electron reduction reaction to produce *CO_2_^⁻^ active intermediates. At the same time, photogenic holes continuously provide a proton source (H^+^) by driving the water oxidation reaction and participate in the subsequent carbon chain remodeling process in synergy with the superoxide radical (O_2_^⁻^). Specifically, *CO_2_^⁻^ intermediates undergo a multistep proton-coupled electron transfer (PCET) reaction and gradually transform into key intermediates such as *COOH and *CHO. Finally, through surface catalytic steps such as C–O bond breaking and C–H/O–H bond formation, the selective generation of CO, CH_4_, and other carbon-based fuel products is realized. The distribution of the products is coordinated by the electronic structure of the catalyst surface and the reaction kinetics.

**Fig. 3:**
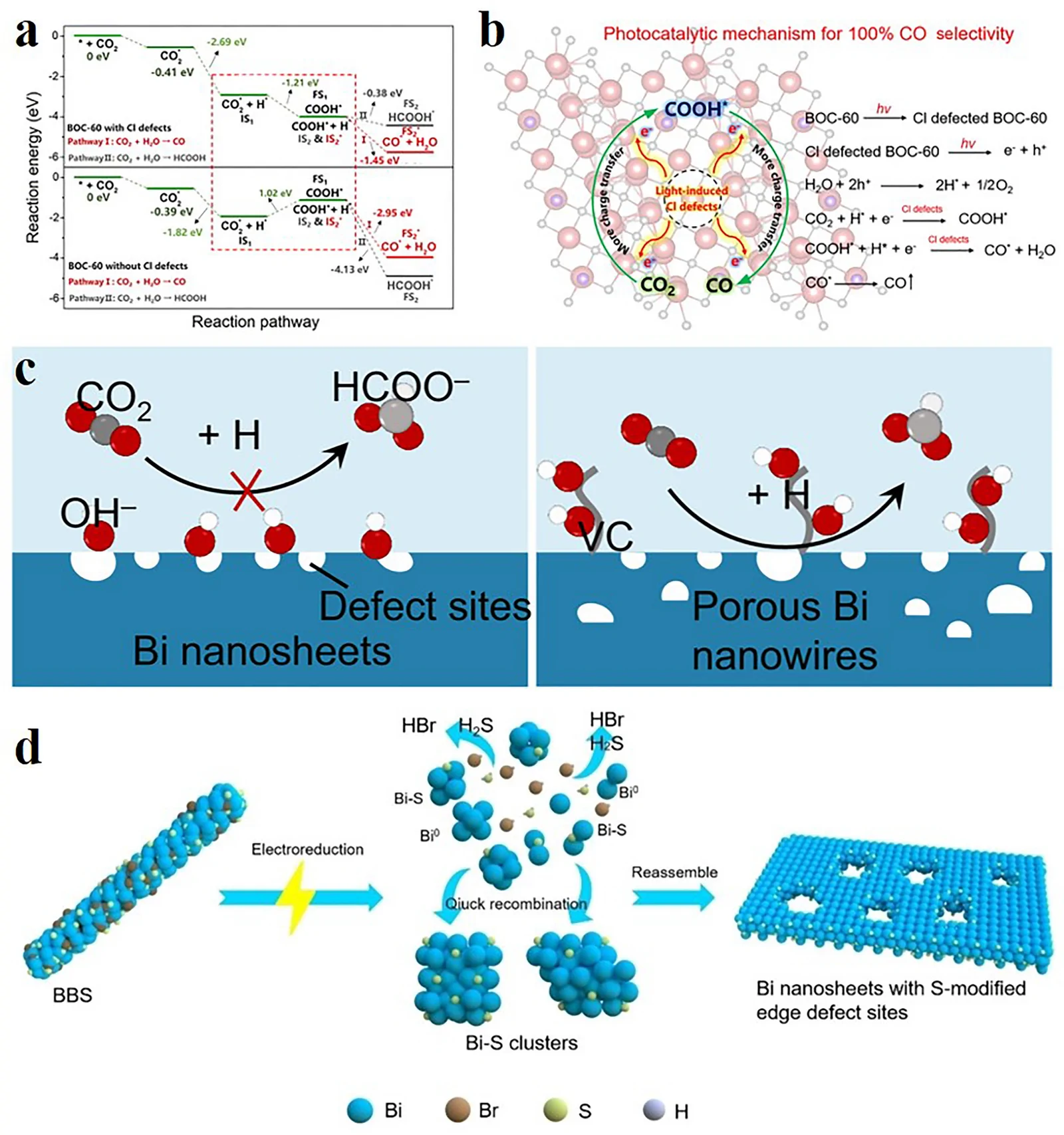
Other strategies of modulating the defect sites with nonmetallic elements (Cl, S, et al.). **a** Reaction pathway for photocatalytic CO_2_ reduction over Bi_5_O_7_Cl based on DFT calculation; **b** photocatalytic CO_2_ reduction mechanism promoted by light-induced Cl defects [79]. Copyright © 2022 Science China Press. **c** Schematic illustration of the hydroxyl trapping by VC passivate layer and ensure a smooth CO_2_ reduction at the defective sites [81]. Copyright © 2023, Crown. **d** Schematic diagram of the structural evolution from BBS to Bi nanosheets with S-modified edge defect sites [82]. Copyright © 2023 Wiley‐VCH GmbH

The defect engineering strategy has also made great progress in photocatalytic CO_2_RR of Bi-based halides. The photodynamic defect source in the halide perovskite system is the synergistic effect of ion dissociation migration and lattice relaxation under photoexcitation. This metastable defect structure usually leads to the carrier loss mechanism dominated by non-radiating composite channels, which seriously restricts the energy conversion efficiency. Shi’s group [79] successfully constructed a Bi_5_O_7_Cl photocatalytic material system with temperature gradient regulation by implementing the in situ defect engineering strategy through a controlled thermal precipitation method. The results showed that the sample synthesized at 60 °C could maintain the stable existence of high concentration dynamic Cl vacancy (V_Cl_) under continuous light, and its photocatalytic CO_2_ reduction activity reached the optimal value. (The yield was 5.8 times higher than that of the intrinsic sample.) It is revealed that photodynamic halogen defects form an active site network for electron–hole separation by reconstructing the surface coordination environment in the Bi–O–Cl terpolymer system. As shown in Fig. 3a, b, the Cl defect reduces the energy barrier of this step and enables the spontaneous generation of CO_2_* to COOH*. This is because the CO_2_* absorbed on the Cl defect is continuously reduced by the incident electrons transferred from the defect position with abundant local electrons on the surface, thus promoting the continuous protonation process, optimizing the reaction kinetic path for the formation of the *CO intermediate, and ultimately reducing it to CO with 100% selectivity. Similarly, Guan et al. [80] prepared Bi_12_O_17_Cl ultra-thin nanosheets and Bi clusters with Vo by a simple one-step hydrothermal method. Vo-rich Bi–Bi_12_O_17_Cl ultra-thin nanosheets have a better effect on the photocatalytic activity of CO_2_ reduction, and the precipitation rate of CO is 28 times that of Bi_12_O_17_Cl. The presence of Vo and Bi clusters leads to the spatial separation of photogenerated carriers. Furthermore, Vo-rich Bi–Bi_12_O_17_Cl ultra-thin nanosheets have various configurations, such as light absorption capacity, efficient separation of spatial carriers, and optimal adsorption and activation of CO_2_ molecules, which make them excellent catalysts for highly selective photoreduction of CO_2_ to CO.

Besides, researchers also proposed other ways for modulating the defect sites in the electrocatalytic CO_2_RR process for further improving the catalytic performance of Bi-based catalysts and satisfying their industrial applications. For instance, achieving high formic acid yields at wide voltage intervals and industrial current densities remains difficult because Bi catalysts are often poisoned by oxygen-containing substances. Zhu et al. [81] reported a Bi_2_S_3_ nanowire ascorbate hybrid catalyst. By introducing a molecular passivation layer of vitamin C (VC), as shown in Fig. 3c, the reconstruction process of the Bi-based catalyst was reasonably regulated to prevent hydroxyl poisoning from generating high-activity defect sites, and then achieving high formate yield at industrial ampere-level current density. Also, Lv et al. [82] synthesized Bi nanosheets with S-modified edge defect sites by electrochemical reconstruction of Bi_19_Br_3_S_27_ nanowires (BBS) shown in Fig. 3d. The results showed that the S dopant was mainly located at the edge of Bi nanosheets, and had a strong adsorption capacity for *OCHO intermediates, which reduced the density of the complex unsaturated Bi site and thus the adsorption density of *H, and finally effectively inhibited the generation of H_2_ and CO in the electrocatalytic CO_2_RR process.

The defect engineering strategy significantly enhances the activity and selectivity of the CO_2_RR by regulating the electronic structure of Bi-based catalysts, the adsorption energy of intermediates, and the carrier separation efficiency. For instance, atomic vacancies (such as Bi vacancies) and Vo optimize the HCOOH generation path by forming local electron-enriched regions, reducing the energy barrier for CO_2_ activation and stabilizing key intermediates (such as *OCHO). The electron-rich Bi sites formed by charge transfer in the bimetallic Cu–Bi system enhance both activity and selectivity simultaneously through a synergistic effect. However, this strategy have limitations: (1) high-density defects may lead to intensified carrier recombination or a decline in structural stability. For instance, the instability of dynamic halogen vacancies (such as Cl vacancies) under light excitation may cause catalyst deactivation; (2) selective enhancement often comes at the expense of a wide potential window or the entire reaction kinetics. For instance, although sulfur-modified edge defects suppress hydrogen evolution side reactions, they may limit the multielectron path extension of CO_2_ reduction; (3) there is a lack of systematic research on the long-term tolerance of catalysts (such as antitoxic ability) in defect engineering, especially at industrial-grade current densities, defect sites are prone to being occupied by intermediates or structurally reconstructed. In addition, the synergistic effects of different defect types (such as cationic vacancies and anionic vacancies) have not been fully explored. For instance, the coupling of Vo and quantum dot modifications can simultaneously broaden light absorption and optimize the band structure, but the interface charge transport mechanism still requires in-depth analysis. In the future, it is necessary to balance the defect concentration and distribution at the atomic scale and reveal the true role of defects in dynamic reactions, to achieve multidimensional optimization of activity–selectivity–stability.

### Atomic Doping Engineering

**Fig. 4:**
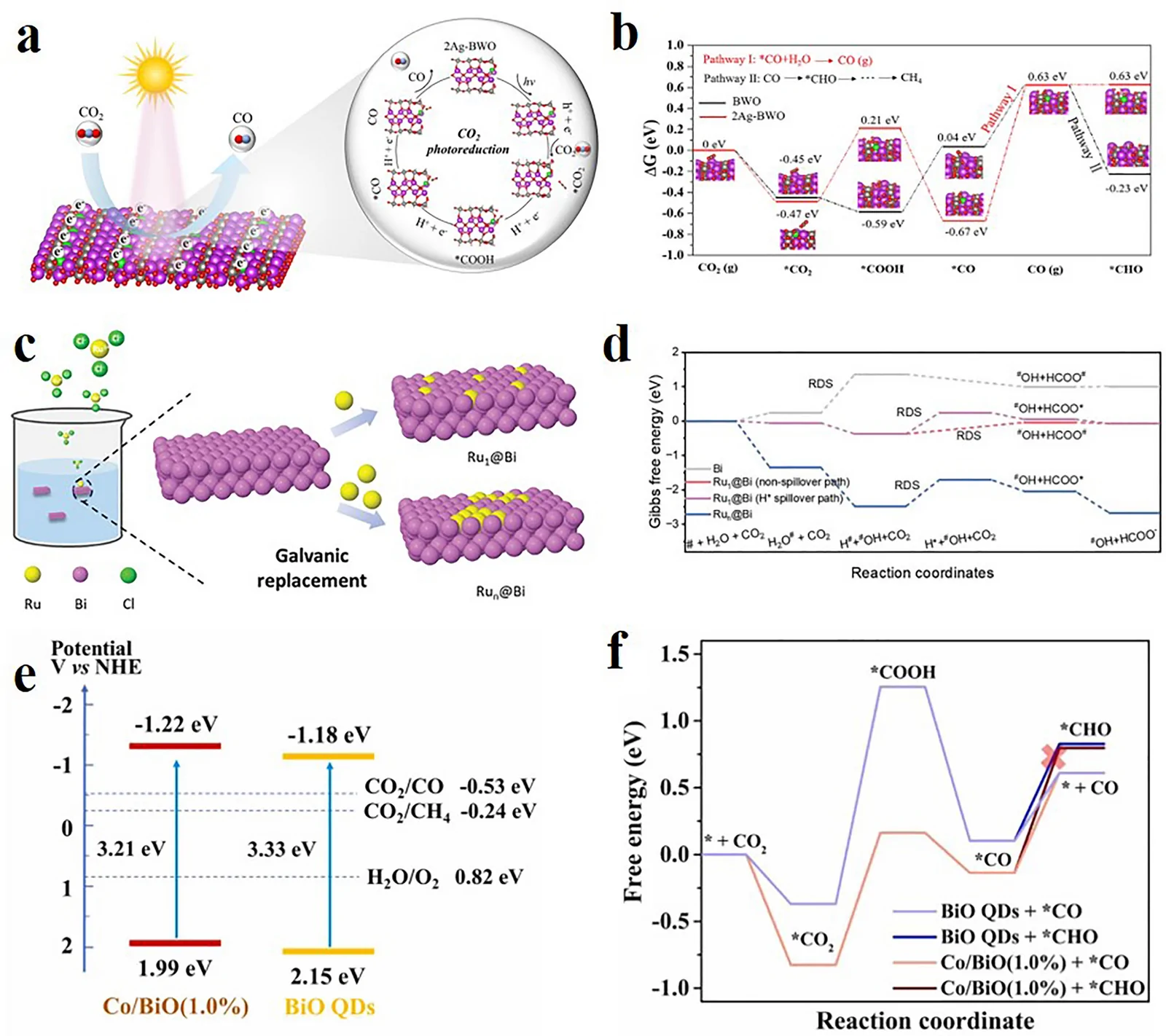
Atomic doping strategy optimizes the reaction pathways of Bi-based photoelectrocatalysts. **a** Schematic of reaction mechanism on ultra-thin Ag–BWO nanosheets for photoreduction of CO_2_ to CO; **b** DFT-calculated Gibbs free energy of reaction intermediate on BWO and Ag–BWO with related geometry structures for CO_2_ photoreduction [83]. © 2023 Elsevier B.V. **c** Schematic representation of the preparation of Ru_1_@Bi and Ru_n_@Bi catalysts; **d** Gibbs free energy landscapes for CO_2_ reduction to formate at 0 V versus RHE. The RDS is labeled with a black text [84]. © 2024 Wiley‐VCH GmbH. **e** Band structure alignments of BiO QDs and Co/BiO(1.0%); **f** calculated free energy diagrams for CO_2_ reduction into CO on BiO QDs and Co/BiO(1.0%) [85]. © 2023 Elsevier BV

It is worth noting that regulating the intrinsic properties of catalysts by defect engineering does not exist in isolation. The synergistic effect is formed by atomic doping engineering, which reconstructs the energy band structure of materials at the molecular level by introducing heterogeneous elements, providing another dimension for optimizing charge dynamics and creating new active sites. Atomic doping engineering can be divided into metal atom doping, nonmetal atom doping, and co-doping. Atomic doping is a simple and effective method to regulate the electronic structure and surface-active site of catalysts. The introduction of heteroatoms can change the energy band structure, regulate the conductivity and light absorption characteristics, promote the effective separation of charge, reduce the electron–hole pair recombination, and introduce new active sites of the Bi-based catalysts, thereby improving their catalytic activity. For metal atom doping, Zhao et al. [83] successfully synthesized Ag–Bi_2_WO_6_ (Ag–BWO) photocatalyst by using hydrothermal method and liquid-phase cation exchange process to precisely replace Bi^3+^ with Ag^+^ in ultra-thin Bi_2_WO_6_ nanoplates, as shown in Fig. 4a. The catalyst has abundant surface-active sites, adjustable band locations, enhanced CO_2_ and H_2_O molecular absorption capacity, and excellent charge separation efficiency. Ag^+^ doping induces a significant electronic restructuring effect in the BWO lattice, achieves high-speed directional migration of photogenerated carriers by constructing Bi–O–Ag charge transfer channels, prolongs the carrier lifetime up to 3.2 times that of the intrinsic sample, and significantly improves the quantum efficiency of the photocatalytic system. DFT calculations show that the partial substitution of the Bi^3+^ site by Ag reduces the adsorption energy of CO_2_ molecules from − 0.45 to − 0.82 eV. At the same time, the activation energy barrier of the C=O bond drops by about 0.37 eV. This thermodynamic advantage significantly promotes the chemisorption and activation of CO_2_ molecules on the catalyst surface. Notably, the upward shift of the valence band position (+ 0.23 eV) induced by Ag doping optimizes the kinetic matching of the water oxidation reaction, ensuring a continuous supply of proton–electron pairs. Other experiments results confirm that photogenerated electrons were injected into the antibonding orbitons of CO_2_ molecules to form CO_2_^⁻^ intermediates, which were gradually transformed into COOH key intermediate species through the PCET process, and the C–OH bond breaking energy barrier of the photogenerated electrons was about 0.15 eV lower than that of the intrinsic samples. Finally, the target product CO is produced by desorption of the *CO intermediate state. The corresponding multistep reaction path is shown in Fig. 4b. Liu et al. [84] adopted Ru-doped Bi as a model system and synthesized two kinds of Bi-based catalysts of Ru_1_@Bi and Ru_n_@Bi, as shown in Fig. 4c. X-ray absorption near-side structure (XANES) and extended X-ray absorption fine structure (EXAFS) measurements were used to probe the chemical state and local coordination environment. It was found that the oxidation states of Ru atoms in Ru_1_ @Bi and Ru_n_@Bi are + δ and + ε (0 < ε < δ < 3), and after the CO_2_RR test, their oxidation states cannot be retained, and the main form of Ru in the working catalyst is Ru^0^. The *k*^2^-weighted Fourier transform (FT)-EXAFS curve for Ru_1_@Bi has a peak at 1.53 Å, which is attributed to the Ru–O bond and consistent with the XANES result. No Ru–Ru bonds exist, confirming the presence of individually atomically dispersed Ru atoms. For Ru_n_@Bi, the bond length of the Ru–Ru is 2.40 Å, which confirms the existence of the Ru cluster. Combined with the above results, it is proved that the isolated Ru single atoms and clusters are successfully fixed on the Ru_1_@Bi and Ru_n_@Bi Bi substrates, respectively. As shown in Fig. 4d, doping Ru atoms in Bi significantly reduces the reaction energy barrier, promotes H_2_O dissociation and H* migration to the Bi site, thereby converting CO_2_ into formate, and constructs Ru–Bi bridge sites through non-overflow paths to achieve efficient CO_2_ hydrogenation. Compared with pure Bi- and Ru-cluster-doped Bi, Ru-single-atom-doped Bi exhibits nearly twice the high current density, which significantly enhances the catalytic efficiency. Xu et al. [85] obtained BiO quantum dots (QDs) containing Co single atoms through a simple one-step hydrothermal reaction. The resulting products were labeled as BiO QDs, Co/BiO (0.5%), Co/BiO (1.0%), Co/BiO (2.0%), and Co/BiO (3.0%) according to the molar ratio of Co to Bi. The single Co atoms achieve atomic-level dispersion (Co-SAs/BiO) in the second coordination layer of the BiO quantum dots and induce a unique orbital hybridization mechanism by restructuring the coordination microenvironment. EXAFS and electron energy loss spectroscopy confirm that Co atoms preferentially occupy the next-nearest coordination sites of the Bi–O octahedron, and the unoccupied 3*d* orbitals form strong hybridization with the Bi 6*s*-O 2*p* orbitals, which significantly enhances the π-π reverse donation with the C 2*p* orbitals of CO_2_ molecule. Ultraviolet photoelectron spectroscopy (UPS) analysis shows that the orbital reconstruction can significantly reduce the work function of the BiO quantum dots, effectively reduce the Schottky barrier of photogenerated electrons migrating across the interface, and improve the charge transfer efficiency. As shown in Fig. 4e, the upward movement of the conduction band significantly enhanced the reduction potential of the catalyst, while the synchronous upward movement of the valence band reduced the oxidation potential and significantly improved the material stability. DFT calculation (Fig. 4f) further revealed that the Co site reduced its forming energy barrier by optimizing the adsorption configuration of *COOH intermediates while increasing the stabilization energy of the intermediates. This bifunctional regulatory mechanism enables Co-SAs/BiO (1.0%) to exhibit excellent photocatalytic efficiency and significantly increase CO production.

**Fig. 5:**
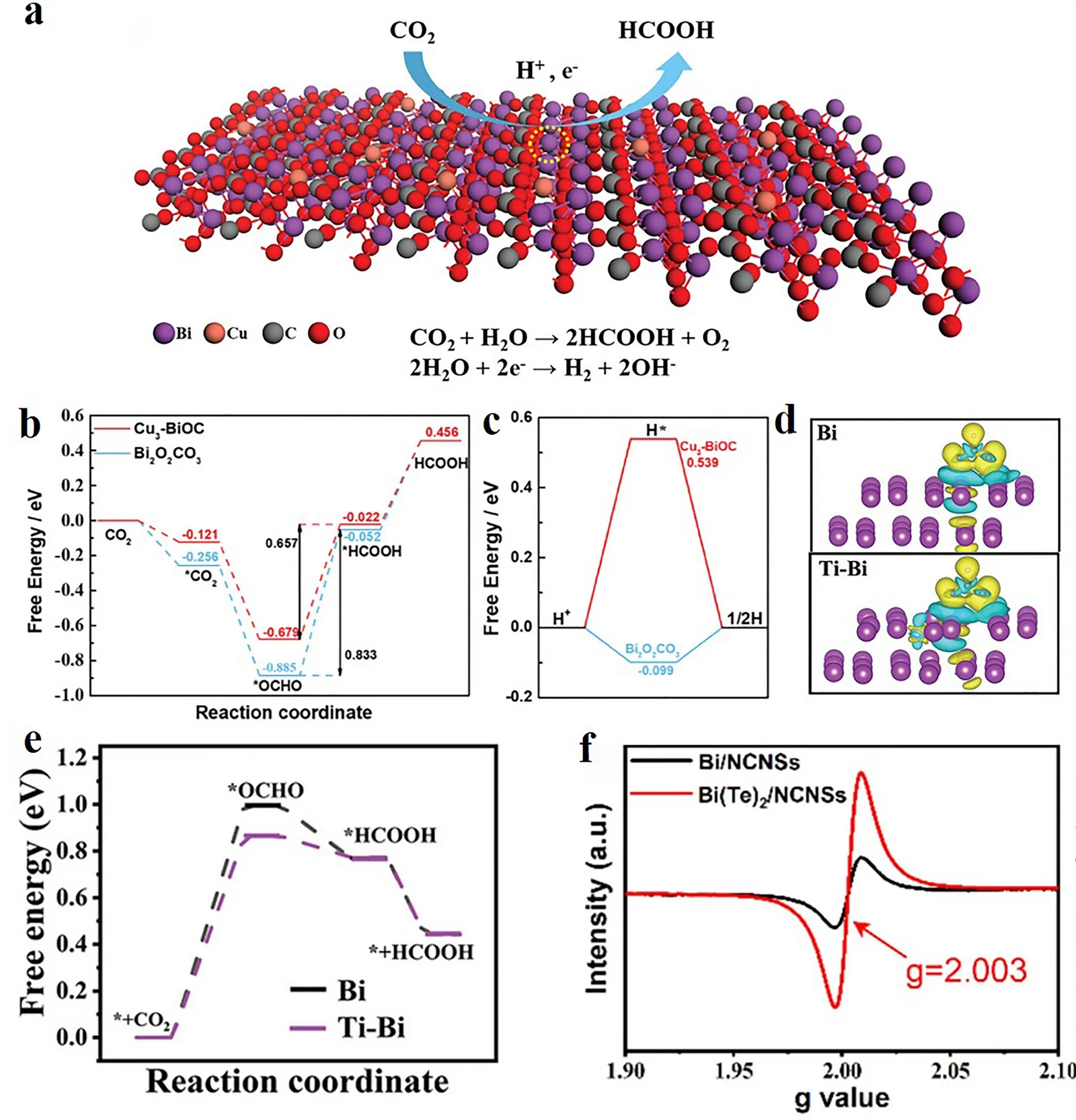
Doping-induced charge reconstruction and directional evolution of reaction pathways. **a** Catalytic process model of Cu–BOC; **b** Gibbs free energy profiles of formate production pathways on BOC and Cu–BOC; **c** Gibbs free energies for the formation of H* on BOC and Cu–BOC [86]. © 2024 Wiley–VCH GmbH. **d** The charge density difference between Bi and Ti–Bi, the Bi, Ti, C, H, and O atoms were illustrated in purple, blue, brown, white, and red, respectively; **e** free energy diagrams for CO_2_RR on Bi and Ti–Bi [87]. © 2023 Wiley–VCH GmbH; **f** EPR profiles of Bi(Te)_2_/NCNSs and Bi/NCNSs [90]. Copyright © 2022, American Chemical Society

As an electron hunter, the Cu atom was doped in BOC catalyst (Cu–BOC) for converting CO_2_ into formate, developed by Lu et al. [86], as shown in Fig. 5a. Cu–BOC catalyst with a hollow microsphere structure can prolong the residence time of CO_2_ on the catalyst and provide more active sites. DFT calculation showed that the presence of Cu significantly increases the charge density of the active site and affects the local electronic structure of Bi, thereby reducing the energy barrier associated with the conversion of *OCHO substances to formate (Fig. 5b, c). As a novel electrocatalytic CO_2_RR catalyst, Xu et al. [87] successfully synthesized Ti-doped Bi (Ti–Bi) nanosheets by one-step electrochemical reduction of Bi_4_Ti_3_O_12_. Differential charge density analysis (Fig. 5d) demonstrates that there is a significant electron redistribution phenomenon at the Ti–Bi interface, in which the yellow region (electron accumulation region) is mainly distributed around Bi atoms, while the blue region (electron depletion region) is concentrated near the Ti site. The Bader charge analysis showed that each Ti atom transferred 1.05 eV electron density to the neighboring Bi atom, and this strong electron transfer effect significantly increased the density of state of the active center of Bi. Figure 5e shows the free energy diagrams for CO_2_RR on Bi and Ti–Bi. Doping of Ti promotes the enrichment of Ti–Bi nanosheets and enhances the activation of CO_2_ molecules. The ultra-thin Ti–Bi nanosheets can also provide a large number of exposed active sites and accelerate mass transfer; meanwhile, its electron-rich nature can accelerate the production of *CO_2_, improve the adsorption strength of *OCHO intermediates, and thus improve the conversion of CO_2_ and the selectivity of formic acid. In addition, Zhang et al. [88] using Bi(NO_3_)_2_·5H_2_O and H_3_BTC as raw materials successfully prepared a composite catalyst with Ce-doped Bi^0^ nanoparticles confined to a porous carbon matrix by directly annealing the Ce-exchanged Bi–BTC precursor in an Ar atmosphere. The electrochemical tests showed that Ce doping significantly improved the electroreduction performance of the catalyst in the electrocatalytic CO_2_RR to formic acid. On the one hand, compared with traditional Bi^3+^-based catalysts, metallic Bi^0^-based catalysts exhibit excellent structural stability at cathodic reduction potential; on the other hand, Ce doping increased the electron density around Bi, significantly enhanced the adsorption strength of key intermediates *OCHO, and thus improved the selectivity and activity of CO_2_RR to HCOOH. In addition to the above several typical metal element doping (Ag, Cu, Co, Ce), to our best knowledge, semimetal elements can be also doped in Bi-based catalysts to obtain a functional enhancement catalyst. This is thanks to the partially filled d-band, which enables the semimetal-doped Bi-based catalysts to effectively participate in the CO_2_RR, especially in regulating the adsorption and reaction pathways of reaction intermediates [89]. Moreover, compared with precious metals or rare metals, the cost of many semimetallic materials is lower, which makes semimetal-doped catalysts more economical in industrial applications. Cui et al. [90] reported a semimetal-doped strategy, in which they prepared Te-doped Bi nanoparticles on ultra-thin N-doped carbon nanosheets (NCNSs) by in situ reduction. As shown in Fig. 5f, Te doping changes the electronic structure of Bi, reduces the oxidation state, and forms more oxygen vacancies. The adsorption of H_2_O molecules at the catalytic interface is also weakened. Weak water adsorption facilitates the formation of *OCHO intermediates while helping to inhibit HER, thereby improving the performance of CO_2_RR overall.

**Fig. 6:**
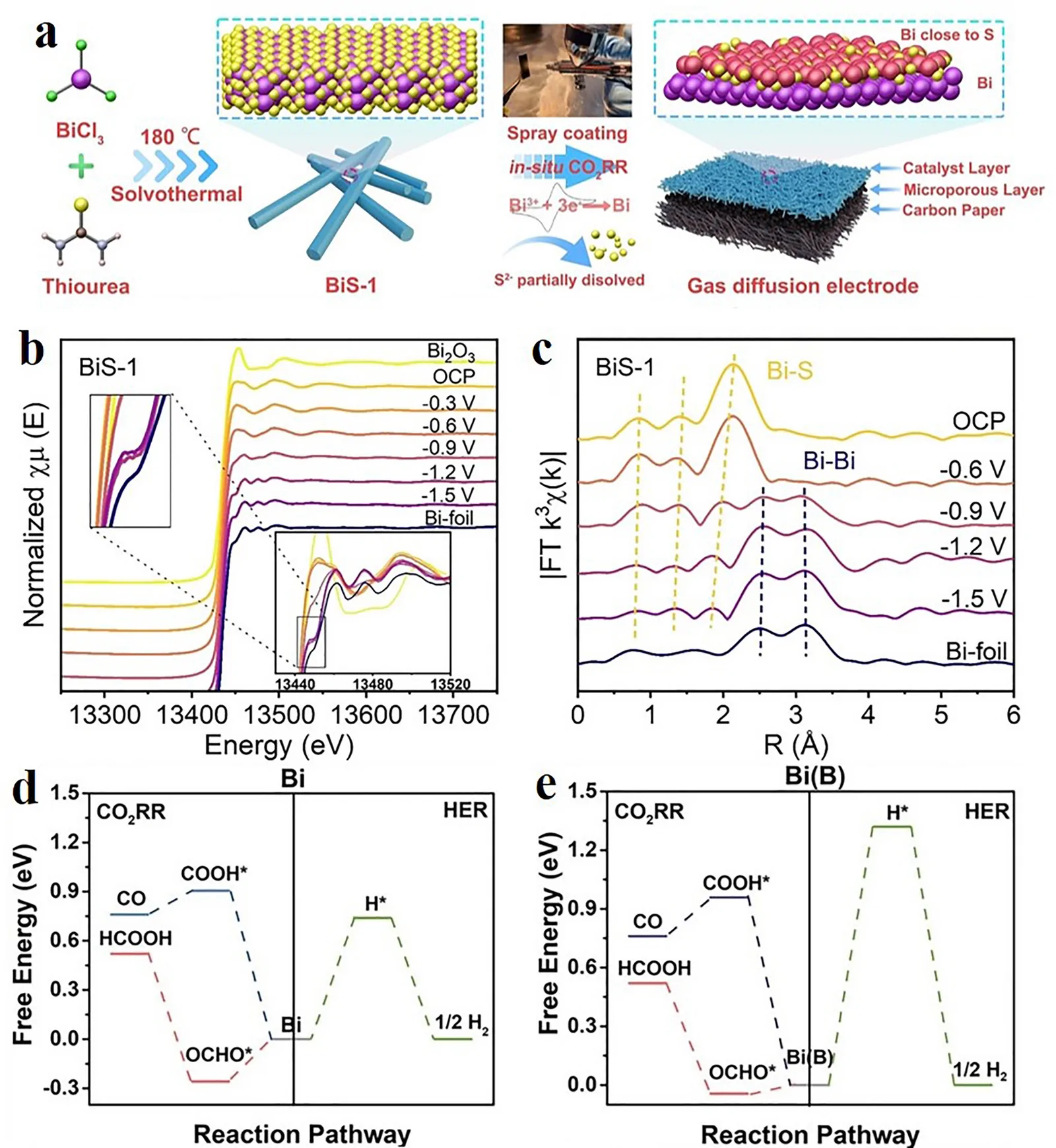
Structural evolution characterization and comparison of reaction energy barriers for doping catalysts. **a** Synthesis and reconstruction of BiS-1 nanorods catalyst; **b** normalized XANES and **c** FT-EXAFS of Bi L_3_-edge spectra for BiS-1 under CO_2_RR from OCP to -1.5 V. The in situ XANES tests are conducted in a customized three-electrode cell with CO_2_-filled 0.5 M KHCO_3_ [91]. © 2024 Wiley–VCH GmbH. Free energy diagrams of CO_2_RR and HER on **d** Bi surface and **e** Bi(B) surface [92]. © 2021 Wiley‐VCH GmbH

For nonmetal atom doping, Jiang et al. [91] designed an S-doped metallic Bi catalyst (BiS-1) through electrochemical construction, as shown in Fig. 6a, realizing the efficient generation of HCOOH in pH universal electrolyte with ampere-level current density. They used the in situ XANES technique to investigate in depth the evolution of the derived BiS-1 structures during CO_2_RR reconstruction. From Fig. 6b, in the process of CO_2_RR, with the increase in the potential, the absorption edge position of the catalyst gradually moves toward that of Bi foil, indicating that Bi^3+^ is reduced to Bi^0^. At high potentials, the near-edge structure of the catalyst is still higher than that of the Bi foil, implying partial precipitation of S atoms during the catalytic process. When the potential reaches -1.2 V, the structure tends to be stable. FT-EXAFS analysis (Fig. 6c) further found that the intensity of the Bi–Bi coordination shell gradually increased around 2.5 ~ 3.3 Å, while the presence of Bi–S bonds could still be observed. That is to say, when Bi^3+^ is reduced to Bi^0^ through the CO_2_RR process, the residual S regulates the electronic structure of Bi, thereby optimizing the adsorption strength of the intermediate. DFT calculations show that S doping significantly reduces the formation energy barrier of HCOO*, promotes forming formic acid, increases the formation energy barrier of *COOH and H* intermediates, and inhibits the formation of CO and H_2_ byproducts. Also, Chen et al. [92] also used a simple chemical reduction method to prepare Bi catalysts doped with B atoms and realized efficient electrochemical reduction of CO_2_. Notably, the Faraday efficiency of formate can be maintained above 90% over a wide potential range of − 0.6 to − 1.2 V (compared to reversible hydrogen electrodes [RHE]). As shown in Fig. 6d, e, the experimental and calculation results show that for pure Bi, it is obvious that the OCHO* is strongly adsorbed on the surface, which makes it hard to be reduced to formate. Once B is doped and induces the formation of electron-rich Bi, the reduction of OCHO* is remarkably facilitated. (The energy gap of Bi (B) is 0.56 eV while that of pure Bi is 0.78 eV.) The reduction of OCHO* is remarkably facilitated. The reason is that the increased electron density at Bi is able to weaken the bonding strength between metallic active center and more electronegative O, and thereby lower the barrier of the reduction of OCHO*. Furthermore, the formation of COOH* (the intermediate of CO) over Bi(B) is very difficult, which is consistent with the low Faradaic efficiency of CO over the whole potential range. Meanwhile, the formation of H* of Bi(B) is strongly suppressed (1.32 eV of Bi(B) vs. 0.74 eV of Bi), indicating the effective inhibition of HER with B dopant.

**Fig. 7:**
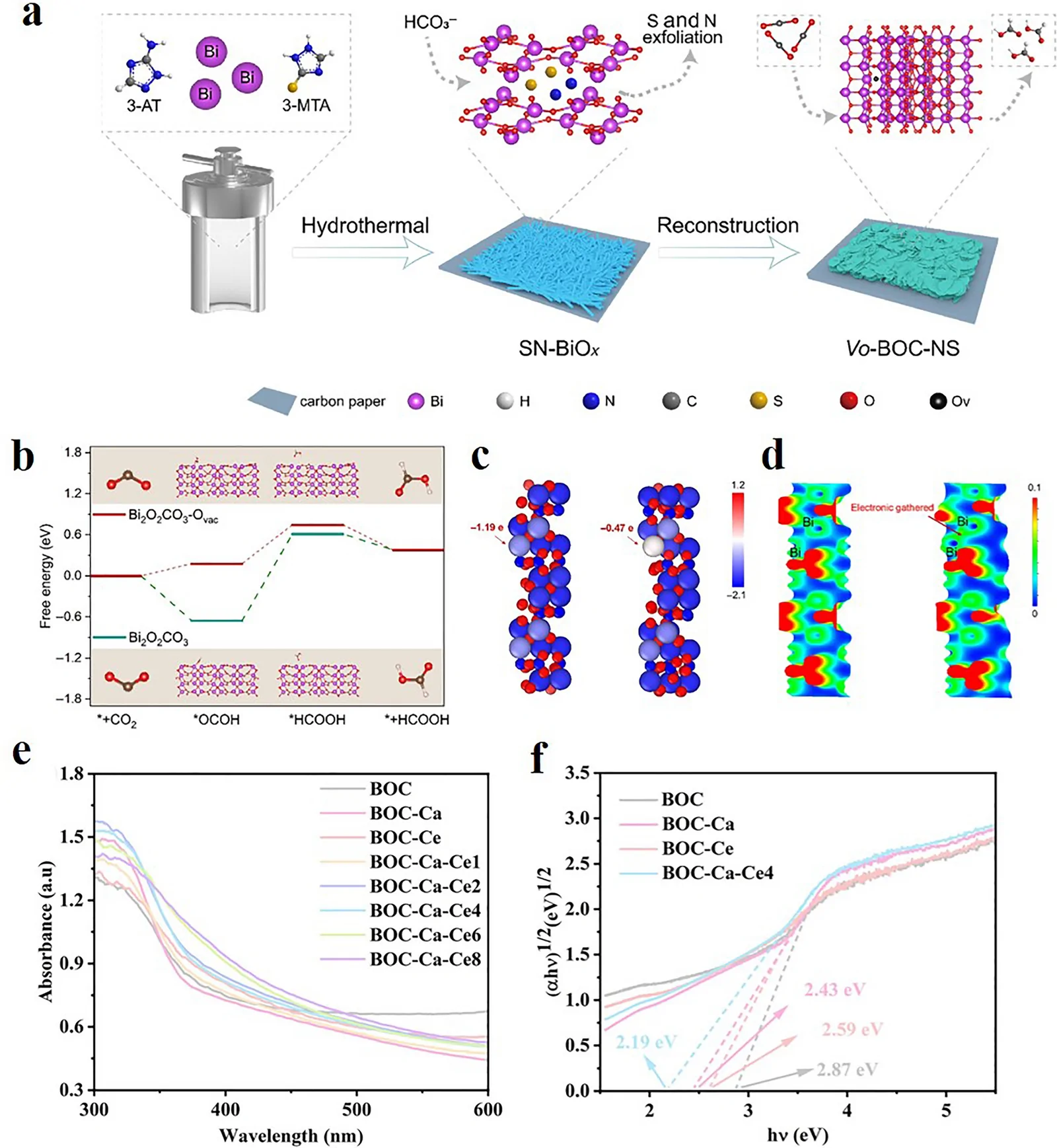
Co-doping strategy optimizes the electronic structure and reaction pathways. **a** Fabrication and reconstruction from Sn–BiO_x_ to Vo-BOC-NS.; **b** Gibbs free energy of key intermediates for CO_2_RR to HCOOH; **c** Bader charge density difference; **d** corresponding 2D electron localization function (ELF) of the left: Bi_2_O_2_CO_3_ and right: Bi_2_O_2_CO_3_ with one oxygen vacancy. Red and blue represent higher and lower levels of electron localization, respectively [93]; © 2022 The Authors. **e** UV–Vis diffuse reflectance spectra and **f** Tauc curves of BOC and BOC-Ca-Ce_x_ (X = 1, 2, 4, 6, 8) materials [95]. © 2025 Elsevier Inc

Atomic doping is an important means to regulate the performance of Bi-based catalysts, while co-doping (that is, introducing two or more heterogeneous atoms simultaneously) can further optimize the electronic structure, surface active sites, and reaction pathways of materials through a synergistic effect. Co-doping can significantly alter the band structure and charge distribution of Bi-based materials through the orbital hybridization of different atoms or the charge compensation effect. Based on this, Zhang et al. [93] proposed ultra-thin BOC nanosheets (Vo-BOC-NS) with abundant Vo reconstructed by co-doped Bi oxides of two nonmetallic elements S and N, as shown in Fig. 7a, which can be used as a durable electrocatalyst for the conversion of CO_2_ to formic acid. The XRD pattern indicates that the gas diffusion electrode (GDE) modified by Sn–BiO_x_ was reconstructed into Vo-BOC-NS after being immersed in 0.5 M KHCO_3_ electrolyte for half an hour. The existence of Vo and the evolution of elemental valence states in Vo-BOC-NS were verified by methods such as Raman spectroscopy, electron electromagnetic resonance, and XPS. As shown in Fig. 7b, experiments and theoretical calculations indicate that during the reconstruction process, the release of N and S elements and the structural transformation of the precursors generate a large number of Vo. The large number of positively charged Vo induces the reconfiguration of electron density. Among them, the Vo, as electron donors, can transfer more electrons to the surrounding Bi metal and form a local alkaline environment around the metal. This increases the electron density around the metal (Fig. 7c, d), thereby reducing the energy barrier for the adsorption and activation of CO_2_ molecules and promoting further reactions. For another achievement, Liu et al. [94] reported a novel sulfur–oxygen co-doped Bi-based catalyst, which is derived from Bi sulfide (Bi_2_S_3_) and is used for the selective electrochemical reduction of CO_2_ to formate. Characterization and electrochemical results show that sulfur atoms and oxygen atoms have high electronegativity. The co-doping of the two improves the surface electronic structure of the material, increasing the electron density around Bi atoms, which is conducive to the transfer of electrons from the catalyst to CO_2_, enabling the faster formation of intermediates and thereby enhancing catalytic performance. Sun et al. [95] proposed a Ca^2+^/Ce^3+^ bimetallic ion co-doping strategy to collaboratively optimize the carrier kinetics and surface reactivity of BOC. A novel BOC-Ca-Ce_X_ (X represents the molar percentage of Ce^3+^ to Bi^3+^, and the values of X are 1%, 2%, 4%, 6%, and 8%, respectively) catalyst was constructed through controllable doping engineering. The system reveals the synergistic enhancement mechanism of bimetallic sites on the gradient regulation of banded structures and the generation of reactive oxygen species. Figure 7e shows the UV–Vis diffuse reflectance spectra (UV–Vis DRS) of BOC and its different doped samples. The UV–Vis DRS analysis shows that the absorption edge of the undoped BOC sample is located near 420 nm, and the corresponding band gap is 2.87 eV. After single doping modification, the absorption edges of BOC-Ca and BOC-Ce samples were redshifted. It is worth noting that in the Ca/Ce co-doped system (BOC-Ca-Ce_X_), as the doping ratio increases, the absorption edge shows a regular redshift trend. Among them, the BOC-Ca-Ce_4_ sample exhibits significant light absorption characteristics, and its absorption intensity in the 300–460 nm band is significantly better than that of pure BOC. Although the absorption intensity decreases slightly in the band above 460 nm, it still maintains good light response ability. The comprehensive analysis shows that the BOC-Ca-Ce_4_ sample has excellent visible light capture ability. Through Tauc curve calculation (Fig. 7f), the band gap of undoped BOC is 2.87 eV, which is consistent with the band gap of pure BOC reported in the literature [96]. In the single-doped samples, the band gaps of BOC-Ca and BOC-Ce were reduced to 2.43 and 2.59 eV, respectively, indicating that the single doping of Ca and Ce can reduce the band gap by introducing impurity energy levels or lattice distortion. It is notable that the band gap of Ca/Ce co-doped samples is further reduced. The band gap of BOC-Ca-Ce_4_ is 2.19 eV, which is significantly lower than that of single-doped and low-proportion co-doped samples. With the increase in the co-doping concentration, the absorption edge of the sample shows a systematic redshift trend. The results show that the band gap values of BOC-Ca-Ce, BOC-Ca-Ce_6_ and BOC-Ca-Ce_8_ are significantly smaller than those of the single-doped system, while the band gap value of the BOC-Ca-Ce_1_ sample is smaller than that of BOC and BOC-Ca, but still slightly higher than that of the BOC-Ce sample. This phenomenon can be attributed to the synergistic effect of Ca^2+^ and Ce^3+^/Ce^4+^. The introduction of Ca may cause lattice expansion by replacing the Bi^3+^ position, while the valence state change of Ce can regulate the electronic structure, form intermediate energy levels, and reduce electron–hole recombination. The continuous reduction of the band gap is consistent with the redshift trend of the light absorption edge, indicating that the co-doping strategy effectively optimizes the band structure of BOC, thereby enhancing its visible light-driven photocatalytic activity. Through the coupling mechanism of band engineering and space charge separation, this work broke through the efficiency limitations of traditional single-component doping, promoting the design and development of bifunctional photocatalysts with wide spectral response and high stability.

The doping engineering strategy effectively regulates the electronic structure, band position, and intermediate adsorption behavior of Bi-based catalysts by introducing heterogeneous atoms (such as metals Ag, Ru, Co and nonmetals S, B) or co-doping (such as S/N, Ca/Ce), significantly enhancing the activity and selectivity of the CO_2_RR. Metal doping optimizes the carrier separation efficiency through orbital hybridization or charge transfer channels, reducing the energy barrier for CO_2_ activation and intermediate conversion. Nonmetallic doping inhibits the hydrogen evolution reaction by adjusting the surface electron density and enhances the stability of the *OCHO intermediate. The co-doping strategy further couples with lattice distortion through band gradient regulation, broadens the light absorption range, and improves the charge separation efficiency, breaking through the limitations of single doping. However, the atomic-level precise regulation of doping concentration and distribution still poses challenges. Excessive doping may cause an intensification of carrier recombination (such as Ru cluster doping leading to shielding of active sites). The dynamic stability is insufficient. Some doped atoms undergo valence reduction or dissolution during the reaction, resulting in irreversible inactivation of the active sites. Selective enhancement often comes at the expense of multielectron reaction pathways. For example, while S doping inhibits the generation of CO, it may hinder the extension of C–C coupling to high-value-added products. It is worth mentioning at this time is that strategies such as defect engineering and crystal plane engineering can be combined with doping engineering to synergically enhance the performance of Bi-based catalysts, and appropriate multiple in situ characterization techniques can be applied to deeply reveal the dynamic evolution mechanism of the doping sites.

### Organic Framework Engineering

#### Bi-Based/COFs Catalysts

**Fig. 8:**
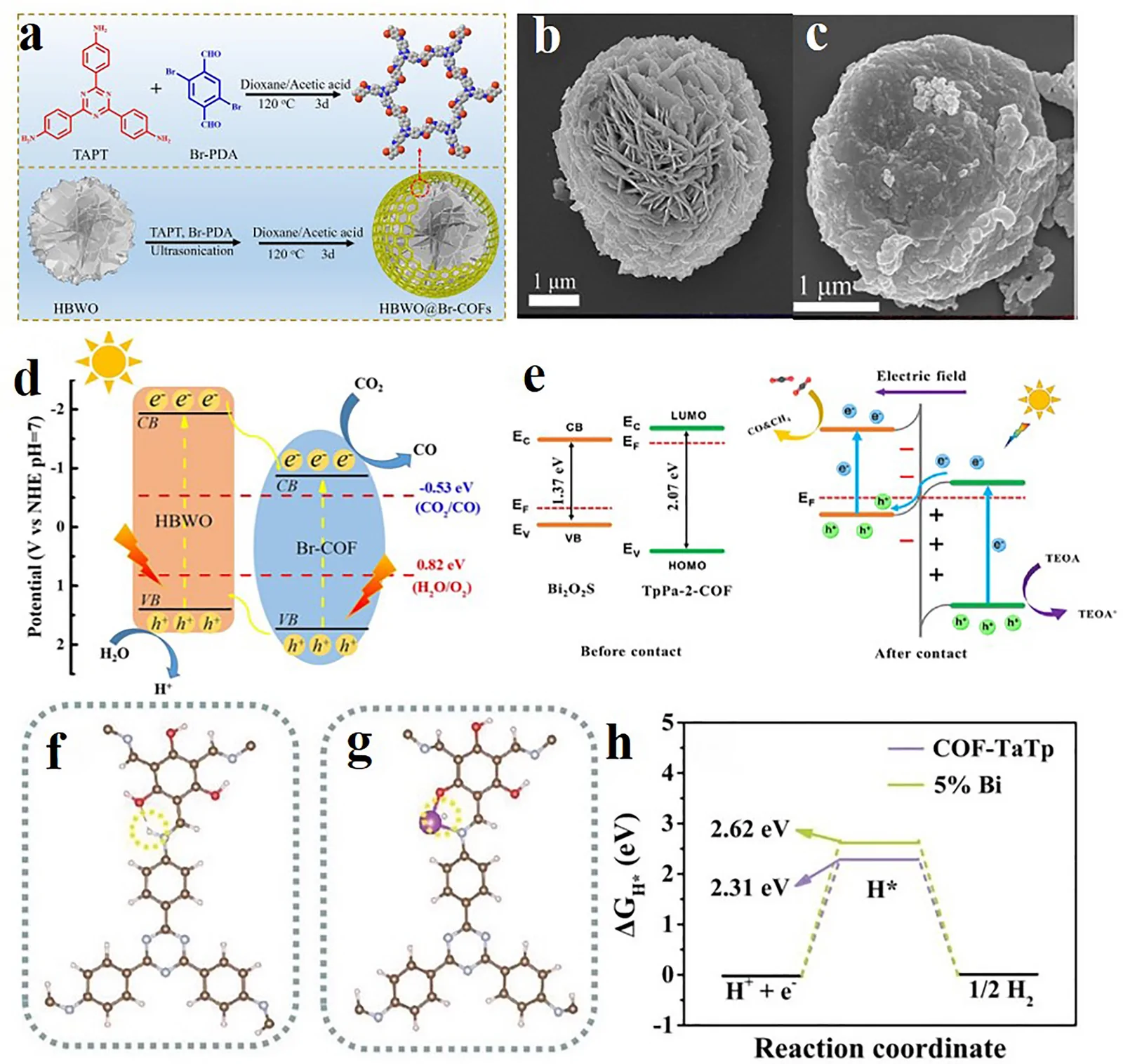
Hierarchical structure design and mechanism of photoelectrocatalytic CO_2_RR with COF hybrid materials. **a** Schematic illustration of the preparation of HBWO@Br-COFs hybrid materials. SEM images of **b** HBWO and **c** HBWO@Br-COF-2; **d** electron transfer pathway and CO_2_ reduction mechanism [100]; © 2023 Elsevier B.V.** e** Enhanced photocatalytic CO_2_RR mechanism of Bi_2_O_2_S@TpPa-2-COF heterojunction [101]. © 2023 Elsevier B.V. Top view of optimized single hydrogen adsorption configuration of **f** COF-TaTp, and **g** 5% Bi/COF-TaTp. **h** Free energy diagram for hydrogen evolution of COF-TaTp and 5% Bi/COF-TaTp [102]. © 2022 Wiley‐VCH GmbH

When the regulation of electronic structure at the atomic scale tends to be perfected, researchers begin to turn their attention to mesoscopic structural engineering. By combining precisely designed covalent organic frameworks (COFs) with Bi-based catalysts, a catalytic microenvironment with spatial confinement effects can be constructed while maintaining the advantages of electronic regulation, achieving cross-scale collaborative optimization from atomic arrangement to nanostructures. COFs [97] are a new type of porous material, formed by organic molecules connected through covalent bonds, which have various architectural structures such as one-dimensional (1D), two-dimensional (2D), and three-dimensional (3D), and exhibit high porosity, large specific surface area, and excellent stability. By choosing organic monomers with different symmetries (such as benzene rings, triazines, and porphyrins) and connection methods (imine bonds, borate ester bonds, etc.), the pore size and topological structure can be customized. It has a high porosity, specific surface area, and stability. The pore structure of COFs is clear, and it is easy to combine with Bi-based nanomaterials. COFs and Bi-based nanomaterials can be combined into a hybrid material to achieve a synergistic effect, thereby improving their catalytic performance [98, 99]. Wang et al. [100] successfully synthesized an HBWO@Br-COFs hybrid material with a clear core–shell structure by the one-pot solvent method using nano-Bi_2_WO_6_ as the core and triazine imine-functionalized Br-COF as the shell, as shown in Fig. 8a. The pure HBWO group was divided into nanoflowers assembled by nanosheets with an average diameter of 2.5 μm (Fig. 8b). After the Schiff base reaction, Br-COF is uniformly coated on the surface of HBWO (Fig. 8c), showing core–shell characteristics. FT-IR and XPS analyses confirmed that W–O–C covalent bonds were formed at the interface, enhancing the electron transfer efficiency. Meanwhile, HBWO (positively charged, zeta potential + 4.00 mV) and Br-COF (negatively charged, -10.6 mV) also form close contact through electrostatic attraction, promoting the stable construction of heterojunctions. Figure 8d shows the possible CO_2_ emission reduction mechanisms. Under visible light irradiation, HBWO and Br-COF surfaces continuously produce a large number of photogenerated electrons, which transfer from the valence band (VB) to the corresponding conduction band (CB), while the photogenerated holes remain in the original VB. HBWO (-1.93 eV) and Br-COF (-0.87 eV) have more negative CB than CO_2_/CO (-0.53 eV), while HBWO is more negative CB, so photoelectrons are transferred from HBWO Br-COF via C-O covalent bonds. These results show that the interfacial heterojunction is beneficial to the reduction of CO_2_ to CO. On the contrary, at the heterojunction, the photogenic holes of Br-COF transform from VB to HBWO and gather together to oxidize H_2_O. The continuous migration of light-generated electrons and holes promotes the separation of electron–hole pairs, thus improving the photocatalytic CO_2_ reduction effect. Wu et al. [101] successfully constructed a TPA-2-COF/Bi_2_O_2_S nanosheet p–n heterojunction photocatalyst by an in situ growth strategy, and its interface achieved strong coupling by forming covalent bonds through C–S bonds and N–O bonds. As shown in Fig. 8e, the mechanism by which the heterojunction enhances photocatalytic CO_2_ reduction can be systematically described as follows: before the heterostructure is constructed, the Fermi level of TPA-2-COF is significantly higher than that of Bi_2_O_2_S. When the n-type semiconductor TPA-2-COF and p-type Bi_2_O_2_S form interfacial contact, electrons spontaneously migrate from TPA-2-COF to Bi_2_O_2_S until the Fermi levels of both reach equilibrium. This charge redistribution process forms an internal electric field from TPA-2-COF to Bi_2_O_2_S at the interface, accompanied by band bending, and finally forms a space charge region in the interface. Under visible light excitation, photogenerated electrons are excited from the VB of TPA-2-COF and Bi_2_O_2_S to the CB. To achieve the purpose of electron–hole segregation, a large number of electrons are gathered in the VB of TPA-2-COF, and under the action of the internal electric field, these electrons are moved to the VB of Bi_2_O_2_S and recombined with the Bi_2_O_2_S hole generated by the photoexcitation. CO_2_ is converted into CO and CH_4_ by electrons enriched in the Bi_2_O_2_S guide band, and the holes accumulated in the TPA-2-COF VB are devoured by the sacrifice agent TEOA, thus realizing the effective separation of photogenerated carriers and greatly improving the efficiency of photocatalytic CO_2_RR. Covalent bond connection enables the composite material to maintain high activity after multiple cycles, and the stability has also been significantly improved. The CO yield of covalent bond heterojunctions (19.5 μmol g^−1^ h^−1^) was 3.96 times and 66.8 times higher than that of pure Bi_2_O_2_S and TpPa-2-COF, respectively, and the CH_4_ yield reached 6.2 μmol g^−1^ h^−1^. In addition, Yu et al. [102] designed a novel Bi-modified imine linkage COF-TaTp (Bi/COF-TaTp) catalyst through N–Bi–O coordination. Theoretical calculations (Fig. 8f-h) show the adsorption geometry of ΔG_H*_ photocatalyst and H. COF-TaTp modified with the appropriate number of Bi nanoparticles significantly promotes the separation and migration of photoinduced electron–hole pairs, effectively inhibits HER competition, and thus greatly improves the reaction rate and yield of target products.

**Fig. 9:**
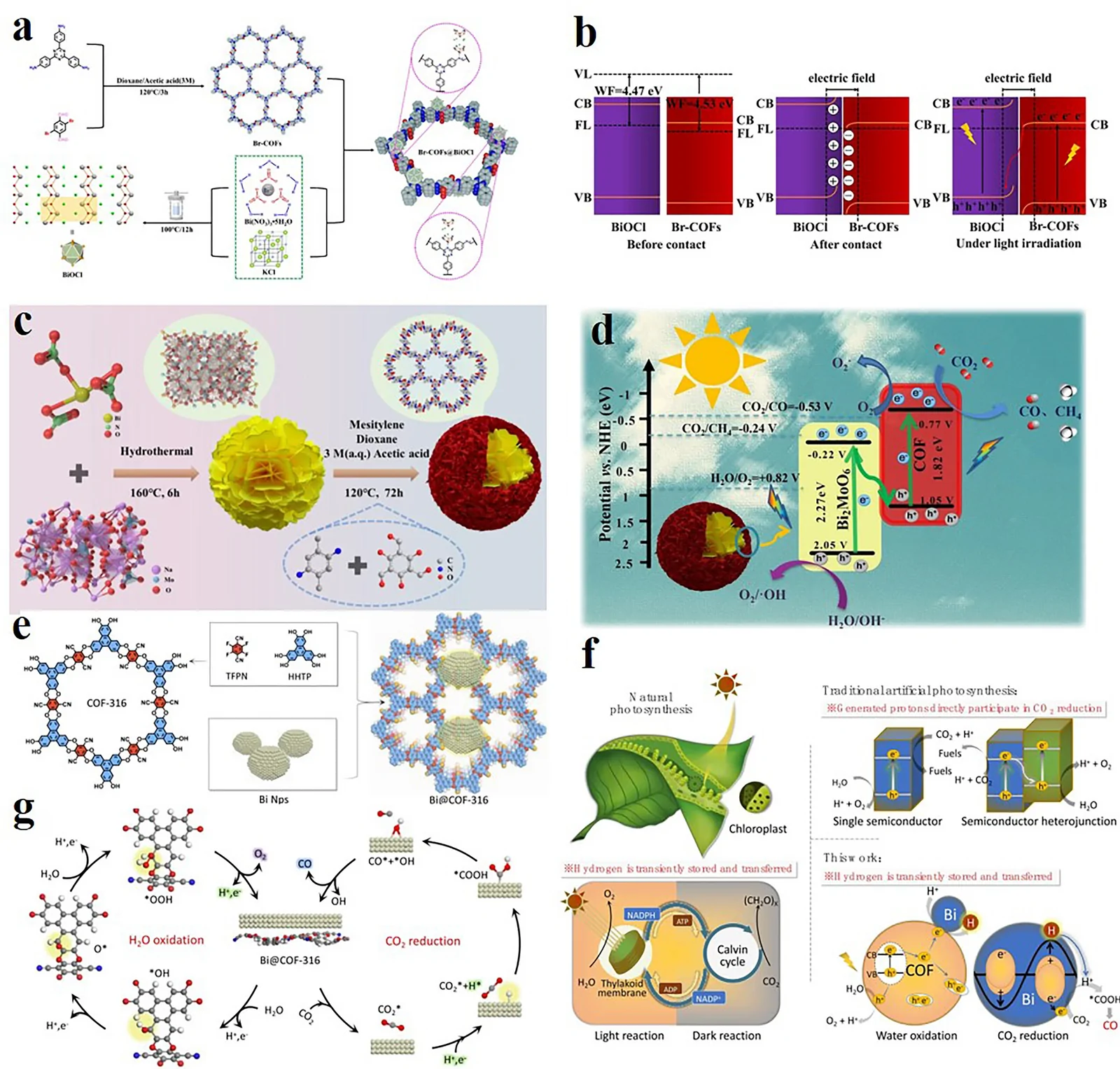
Multidimensional structural engineering of Bi-based COF heterojunctions and the cross-scale charge transport mechanism jointly regulate CO_2_RR. **a** Schematic illustration for the preparation of Br-COFs@BiOCl composites; **b** schematic illustration of the relative band positions and charge transfer process before contact, after contact and under light irradiation [103]; © 2025 Elsevier B.V. **c** Preparation process for the core–shell Bi_2_MoO_6_@COF composites; **d** proposed mechanism of photocatalytic CO_2_ reduction for Z-scheme Bi_2_MoO_6_@COF heterojunction [104]; © 2025 Elsevier B.V.** e** Schematic diagram of synthesis of Bi@COF-316; **f** schematic depiction of natural and artificial photosynthesis; **g** schematic diagram of the photocatalytic CO_2_ reduction and H_2_O oxidation mechanism. [105]. © 2025 Elsevier B.V

For another achievement, Wang et al. [103] developed triazinyl bromo-substituted COFs (Br-COFs) to synthesize the Br-COFs@BiOCl photocatalyst by in situ anchoring BiOCl to Br-COFs (Fig. 9a). Covalent N-Bi bonds are formed at the interface, creating a Z-type heterojunction. The formation of covalent N-Bi bonds not only enhances the interface stability but also provides an efficient electron transport channel, significantly improving the separation efficiency of photogenerated carriers. Based on the photogenerated carrier migration in Z-type heterojunctions, a potential catalytic process and photocatalytic mechanism were proposed, as shown in Fig. 9b. The excited state electrons generated in Br-COFs transfer along the Z-scheme path to the BiOCl, where they recombine with the holes in the valence band on the interface. This photoexcitation event leads to the generation of more electrons, which then migrate to the CB of the BiOCl, resulting in the accumulation of electrons in this region. These electrons enter the surface of the catalyst, promoting the conversion of adsorbed CO_2_ molecules into CO and CH_4_. Meanwhile, the holes generated by light remain within the VB of Br-COFs, promoting the oxidation of H_2_O. The continuous migration of these photogenerated electrons and holes significantly enhances the separation of electron–hole pairs, thereby improving the photocatalytic CO_2_RR. The conversion rate of CO_2_ to CO was 27.4 μmol g^−1^ h^−1^, with a selectivity close to 100%, which was superior to pure BiOCl and Br-COFs. Wu et al. [104] combined Bi_2_MoO_6_ nanospheres with the organic covalent framework TpPa-2-COF to construct a core–shell Bi_2_MoO_6_@COF photocatalyst, which facilitated the photocatalytic CO_2_RR in water vapor, as shown in Fig. 9c. The Z-type heterojunction formed by N–O covalent bonds between Bi_2_MoO_6_ and TpPa-2-COF promotes interfacial charge transfer and improves the separation efficiency of electrons and holes. SEM and TEM images showed that TpPa-2-COF tightly wrapped Bi_2_MoO_6_ nanospheres in a “cobweb-like” structure, which increased the interface contact area and provided more active sites. In addition, the photocatalytic CO_2_RR mechanism of the prepared Z-type Bi_2_MoO_6_@COF heterojunction was proposed and shown in Fig. 9d. During the light collection process, there are two active visible light absorption centers, namely Bi_2_MoO_6_ nanoflowers and organic covalent frameworks. The charge transfer process mainly relies on photoexcitation and the built-in electric field, that is, the photogenerated electrons transfer from the conduction band of Bi_2_MoO_6_ to the valence band of TpPa-2-COF. The REDOX ability of the holes in TpPa-2-COF and the electrons in Bi_2_MoO_6_ is relatively weak and they are sacrificed during the reaction process, while the electrons in TpPa-2-COF and the holes in Bi_2_MoO_6_ have strong REDOX ability. They jointly regulate the charge potential and kinetics to meet the dual functional requirements of aerobic photocatalytic oxidation and anaerobic photocatalytic reduction. The mechanism of surface photocatalytic reactions depends on the reaction environment. Under aerobic conditions, the holes in the Bi_2_MoO_6_ valence band can activate H_2_O, promote the generation of ·OH, which is conducive to the oxidation of malachite green (MG) or the hydrogenation of CO_2_ through water cracking. Electron-initiated reactions are more complex. The electrons in TpPa-2-COF can activate O_2_ to generate ·O_2_^−^, which works in synergy with ·OH to effectively oxidize MG. Under anaerobic conditions, the ·O_2_^−^ produced by electron excitation is inhibited, resulting in a significant decrease in the removal efficiency of MG. On the contrary, under such conditions, the photogenerated electrons in the TpPa-2-COF valence band will dominate the reduction of CO_2_ to CO and CH_4_. Natural photosynthesis occurs through continuous water splitting and carbon dioxide reduction reactions, both of which are ingeniously linked to the short-term storage of hydrogen. The optimized Bi_2_MoO_6_@COF photocatalyst demonstrated excellent catalytic performance in the CO_2_RR, with CO_2_ reduction rates of 12.71 μmol g^−1^ h^−1^ and CH_4_ reduction rates of 5.5 μmol g^−1^ h^−1^, which were 2.73 times and 1.42 times that of Bi_2_MoO_6_, respectively. This is mainly attributed to the interface synergy effect. However, the performance of the composite materials prepared by mechanical mixing is significantly lower than that of the in situ grown samples. This is mainly because chemical bonds and physical contact jointly regulate light absorption, charge-charge separation and surface reaction kinetics, thereby enhancing the overall photocatalytic performance. Zhang’s team [105] used plasma Bi as the hydrogen storage medium and combined it with COFs to simulate the key process of hydrogen storage in natural photosynthesis (Fig. 9e, f). The COF-316 exhibits strong absorption at 380–570 nm, and the calculated band gap is 2.42 eV. When Bi NPs is incorporated, the light absorption range of Bi@COF-316 is significantly expanded up to 800 nm. Based on experimental and theoretical calculations, the internal mechanism of Bi@COF-316 photocatalytic CO_2_RR was proposed, as shown in Fig. 9g: under light irradiation, COF-316 is excited and photoelectrons are transferred from VB to CB. Then the photogenerated electrons in the CB of COF-316 rapidly migrate to the Fermi level of Bi NPs in the hybrid material, facilitating the separation of photogenerated carriers in Bi@COF-316. The water splitting caused by photoelectron–hole pairs in COF-316 simultaneously generates O_2_ and hydrogen storage Bi (Bi–H). Here, it should be pointed out that the Fermi energy level of Bi NPs is approximately –0.17 V (vs NHE, pH 7), which is not feasible for CO_2_-to-CO reduction (−0.52 V vs NHE, pH 7). Therefore, the CO_2_-to-CO reduction is completely dependent on the high-energy thermoelectrons excited by the SPR effect light of Bi NPs. Unlike the photoexcited charges in semiconductors, the lifetime of the thermal electrons produced by SPR is much shorter. Therefore, it is particularly important to improve the separation efficiency of thermal charges in plasma metals. On the basis of the above considerations, the role of stored H atoms in the CO_2_ reduction of Bi–H@COF-316 is discussed. Firstly, under the action of light, thermal electron–hole pairs are generated on the irradiated Bi–H NPs through plasma excitation. Hydrogen atoms stored in Bi–H@COF-316 consume plasma hot holes (H + h → H). In one operation, active H and e^+++−^ were successively produced at Bi–H@COF-316, and these active substances reacted with the active CO_2_ molecule at the active site. According to this mechanism, by positioning H in the Bi component, both the recombination of photoexcited carriers can be inhibited and the giving of electron–proton pairs can be enhanced. This is why Bi@COF-316 has excellent CO_2_ reduction performance through the proton-assisted electron transfer pathway (CO_2_ + 2H + 2e^+−^ → CO + H_2_O). In addition, the interdependent nature of H and e^+−^ prevents the excessive accumulation of e^−^ or H during the photocatalytic process, which also helps to enhance the activity of Bi@COF-316. This strategy achieved a high CO production efficiency of 185.42 μmol g^−1^ h^−1^ without the use of photosensitizers and sacrifice reagents.

The COF composite strategy significantly enhances the CO_2_RR performance of Bi-based catalysts by constructing spatial confinement effects and covalent bond interfaces (such as N–Bi–O coordination and Z-type heterojunctions). The high specific surface area and customized channels of COFs enhance CO_2_ adsorption. Meanwhile, the covalent bond interface optimizes the carrier separation efficiency. The coupling of pore confinement of COFs with the electronic regulation of Bi-based active sites inhibits photogenerated electron–hole recombination. However, the synthesis time of COFs is generally long, and the interface charge transport mechanism depends on band matching (for example, Bi@COF-316 depends on plasma thermal electrons, whose short lifetime limits the reaction kinetics). Meanwhile, although the hydrophobic property of COFs inhibits the hydrogen evolution side reactions (such as the HER inhibition of Bi/COF-TaTp), it may weaken the multielectron reduction path of CO_2_, and the excessive pursuit of interfacial charge separation may sacrifice the wide potential adaptability of the catalyst. Therefore, it is necessary to balance the structural stability of COFs and the design of the dynamic reaction microenvironment to achieve the unity of high activity and high selectivity.

#### Bi-Based/MOFs Catalysts

**Fig. 10:**
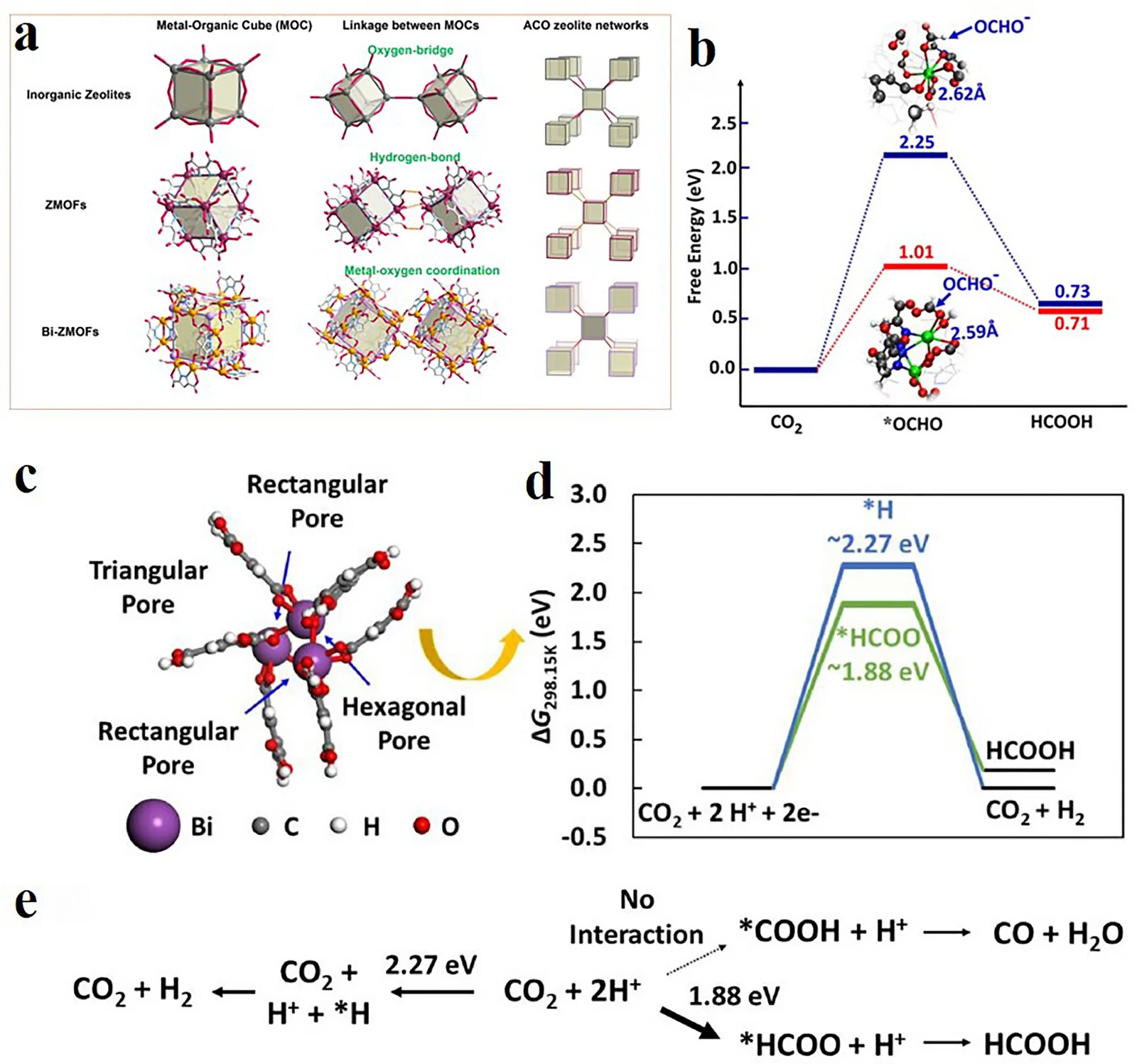
Structural evolution and catalytic mechanisms of Bi-MOFs. **a** Schematic illustration of metal–organic cube, linkage, and ACO topology from inorganic zeolites to ZMOFs, and Bi-ZMOFs; **b** free energy diagrams for HCOOH formation on the Bi of PZH-1 (red) and PZH-2 (blue) system, respectively. [110]; © 2023 Wiley‐VCH GmbH. **c** Simulated cluster representing Bi-MOF catalytic sites. Blue arrows show the binding sites of adsorbates; **d** free energy diagram of HER and HCOOH formation. The ~ energy value indicates the average energy of the three binding sites; **e** suggested reaction pathway. Each arrow indicates a reaction with an addition of an electron. *COOH, an intermediate for CO formation, does not bind [111]. © 2020 Elsevier B.V

In addition to the organic porous framework constructed by covalent bonds, the metal–organic hybrid system provides another path for the structural regulation of Bi-based catalysts. Compared with conjugated organic polymers, metal–organic frameworks (MOFs), through the metal–ligand coordination network, further integrates the intrinsic catalytic activity of metal nodes on the basis of retaining the advantages of high specific surface area and customizable pores, and forms functional complementarity with the pure organic confinement effect of COFs, jointly expanding the collaborative design dimension of porous materials and Bi-based catalysts. MOFs [106] are another kind of porous material formed by metal ions or metal clusters connected with organic ligands through coordination bonds. Their highly adjustable structure, large specific surface area, and unique pore properties make them promising for a wide range of applications. Bi-based organic scaffolds (Bi-MOFs) are highly efficient catalysts for CO_2_RR. Through metal–organic co-catalysis, structural programmability and multidimensional mass transfer channels, MOF has solved the bottlenecks of Bi-based materials in photoelectrocatalysis, such as insufficient active sites, severe charge recombination and poor stability. Meanwhile, it has an extremely high specific surface area, which can enhance the adsorption capacity of CO_2_, can accelerate the diffusion of CO_2_, has strong stability, and is resistant to acids and alkalis and oxidation. It can better protect Bi-based materials. However, traditional conjugated polymers such as polyaniline [107] and polythiophene [108], due to the lack of metal active centers and ordered structures, have a narrow light absorption range, limited band regulation, and are prone to degradation, making it difficult to achieve the same level of performance breakthroughs. Liu et al. [109] prepared four new Bi-MOFs with different shapes and sizes through hydrothermal reaction. The experimental results show that the topological structure of the precursor significantly affects the microstructure evolution of the derived Bi-based catalyst, especially its interface contact with the carbon paper substrate. Among the four groups of Bi-MOFs precursor derivatives with different morphologies, the Bi-MOF-NC-derived Bi catalyst showed the best CO_2_RR performance. Through the combination of Gibbs free energy calculation and differential charge density analysis, the CO_2_RR reaction path dominated by Bi (012) crystal plane was systematically clarified: The CO path (*COOH formation energy ΔG =  + 1.08 eV) and H_2_ path (*H formation energy ΔG =  + 1.05 eV) are limited to a high activation barrier, while the HCOOH path has a rate-determining step (*CO_2_ → HCOO*) with an energy barrier of only 0.67 eV. This electronic structural feature effectively reduces the kinetic barrier of CO_2_ activation by enhancing the antibonding orbital occupation of the C=O bond. The activation energy required for HCOOH formation is significantly lower than that for CO or H_2_ formation, which is consistent with the high selectivity of Bi-MOF-NC for formic acid formation. To further improve the activity and stability of Bi-based MOFs catalysts, Jiang et al. [110] successfully constructed a novel Bi-based zeolite imidazolate skeleton material with ACO topological lattice (Bi-ZMOF, named PZH-1) by using a ligand-oriented assembly strategy, and its unique Bi-based cage structure was stabilized by strong coordination bonds, as shown in Fig. 10a. This kind of material showed excellent formic acid selectivity in the electrocatalytic CO_2_RR. From the in situ surface-enhanced infrared absorption spectroscopy and DFT calculations shown in Fig. 10b, the regulatory mechanism of Bi–N coordination on catalytic performance was revealed: the electrons transfer from the coordinated N atoms to the Bi center. This electron redistribution significantly enhanced the adsorption stability of the key intermediate *OCHO, and reduced the activation energy of CO_2_. This discovery clarified the precise regulation of the coordination microenvironment of the metal active center on the electrocatalytic CO_2_RR path from the atomic scale and provided a new idea for the rational design of efficient CO_2_RR catalysts. Also, Li et al. [111] prepared 2D porous Bi-MOF catalysts with adjustable crystallinity and coordination environment by manipulating the reaction temperature and the type of organic linker. According to the calculation of DFT, the thermochemical processes of CO, HCOOH and H_2_ formation pathways were considered, as shown in Figs. 10c ~ 10e. The three binding sites were found to exhibit similar energetics (Fig. 9d). Notably, *COOH is an intermediate that forms CO, but *COOH does not bind at the binding site (Fig. 10e). This suggests that *COOH is not formed from CO_2_ and H, explaining the low selectivity toward deoxidized substances such as CO, CH^+^_4_, and CH_3_OH. On the other hand, the reaction binding energy of the intermediate *HCOO forming HCOOH is 1.88 eV, which meets the experimental overpotential required for observing the activity. The calculation shows that HER is inhibited on Bi-MOF because the formation energy of *H is 2.27 eV, which is 0.39 eV larger than that of *HCOO. In conclusion, these results provide a theoretical basis for the activity, selectivity, and overpotential trend of Bi-MOF catalysts.

**Fig. 11:**
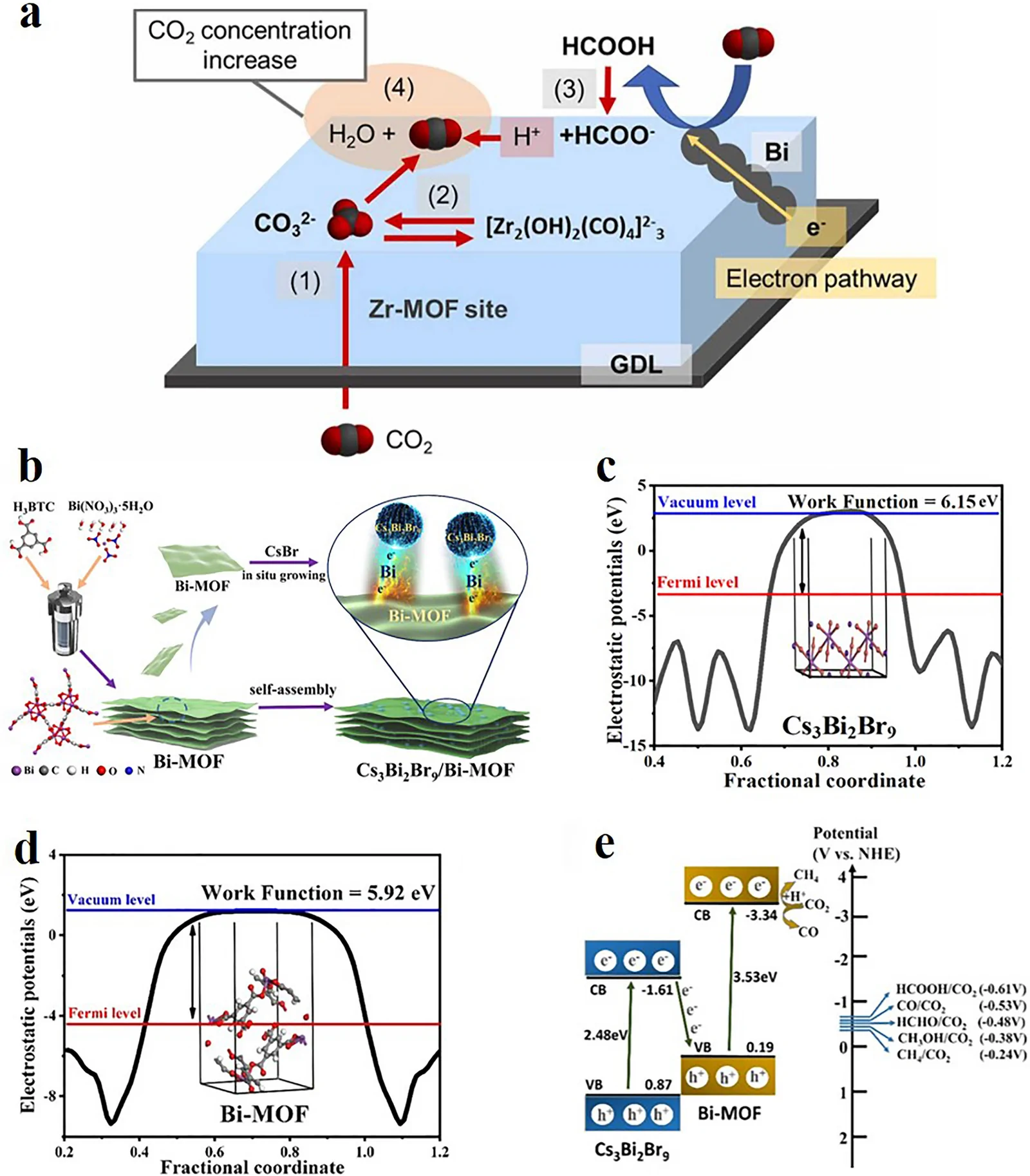
Interfacial engineering and reaction mechanisms in Bi-MOF hybrid systems. **a** Proposed mechanism for the improved CO_2_ reduction activity with Bi/UiO-66-Zr-MOF structure. In the schematic, (1) ~ (4) correspond to the chemical reactions of Zr_6_O_4_(OH)_4_(BDC)_6_ + 18HCO_3_^−^ → 3[Zr_2_(OH)_2_(CO_3_)_4_]^2−^ + 8 H_2_O + 6CO_2_ + 6BDC^2−^, CO_2_ + OH^−^ ↔ HCO_3_^−^, HCOOH ↔ H^+^  + HCOO^−^ and 2CO_3_^2−^ + 4 H^+^  → 2CO_2_ + 2H_2_O, respectively [112]; © 2023 Elsevier B.V. **b** Schematic illustration of the Cs_3_Bi_2_Br_9_/Bi-MOF composite. Electrostatic potential diagrams for **c** Cs_3_Bi_2_Br_9_ and **d** Bi-MOF; **e** diagrams of the relative band edge positions of Cs_3_Bi_2_Br_9_ and Bi-MOF [113]. Copyright © 2023, American Chemical Society

The synergistic effect of Bi-based catalysts and MOF materials can significantly enhance the catalytic CO_2_RR performance. Takaoka et al. [112] proposed a novel strategy of combining UiO-66 MOF structure with Bi electrocatalyst for highly active CO_2_RR with selective HCOOH production. The synthesized Bi/UiO-66 catalyst demonstrates superior CO_2_ reduction properties, 4.6 times higher current density at 0.7 V than bare Bi without UiO-66 despite of low electrochemical surface area. Furthermore, CO_2_ can be captured in the form of carbonate at the Zr-MOF site, as shown in Fig. 11a. When CO_2_ gas flows through the surface between Bi/UiO-66 and the KOH electrolyte, the HCO_3_^−^ generated by the reaction can react with UiO-66 to release [Zr_2_(OH)_2_(CO_3_)_4_]^2−^. This process can be seen as the CO_2_ capture process of MOF structural transformation. At the same time, HCOOH generated on the surface of the Bi catalyst can provide protons through ionization near the catalyst surface, which can release carbonate ions from the Zr site to the Bi catalyst side in the form of CO_2_, and can be used directly for CO_2_RR. Carbonate species captured form of CO_2_ at the Zr-MOF site contribute to the high CO_2_ conversion rate. This work reveals the feasibility of Zr-MOF as a supporting material to achieve efficient CO_2_ reduction. Ding et al. [113] successfully synthesized a new bifunctional Cs_3_Bi_2_Br_9_/Bi-MOF artificial photoreaction structure, as shown in Fig. 11b. Under 300 W Xe lamp irradiation, the prepared Cs_3_Bi_2_Br_9_/Bi-MOF materials captured CO_2_ and performed photoelectric conversion. Based on experimental data and theoretical calculation, the catalytic mechanism can be revealed (Fig. 11c, d): When Cs_3_Bi_2_Br_9_ and Bi-MOF come into contact with each other, the electrons in the Bi-MOF transfer to the Cs_3_Bi_2_Br_9_/Bi-MOF interface until the Fermi level equilibrium. At this point, the Bi-MOF band bends upward and loses electrons. At the same time, due to electron accumulation, the negatively charged interface of Cs_3_Bi_2_Br_9_ shows a downward bending trend. Therefore, under light irradiation, the electrons in CB of Cs_3_Bi_2_Br_9_ are transferred to VB of Bi-MOF. Cs_3_Bi_2_Br_9_ and Bi-MOF can be arranged into a typical direct S-band structure shown in Fig. 11e. In the S-type heterostructure between Cs_3_Bi_2_Br_9_ and Bi-MOF, Cs_3_Bi_2_Br_9_ quantum dots develop in situ on the surface of Bi-MOF nanosheets through Bi atom sharing, which further improves the dispersion of Cs_3_Bi_2_Br_9_ quantum dots on Bi-MOF nanosheets. The active sites for CO_2_ adsorption were also increased. Furthermore, the coulomb electrostatic repulsion is significantly reduced by sharing the same atom, which can accelerate the separation between the photogenerated electrons and the holes, and reduce the activation energy of the carrier transport during the photocatalysis process significantly. The stability of Cs_3_Bi_2_Br_9_/Bi-MOF system is greatly improved by the co-oxidation resistance of Bi and the water solubility of quantum dots. Thus, the combination of Bi-MOF constructed by sharing Bi species with Cs_3_Bi_2_Br_9_ quantum dots can establish electron shuttle paths, improve charge separation efficiency, and strengthen bond connections between them. Compared with conventional heterojunction materials, this improved Bi-based/Bi-MOF structure contributes to enhanced structural stability and excellent CO_2_ capture and conversion activity in gas–solid systems.

**Fig. 12:**
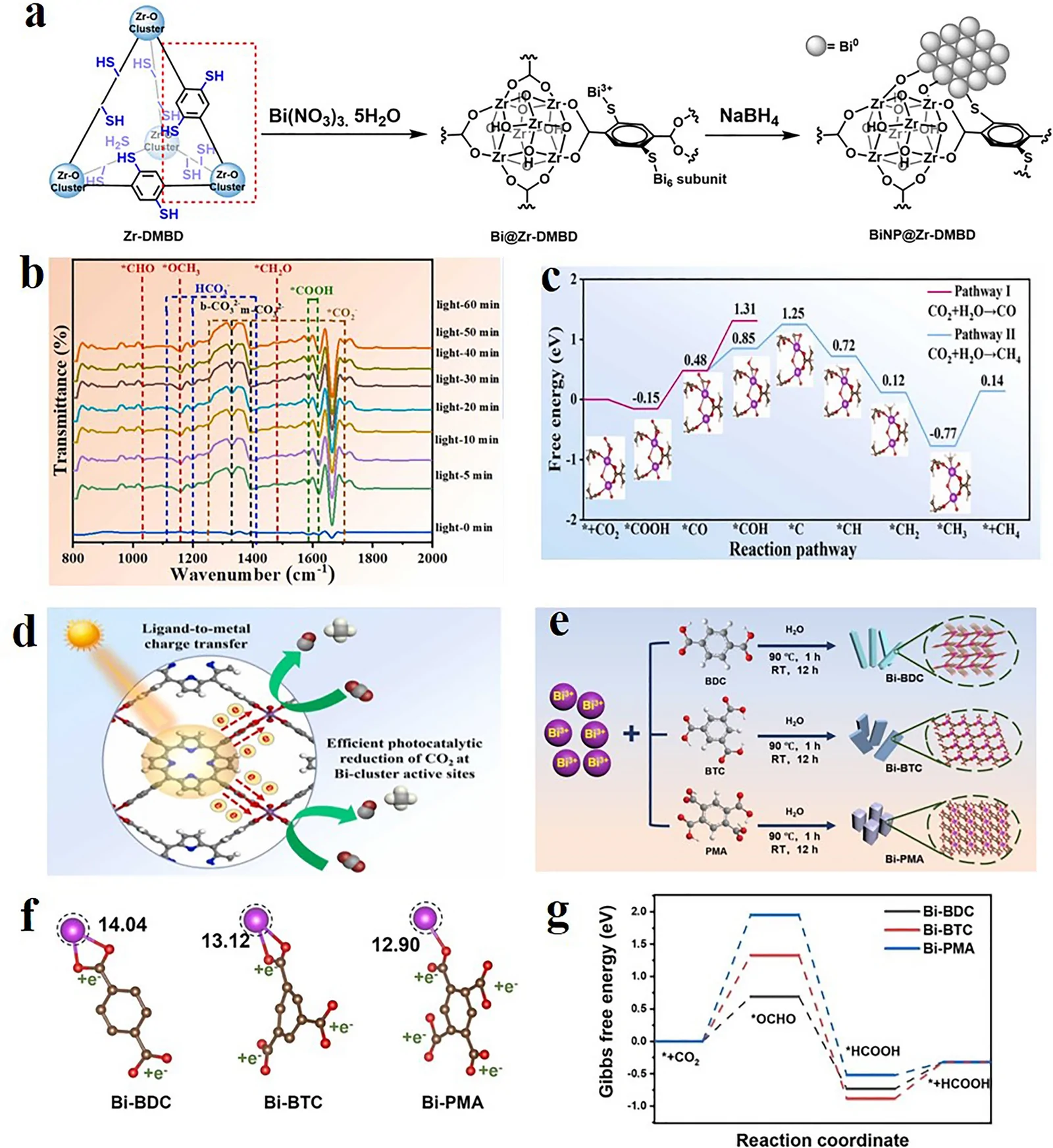
Multipathway regulatory mechanism of photoelectrocatalytic CO_2_RR of Bi-based MOFs with specific ligand structure. **a** Schematic presentation of chemical transformation of Zr-DMBD to BiNP@Zr-DMBD; [114] © The Royal Society of Chemistry 2025. **b** In situ FT-IR spectra of SC-Bi-PMOF-6 h under light irradiation after purging with CO_2_ and H_2_O vapor;** c** Gibbs free energy change (ΔG) and reaction pathways for photocatalytic CO_2_ reduction to CO and CH_4_ over Bi-PMOFs; **d** schematic of photocatalytic CO_2_ reduction on SC-Bi-PMOFs; **e** schematic illustration of the preparation procedure of Bi-MOFs; [115] © 2024 Elsevier B.V. All rights reserved. **f** Bader charge analyses of Bi–BDC, Bi–BTC, and Bi–PMA; **g** Gibbs free energy diagram for ECR to HCOOH process on Bi–BDC, Bi–BTC, and Bi–PMA. [116]. Copyright © 2025, American Chemical Society

The tight integration of metal nanoparticles into MOFs can achieve the customized function of composite material structures. Borah et al. [114] proposed a “bottle-in-ship” method to produce approximately 4.0 nm Bi NPs in zirconium-based MOF rich in thiol Zr-DMBD (2,5-dimercapto-1,4-phenyldicarboxylate), as shown in Fig. 12a. It was found that the combination of Bi NPs junctions into the Zr-DMBD framework depends on independent thiol groups. These mercaptans have two functions (i) to help bind precursors. Bi^3+^ prevents the formation of insoluble Bi methyl units (BiO) and (ii) controls the growth of Bi NPs. The resulting composites (denoted as BiNP@Zr-DMBD-1) exhibit stronger catalytic activity due to the strong interaction between Bi NPs and the sulfur-mediated organic linker, which facilitates the transfer of charge from Bi NP to the MOF matrix. In a flowing environment, after the electroreduction of CO^+^_2_ to formic acid, BiNP@Zr-DMBD-1 remains stable with a Faraday efficiency > 88% at a current density of 25 mA cm^−2^. This work opens up new avenues for developing MOFs rich in functional thiol groups into novel nanomaterials with a variety of catalytic and photocatalytic applications. Exploring a simple, rapid and efficient method for preparing MOFs with highly accessible active sites is of great significance for catalysis. Xu et al. [115] utilized the synergistic physicochemical properties of supercritical CO_2_ (scCO_2_) (SC-Bi-PMOFs) to structurally regulate Bi-PMOFs at the molecular level. They achieved rapid growth of Bi-PMOFs crystals at low temperatures and promoted the synthesis of 2D Bi-PMOF nanosheets through the chemical coordination of CO_2_. The prepared SC-Bi-PMOFs have the advantages of unique coordination environment, high density of active sites, and fast electron transfer ability, and have excellent photocatalytic CO_2_ reduction activity. In situ infrared testing of photocatalytic CO_2_ reduction explains the CO_2_ reduction process (Fig. 12b). Firstly, the chelated carbonate ions (m-CO_3_^2−^, b-CO_3_^2−^) and *CO_2_^−^ are adsorbed onto the surface of the catalyst. Through proto-coupled electron transfer, *COOH and HCO_3_^−^ are generated. Subsequently, further proto-coupled electron transfer is carried out to produce *CHO, *CH_2_O, and *OCH_3_ intermediates to achieve photocatalytic reduction of CO_2_ to CO and CH_4_. DFT calculations indicate that the adsorption process of CO_2_ on the surface of Bi-PMOFs is exothermic, and the Bi active sites can effectively capture and activate CO_2_. As shown in Fig. 12c, the CO_2_ adsorbed on the Bi sites of Bi-PMOFs is activated and spontaneously generates *COOH with a free energy of − 0.15 eV. Furthermore, the energy required for the conversion of *CO to *COH is 0.85 eV, which is lower than that required for the conversion of *CO to CO (1.31 eV), indicating that this catalytic reaction pathway can be further carried out to generate CH_4_. Based on the above results, the mechanism of SC-Bi-PMOFs photocatalytic CO_2_ reduction was proposed (Fig. 12d). Porphyrin ligands act as light collectors to generate electron–hole pairs. The electrons produced by light are rapidly transferred to the active sites of Bi. The electrons enriched at the Bi active site can effectively reduce CO_2_ to CO and CH_4_ (the charge transfer process from ligand to metal). Throughout the process, the presence of 2D Bi-PMOFs nanosheets with high-density active sites ensures efficient electron transfer, which plays a key role in the multielectron involved photocatalytic CO_2_ reduction. This research provides new ideas for the rapid and efficient construction of porphyrin-based MOFs materials with rich active sites, as well as for the further practical application of CO_2_ photocatalysis. Studying the ligand structure is equally crucial for improving the reduction performance of electrochemical CO_2_ catalysts based on the MOF. Zhao et al. [116] prepared three Bi-MOFs with different spatial structures using 1,4-phenyltricarboxylic acid (BDC), 1,3,5-phenyltricarboxylic acid (BTC), and 1,2,4,5-phenyltricarboxylic acid (PMA) as organic ligands and used them for the electrocatalytic reduction of CO_2_ to formate (HCOO^−^) (Fig. 12e). Among them, the selectivity of Bi–BDC for HCOO^−^ is the highest in the H-type electrolytic cell. In the flow cell, the selectivity of Bi–BDC for HCOO^−^ reaches 96%, and the local current density is as high as 105.36 mA cm^−2^. In situ Raman spectroscopy verified the formation of the key intermediate *OCHO during the electrocatalytic CO_2_RR process. DFT calculations show that the electron density of the Bi central site in Bi–BDC is the highest, as shown in Fig. 12f, indicating that the coordination environment affects the electron density of the Bi site in Bi-MOF due to different ligands. This phenomenon might be due to the fact that the carboxyl group is a strong electron-withdrawing group [117] and the electron accumulation of the carboxyl group can also be observed in the Bader charge distribution diagram. The higher the electron density of the metal active sites in the catalyst is, the more conducive it is to the activation and reduction of CO_2_ [118, 119]. Therefore, the electrocatalytic CO_2_RR performance of Bi–BDC is the best. To further study the mechanism of Bi–BDC catalysis, the Gibbs free energy of CO_2_ to generate HCOO^−^ in each process was calculated (Fig. 12g). Among all the reaction processes, it is generally believed that the step with the highest energy barrier is the rate-determining step of the entire reaction process. By comparing the reaction energy barriers of each step, the first hydrogenation process, that is, the generation process of the intermediate *OCHO, has the highest energy barrier and is considered to be the rate-determining step. The ΔG value of Bi–BDC is the lowest, indicating that Bi–BDC is more likely to generate the *OCHO intermediate. Therefore, it has better catalytic activity and HCOO^−^ selectivity in electrocatalytic CO_2_RR. Therefore, we believe that the high electron density of the Bi active site in Bi–BDC reduces the energy barrier, promotes the formation of the key intermediate *OCHO, and ultimately increases the FE_HCOO_^−^ of ECR. This study provides an in-depth understanding of the influence of ligand structure on the catalytic performance of MOFs and offers new strategies for the research of MOFs to design efficient MOF electrocatalysts.

The MOF material composite strategy significantly enhances the CO_2_ reduction performance of Bi-based catalysts through metal–ligand synergy and structural tunability. The high specific surface area and ordered channels of MOFs enhance CO_2_ adsorption, and the coordination microenvironment (such as Bi–N bonds) optimizes the stability of the intermediate *OCHO through electron transfer and reduces the reaction energy barrier. The confinement effect of MOFs can be dynamically complementary to the active sites of Bi (for example, the Cs_3_Bi_2_Br_9_/Bi-MOF heterojunction accelerates charge separation through the sharing of Bi atoms). However, the limitation lies in the high complexity of the synthesis of MOFs (for example, BiNP@Zr-DMBD requires precise control of the coordination between the thiol group and Bi^3+^), which makes large-scale preparation difficult. The proton transport resistance brought about by high porosity may trigger the hydrogen evolution side reaction (HER) at high current density. The application potential of MOFs is still considerable. However, it is still necessary to achieve efficient product synthesis within a wide potential window through ligand functionalization and interface conductivity regulation, as well as balancing pore structure design and charge transport kinetics.

### Inorganic Heterojunction Engineering

**Fig. 13:**
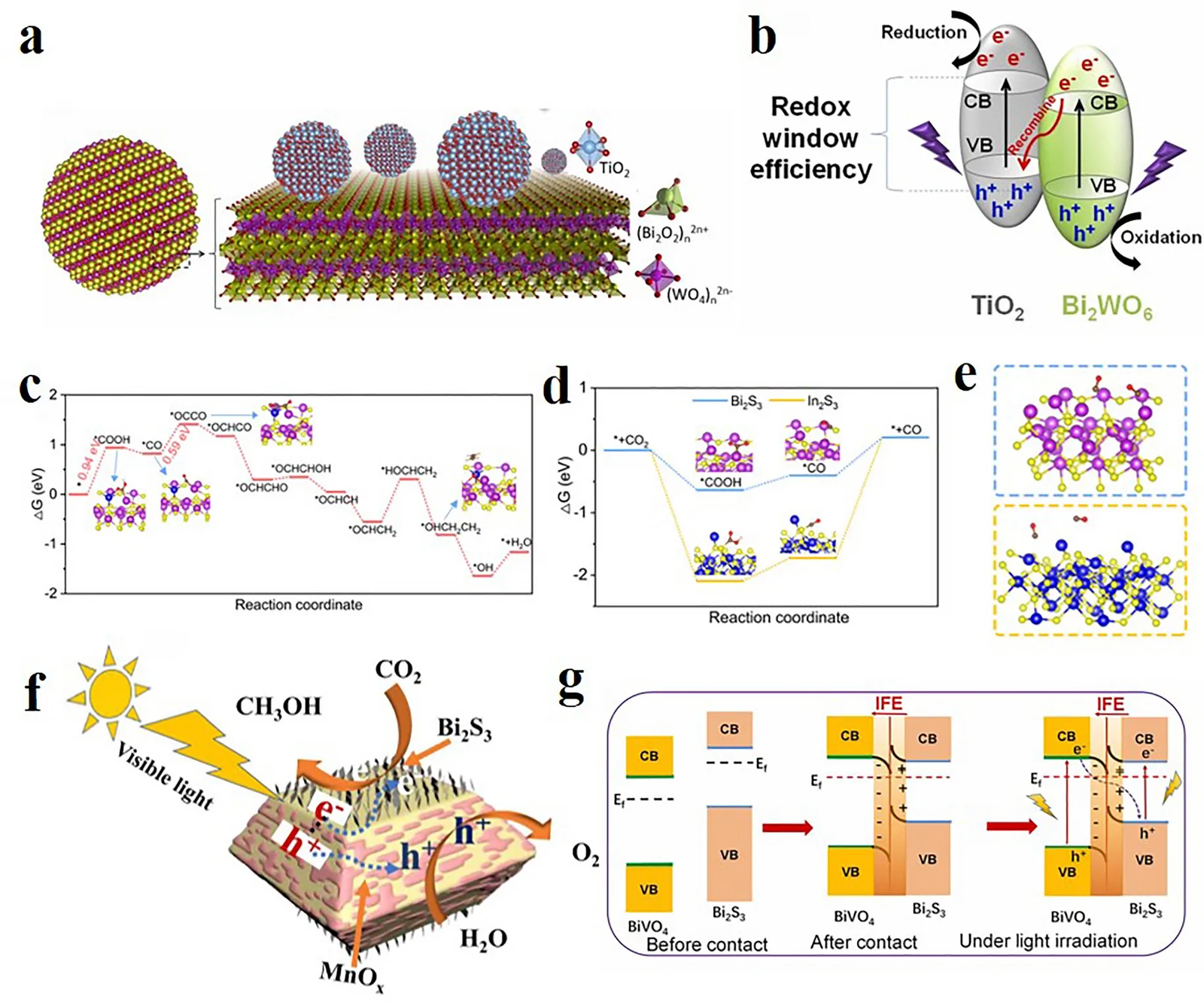
Heterogeneous interface engineering of Bi-based materials and inorganic semiconductors. **a** Schematic representation of the crystal structure and metal coordination of Bi_2_WO_6_ and TiO_2_ in BiW/Ti hybrids; **b** charge transfer mechanism in Bi_2_WO_6_/TiO_2_ heterojunctions [121]; © 2022 The Authors. Published by Elsevier B.V. Gibbs free energy diagrams for **c** CO_2_ reduction to C_2_H_4_ on the In–S_V_–Bi_2_S_3_ surface and **d** CO_2_ reduction to CO on pristine Bi_2_S_3_ and In_2_S_3_ surfaces; **e** configurations of two *CO adsorbed on Bi_2_S_3_ and In_2_S_3_ surfaces [122]; Copyright © 2023, American Chemical Society. **f** Schematic illustration of BiVO_4_/Bi_2_S_3_ heterojunction: the charge transfer and separation inducted by the internal electric field, and the formation of the direct Z-scheme heterojunction under visible light irradiation; **g** schematic illustration of the photocatalytic CO_2_ reduction process (Note: B/B and M/B/B refer to BiVO_4_/Bi_2_S_3_ and MnO_x_/BiVO_4_/Bi_2_S_3_, respectively [123]. © 2022 Elsevier Ltd

Complementary to the strategies of forming composite materials with organic frameworks such as COFs and MOFs, the heterogeneous interface engineering of Bi-based materials and inorganic semiconductors has opened up a new dimension of charge dynamics optimization. This inorganic–inorganic composite system reconstructs the carrier migration path through precise band matching, forming a triple synergistic mechanism with the molecular sieve separation effect of the organic framework and the coordination catalytic function of the metal–organic structure, jointly promoting the efficiency enhancement of the entire process from directional charge separation to surface reaction. Therefore, combining Bi-based catalysts with other inorganic materials, such as metal oxide/sulfide semiconductors, and constructing a heterostructure, is also an essential strategy to improve the catalytic performance. For those Bi-based photoelectrocatalysts, their quantum efficiency can be significantly improved by promoting visible light adsorption, charge transfer, and inhibiting carrier recombination. Furthermore, these heterojunctions can also promote the adsorption and activation of CO_2_, thereby increasing the turnover frequency [120] of CO_2_ molecules and thus improving the selectivity of photoreduction products. Collado et al. [121] prepared Bi–tungsten mixed oxides and Bi_2_WO_6_/TiO_2_ (BiW/Ti) heterojunctions by a simple hydrothermal method. The band matching in Raman tests and theoretical calculations proves that there is a strong electronic coupling between the interface of Bi_2_WO_6_ and TiO_2_ in the BiW/Ti hybrid, promoting charge separation. The close contact of the nanostructure can provide an efficient charge transport channel. As shown in Fig. 13a, experimental tests and theoretical calculations show that the selectivity of hybrid products changes and tends to produce more products requiring electrons. Among them, BiW/Ti50 (where 50 denotes the mass percentage of TiO_2_ in the catalyst) has the highest CH_4_ production, exceeding single Bi_2_WO_6_ and TiO_2_. Compared with bare Bi_2_WO_6_, the BiW/Ti hybrid has better photoactivity, which is attributed to more efficient charge transfer in the heterojunction, as illustrated in Fig. 13b. The charge transfer in the mixed material is enhanced and the charge transfer resistance is reduced under the light. In addition, due to the enhancement of the charge transfer in the heterojunction, the lifetime of photogenerated carriers is extended and the recombination rate is slowed down. These findings suggest that the increased photoactivity of the hybrid is due to enhanced charge transfer, which helps to create spatially separated photocatalytic sites. The spatially separated REDOX sites formed at the interface (TiO_2_ reducing CO_2_, Bi_2_WO_6_ oxidizing H_2_O) are at the core of the performance improvement. Yan et al. [122] synthesized Bi_2_S_3_@In_2_S_3_ photoheterostructure catalyst by one-step solvothermal method, in which Bi_2_S_3_ served as the photothermal material and provided photoexcited carriers simultaneously. Experimental studies combined with theoretical calculations (Fig. 13c-e) reveal multiple mechanisms of action for photocatalytic CO_2_ reduction. Photogenerated carriers trigger the H_2_O molecular-assisted CO_2_RR through the excited state relaxation process, and the temperature increase also promotes the reaction kinetics. A unique In–S_V_–Bi triatomic active center (S_V_ represents sulfur vacancy) is formed at the interface, and this special coordination environment significantly reduces the CO_2_ activation energy barrier and C–C coupling energy barrier. The active center significantly promoted the formation of *CO intermediates and their dimerization process, and finally realized the efficient generation of C_2_H_4_ products. The tight heterogeneous interface enhances the charge separation efficiency, and the photothermal effect accelerates the reaction kinetics. Wang et al. [123] successfully constructed a high-quality BiVO_4_/Bi_2_S_3_ heterogeneous interface on the BiVO_4_ (010) crystal surface by using selective epitaxy growth technology shown in Fig. 13f. Through theoretical calculation and experimental characterization, it is confirmed that a built-in electric field from Bi_2_S_3_ to BiVO_4_ is formed at the interface, driving the photogenerated carrier migration mechanism: BiVO_4_ CB electrons recombine with Bi_2_S_3_ VB holes, and the electron-rich state of Bi_2_S_3_ CB and the hole state of BiVO_4_ VB are preserved, which improves the carrier space separation efficiency. Furthermore, the MnO_x_ co-catalyst was selectively supported on the BiVO_4_ (110) crystal surface by photochemical deposition, and the MnO_x_/BiVO_4_/Bi_2_S_3_ ternary heterostructure was constructed, as shown in Fig. 13g. Compared with BiVO_4_, the light absorption performance of the BiVO_4_/Bi_2_S_3_ nanostructure is significantly enhanced. After the deposition of the MnO_x_ co-catalyst (MnO_x_/BiVO_4_/Bi_2_S_3_), the light absorption performance is further slightly improved. This is because Bi_2_S_3_ and MnO_x_ have smaller band gap (E_g_) energy than BiVO_4_, and Bi_2_S_3_ has stronger absorption characteristics than BiVO_4_, which enhances the light absorption of the BiVO_4_/Bi_2_S_3_ heterostructure and broadens the absorption edge [124, 125]. In particular, Bi_2_S_3_ is a direct bandgap semiconductor with a small band gap energy of 1.36 eV that owns a very high light absorption coefficient (> 104 cm^−1^ for λ < 780 nm, and > 105 cm^−1^ for λ < 500 nm) and reasonable energy conversion efficiency. Therefore, the MnO_x_/BiVO_4_/Bi_2_S_3_ hybrid structure has good light capture ability and photoinduced electron transport characteristics, and is regarded as an efficient photoactive composite structure. The system exhibits excellent performance in the CO_2_RR to methanol. The key mechanism is the reduction of the C–C coupling barrier at the Mn^3+^ site by *d-p* orbital hybridization. Among various semiconductors, BiOI has a narrow band gap of 1.8 eV and can respond to visible light with wavelengths greater than 600 nm [126]. Furthermore, BiOI has a strong reducing ability and is therefore widely used in CO_2_ reduction [127], etc. However, the photoreduction of CO_2_ requires the participation of protons, and protons usually come from the oxidation of water. Therefore, the photocatalyst must have a strong oxidation capacity in order to obtain protons from water [128]. Bi_2_MoO_6_ has a strong oxidation capacity and has been widely studied in many fields. In order to comprehensively enhance the performance of the catalyst, the two are combined to construct a stepped structure (S-scheme) heterojunction composite material, thereby achieving rapid charge transport and increasing the number of active sites. Based on this, Wang et al. [129] successfully synthesized 2D Bi_2_MoO_6_/BiOI heterojunctions by in situ growing Bi_2_MoO_6_/BiOI nanosheets on Bi_2_MoO_6_ nanosheets, significantly improving the CO_2_ photoreduction capability. The 2D structure of two components increases the interface area of the composite material and provides an important charge transfer channel, which facilitates rapid charge separation. At the same time, the S-scheme heterojunction enhances the redox capacity of Bi_2_MoO_6_/BiOI van der Waals heterojunction composites, thus providing a strong driving force for CO_2_ photoreduction.

**Fig. 14:**
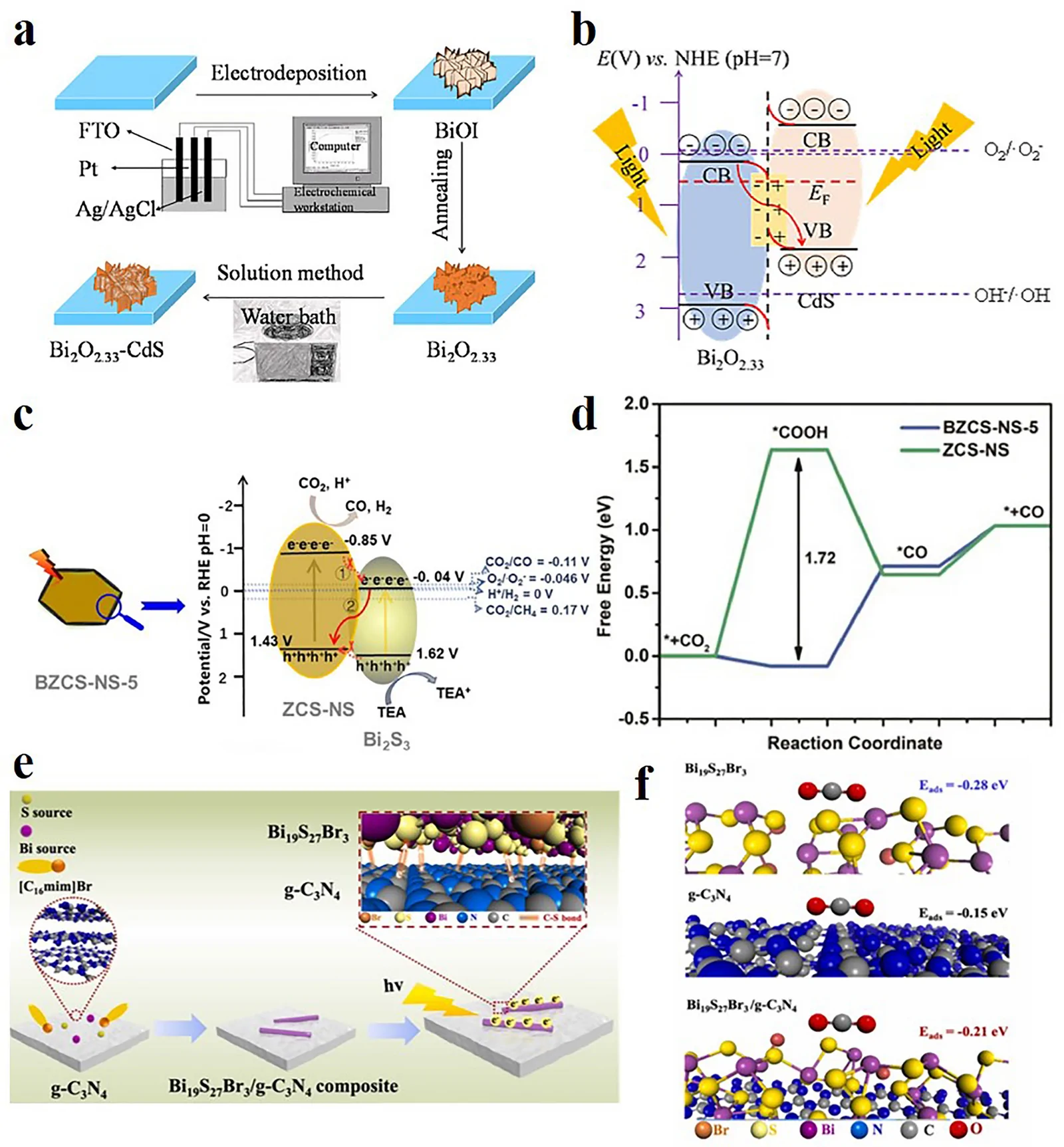
Design and mechanistic elucidation of Bi-based heterojunctions. **a** Schematic synthesis process of the composite Bi_2_O_2.33_-CdS photocatalyst; **b** schematic illustration of Bi_2_O_2.33_-CdS heterojunction charge transfer mechanism [130]; © 2022 Published by Elsevier Ltd on behalf of the editorial office of Journal of Materials Science & Technology. **c** Proposed mechanism for CO_2_ photoreduction catalyzed by Bi_2_S_3_/ZnCdS; **d** free energy required for photoreduction of CO_2_ to CO of ZnCdS and Bi_2_S_3_/ZnCdS [131]; © 2022 Chongqing University. Production and hosting by Elsevier B.V. on behalf of KeAi. **e** Schematic illustration for the preparation and CO_2_ photoreduction process of the Bi_19_S_27_Br_3_/g-C_3_N_4_-5 composite; **f** DFT calculations of adsorption energies for Bi_19_S_27_Br_3_, g-C_3_N_4,_ and Bi_19_S_27_Br_3_/g-C_3_N_4_ composite [132]. © 2022 Elsevier B.V

The direct Z-heterojunction can greatly improve the separation efficiency of the material and the light-generating carrier, maximize the redox capacity of the prepared Bi-based photocatalyst, and increase photocatalytic activity. Wang et al. [130] prepared Bi_2_O_2.33_-CdS direct Z-scheme heterojunction. Bi_2_O_2.33_ core nanosheets were prepared by electrodeposition, as shown in Fig. 14a, and then the heterojunction was prepared by the annealing process. The CdS shell was then deposited on the Bi_2_O_2.33_ nanosheet by the solution method. In this process, a uniform and continuous integrated CdS shell was formed by using a suitable concentration of CdCl_2_ solution. Non-stoichiometric Bi_2_O_2.33_ is rich in oxygen vacancies, which connect Bi_2_O_2.33_ during hybridization and CdS form stable interfacial contacts, and XPS analysis shows a decrease in the oxygen vacancy/lattice oxygen ratio, indicating that the Vo are partially filled. A space charge region and an internal electric field from CdS (+) to Bi_2_O_2.33_ (–) are formed at the interface, driving a direct Z-scheme charge transfer process, as shown in Fig. 14b. Bi_2_O_2.33_-CdS showed excellent CO_2_ reduction photocatalytic performance, mainly due to the good photoinduced charge separation and transport efficiency in the direct Z-scheme heterojunction. The photocatalytic CO_2_ reduction capacity of Bi_2_O_2.33_-CdS is significantly higher than that of single Bi_2_O_2.33_ or CdS. Vo promote interfacial bonding and are the core factor for performance improvement. Zou et al. [131] prepared ZnCdS nanoplates with a unique heterostructure with a Bi_2_S_3_ end edge (Bi_2_S_3_/ZnCdS) through an easy cation exchange pathway to achieve a controlled photocatalytic CO_2_ conversion. When CO_2_ molecules are present in the system, the free energy of Bi_2_S_3_/ZnCdS is also low, which is conducive to the adsorption and activation of CO_2_ molecules, and the subsequent reduction to COOH* intermediates produce the final product CO (Fig. 14c, d). The optimized Bi_2_S_3_/ZnCdS photocatalyst has good CO_2_ photoreduction ability. The CO yield is about 513.2 ± 5.1 μmol^−1^ h^−1^ and the selectivity is about 91.0%, which is one of the most active sulfide photocatalysts in the literature. Due to the formation of Z-type heterostructure between Bi_2_S_3_ and ZnCdS, the separation and migration of photocarriers are accelerated, and thus excellent photocatalytic performance is obtained. Zhao et al. [132] synthesized a novel interfacial C–S bond modulated Z-scheme heterojunction Bi_19_S_27_Br_3_ /g-C_3_N_4_ composite material as shown in Fig. 14e, by using an ionic liquid-assisted solvothermal method, which used C–S bonds as high-speed channels to accelerate the transfer of photogenerated electrons from g-C_3_N_4_ to Bi_19_S_27_Br_3_, and the electrochemical impedance is reduced, indicating an improvement in charge separation efficiency. More excited reducing electrons are provided to the Bi_19_S_27_Br_3_ surface, and the Bi_19_S_27_Br_3_ surface has a lower CO_2_ adsorption energy. To explore the adsorption capacity of the material for CO_2_, the CO_2_ adsorption process was simulated by DFT calculation. As shown in Fig. 14f, after material structure optimization, it is calculated that the CO_2_ adsorption energy E_ads_ of Bi_19_S_27_Br_3_ is -0.27 eV, which is higher than that of g-C_3_N_4_ (-0.15 eV). The E_ads_ value of Bi_19_S_27_Br_3_/g-C_3_N_4_ composite is -0.21 eV, which is higher than that of g-C_3_N_4_, indicating that the CO_2_ adsorption capacity of Bi_19_S_27_Br_3_/g-C_3_N_4_ composite is enhanced than that of g-C_3_N_4_, which is more conducive to CO_2_ emission reduction transformation. Without the addition of sacrifice agents and photosensitizers, the photocatalytic CO_2_ conversion yield of Bi_19_S_27_Br_3_/g-C_3_N_4_ is 5 times and 4 times higher, respectively, than that of Bi_19_S_27_Br_3_ and g-C_3_N_4_.

Inorganic heterojunction engineering enhances the CO_2_ reduction performance of Bi-based catalysts through band matching and interinterface charge regulation: The built electric field in the heterojunction (such as the BiVO_4_/Bi_2_S_3_ interface) drives the spatial separation of photogenerated carriers, reduces the recombination rate, and lowers the CO_2_ activation energy barrier through interinterface active sites (such as the In–S_V_–Bi triatomic center). However, the interface contact quality of heterojunctions depends on precise synthesis control, and photocorrosion problems (such as poor stability of CdS) may limit long-term stability. The catalytic performance can be further enhanced by synergistically catalyzing the reduction of CO_2_ with other strategies, such as atomic doping.

### Crystal Face Engineering

**Fig. 15:**
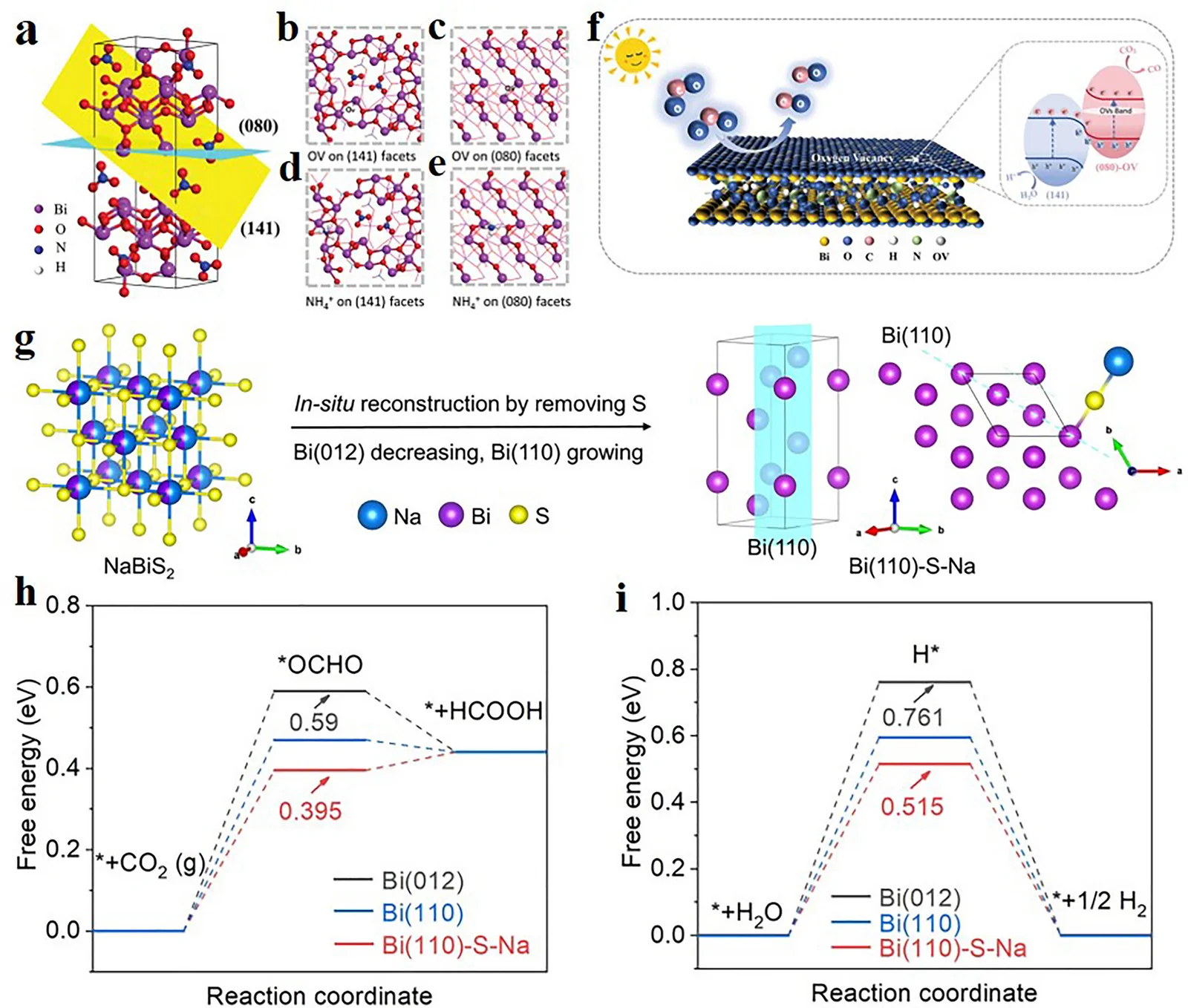
Crystallographic facet engineering and in situ phase evolution in Bi-based electrocatalysts. DFT studies of Bi_5_O_7_NO_3_ crystallographic facets: **a** Crystal plane model of (141) and (080) facets. Bi: purple; O: red; N: blue; H: white; **b, c** oxygen vacancy models on (141) and {080} facets; **d, e** adsorption models of NH_4_^+^ adsorbed on (141) and (080) facets of Bi_5_O_7_NO_3_; **f** schematic illustration of possible carrier transfer path between (141) and (080)-OV facets [136]; © 2024 Wiley‐VCH GmbH. **g** Scheme of phase transition from initial NaBiS_2_ nanodots to final Bi (110)-S-Na nanosheets under in situ CO_2_ electroreduction at -1 A‧cm^−2^. Energy diagrams of **h** formate and **i** H_2_ pathway on these three models [137]. © 2023 Elsevier Inc

In addition to hybridizing with organic frameworks and inorganic materials, crystal plane engineering provides a new atomic-level regulation method for improving the performance of Bi-based catalysts. Adopting crystal face engineering to optimize the physicochemical properties of catalysts at the atomic level [133] is also a novel strategy to improve the CO_2_RR performance. Crystal face engineering has become one of the hot spots due to its unique arrangement of surface atoms on a particularly exposed surface [134, 135]. Crystals tend to form stable crystal faces with low surface energy during the conventional growth process, and these thermodynamically stable surfaces usually show low reactivity, which makes it difficult to meet the needs of efficient catalysis. Therefore, the directional design and control of specific crystal faces through surface engineering strategy is of great significance for improving catalyst performance. Li et al. [136] proposed a method of catalyst modification with the help of crystal face engineering. They synthesized a new Bi_5_O_7_NO_3_ crystal with a customized (080) face exposed by an NH_4_^+^-assisted self-limiting structure. As shown in Fig. 15a-e, it has been demonstrated that NH_4_^+^ ions are selectively adsorbed on the (141) surface, which in turn induces the growth of the desired (080) crystal surface, conducing to the generation of Vo. As shown in Fig. 15f, controlled Vo concentrations affected by different exposure surfaces can optimize the relative positions of Fermi levels and transform the photoelectron transport path between (141) and (080)-Vo surfaces from type II to type S, thus triggering fast charge transport channels and effectively inhibiting electron–hole recombination. The DFT calculation verifies that the energy barrier formed by *COOH on Bi_5_O_7_NO_3_-(080)-Vo is the lowest, thus promoting the generation of CO. The photocatalytic CO_2_ reduction efficiency of well-designed Bi_5_O_7_NO_3_ crystals with the optimal (080)/(141) ratio is 3.8 times higher than that of conventional Bi_5_O_7_NO_3_ crystals with the predominant (141) plane. Peng et al. [137] designed and synthesized metallic Bi nanosheets with highly exposed (110) surfaces and co-modified with sulfur anions and sodium cations, shown in Fig. 15g. DFT calculations were first used to investigate the *OCHO and H* formation energies of those three models. Bi (110) showed a lower *OCHO formation energy of 0.47 eV than Bi (012) (Fig. 15h), suggesting the favorable formate pathway. After co-modified by S^2−^ and Na, this value was further reduced to 0.395 eV. Similarly, the H* formation energy on Bi (110)-S-Na was decreased to 0.515 eV from 0.761 eV on Bi (012) and 0.595 eV on Bi (110) (Fig. 15i), suggesting the easier water activation to form adsorbed H* intermediates. The H* was anchored at Bi that was close to the site with *OCHO adsorption to fully utilize H* for hydrogenation of adjacent *OCHO to form formate. Moreover, the formation energies of *OCHO and H* with two pairs of S-Na modification on Bi (110) surface were calculated as 0.329 and 0.504 eV, respectively, suggesting the easier generation of formate and H* than that with one pair of S-Na modification.

**Fig. 16:**
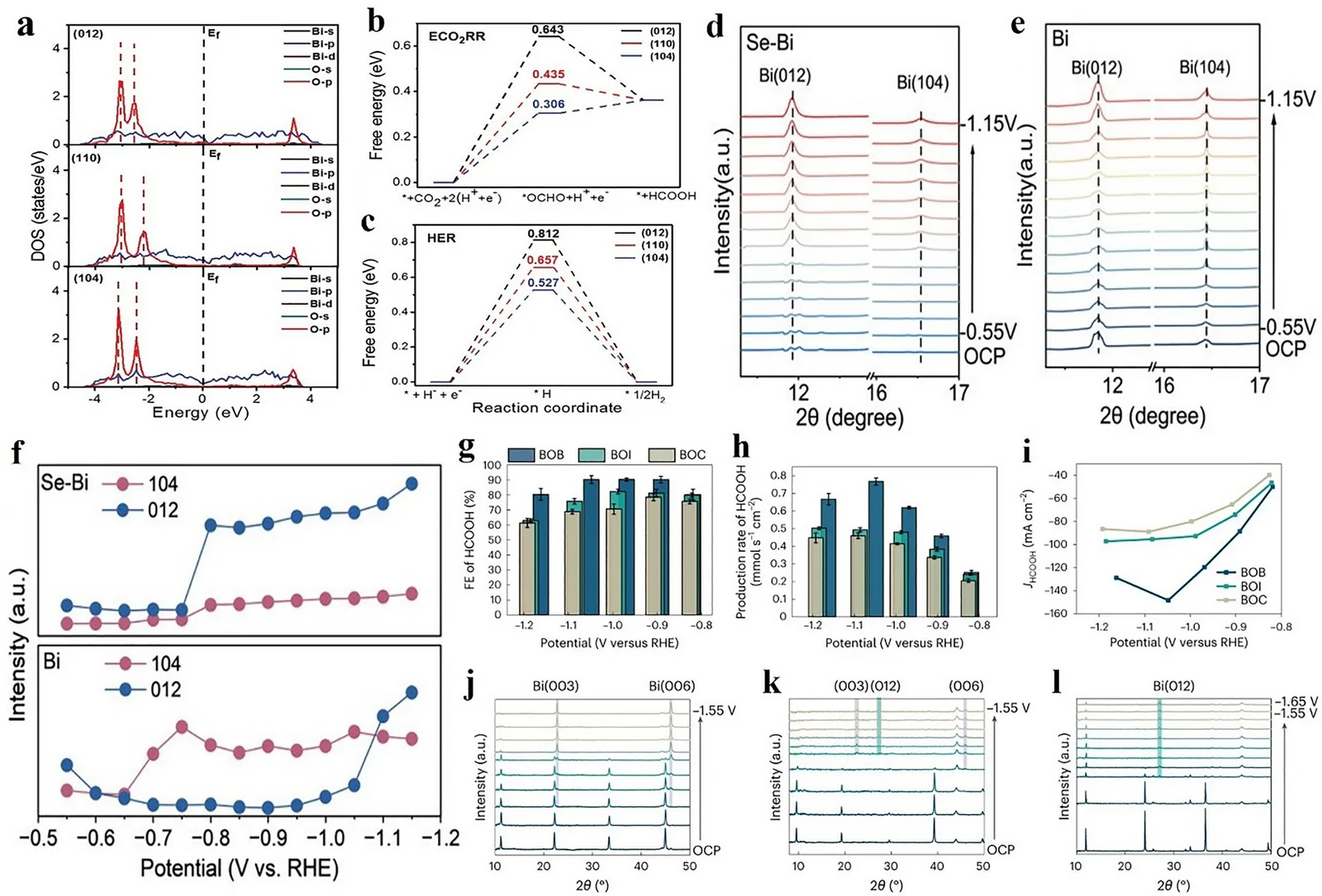
Surface engineering of Bi-based catalysts and dynamic phase transformation synergistically regulate the electroreduction of CO_2_ to formic acid. **a** PDOS of the O atoms for *OCHO intermediate and Bi atoms of the adsorption sites for different Bi surfaces; **b** free energy diagrams of formate formation by eCO_2_RR on different Bi surfaces; **c** free energy diagrams of HER on different Bi surfaces [138]; © 2021 Wiley‐VCH GmbH. Potential-dependent in situ XRD patterns of **d** Se–Bi and **e** Bi in CO_2_-saturated 0.5 M KHCO_3_ solution measured under synchrotron radiation at 0.6333 Å; **f** diffraction intensities of the Bi (104) and Bi (012) facets in the Se–Bi and Bi catalysts as a function of the applied potential in CO_2_-saturated 0.5 M KHCO_3_ solution [139]; © 2025 Wiley‐VCH GmbH. **g, h** Comparison of FE and production rate of HCOOH in BiOBr, BiOI and BiOCl. Error bars were obtained by measuring the liquid products in triplicate, and the center value for the error bars is the average of the three independent measurements; **i** corresponding partial current density in CO_2_-saturated 1 M KHCO_3_ electrolyte solution using a GDE flow cell; **j-l** potential-dependent in situ XRD patterns of BiOBr (**j**), BiOI (**k**) and BiOCl (**l**) in CO_2_-saturated 0.1 M KHCO_3_ solution, plotted in arbitrary units (a.u.) versus diffraction angle 2θ [140]. Copyright © 2023, The Author(s)

Bi is a topological crystalline insulator, which has gapless topological surface states protected by a specific crystalline symmetry that strongly depends on the facet. Chai et al. [138] developed Bi rhombic dodecahedra (RDs) with exposed (110) and (104) faces, which exhibited very low overpotential (120 mV) and high selectivity (Faradi efficiency > 92.2%) within a wide range of formic acid bias current densities (from 9.8 to 290.1 mA cm^−2^). This makes the energy efficiency of generating formic acid in the entire gas diffusion pool as high as 69.5%. Compared the major peaks of *P-states* of O atoms in the *OCHO intermediate with the Bi atoms on which it adsorbed, Fig. 16 clearly shows the shift under the maximum valence band of the Bi (012) surface. Besides, there is only a small displacement on the Bi (110) surface and almost perfect overlap on the Bi (104) surface. These results further prove that the binding strength of the *OCHO intermediate on the Bi (104) and Bi (110) surfaces is stronger than that on the Bi (012) surface. Therefore, compared with the Bi (012) surface, the corresponding adsorption free energies of the *OCHO intermediate on the Bi (110) and (104) surfaces decreased by 0.208 and 0.337 eV, respectively, as shown in Fig. 16b, which led to a significant reduction in the overpotential of formate on Bi RDs. The adsorption free energy of *OCHO on the Bi (104) surface is smaller than that on the Bi (110) surface, which can be attributed to the higher surface energy of the former, which is conducive to reducing the energy of intermediate adsorption. Furthermore, on the Bi (110) and (104) surfaces, the adsorption free energy difference of *H for HER and *OCHO to form formic acid is greater than that on the (012) surface (Fig. 16b, c). The Bi (110) and (104) surfaces are more inclined to adsorb *OCHO rather than *H. The non-trivial surface state on the (110) surface and the trivial surface state with small voids on the (104) surface enhance and stabilize the adsorption of *OCHO and reduce the competitive adsorption of *H. This leads to a significant improvement in the selectivity of formic acid over a wide range of current densities. The robustness, high activity and selectivity, and high full-cell energy efficiency of Bi RDs enable potential applications in high-rate formate production and efficient energy storage of intermittent renewable electricity. In another study, Li et al. [139] simultaneously exposed the (104) and (012) crystal planes of Bi. They modified the pure Bi metal catalyst with selenium as the target element. The phase structure under practical application conditions was evaluated by in situ XRD. The selenium–bismuth catalyst exhibits potential-dependent catalytic performance, and the potential-related in situ XRD reveals the structural evolution. Diffraction characteristics were collected at every -0.05 V_RHE_ (Fig. 16d, e). The open-circuit potential (OCP) curve of the catalyst material was detected on the glassy carbon electrode. For the Se–Bi catalyst, there was no obvious crystal plane exposure, and only the characteristic peak of glassy carbon was recorded. As the reduction potential gradually decreases, the catalyst begins to expose the three main planes of Bi [i.e., the (012) and (104) planes] at -0.80 V. Meanwhile, as the negative potential increases, the intensities of these peaks also gradually increase. For the Bi catalyst, under OCV conditions, the (012) and (104) surface of the Bi metal is exposed to a certain extent. However, with the change of potential, the facets and the corresponding peak intensities also change. To quantify the influence of voltage variation on the exposed surface, a curve was constructed to show the correlation between the peak intensities of the (012) and (104) surfaces of Bi and the application potentials of the two catalysts (Fig. 16f). For Bi metal, the peak strength of the (104) surface begins to increase at -0.65 V_RHE_. However, the intensity of the (012) facet remained unchanged before −1.05 V_RHE_, and then began to gradually increase. In contrast, for the Se–Bi catalyst, the peak intensities of both the (012) and (104) surfaces began to increase at -0.75 V_RHE_. The growth of the Bi (104) facet effectively promoted the rapid growth of the Bi (012) facet. Combined with the electrocatalytic performance of the Se–Bi and Bi catalysts, these results indicate that the potential effects on the crystal facets of Bi influence the proportions of formate and H_2_ product. And the doping of Se and its structural reconstruction during the reduction process will affect the co-exposure process of different crystal planes of Bi. Compared with Bi metal, the (012) and (104) surfaces of Se–Bi are exposed simultaneously. This inhibited the competitive HER reaction on the Se–Bi catalyst, thereby increasing the yield of formate. This study proposes a crystal plane engineering strategy through doping of nonmetallic elements to achieve precise operation of the rate-determining step in the catalytic process, providing a new strategy for designing efficient CO_2_RR catalysts. In addition, Yang et al. [140] found that halogens can lead to the exposure of specific crystal planes of Bi. They first synthesized layered {001} oriented Bi oxide halide nanosheets (referred to as BiOX, X = Cl, Br or I), and used them as a platform for in situ monitoring the structure and active sites of Bi-based electrocatalysts in CO_2_RR. The kinetic process of in situ conversion of BiOX catalyst into active metal Bi electrocatalysts was tracked by in situ XRD and other test methods, as shown in Fig. 16g-i. It was found that the in situ activated Bi electrocatalyst, under the guidance of halides, selectively exposes specific crystal planes during the catalytic process: Br^−^ promotes the exposure of Bi (003), Cl^−^ leads to the dominant Bi (012) plane, and I^−^ generates a mixture. Furthermore, they linked the crystal plane exposure to the catalytic performance, as shown in Fig. 16j-l. Compared with the RHE, the BiOBr has a maximum HCOOH selectivity of 91% at a current density of 148 mA cm^−2^ of −1.05 V. At a potential of −1.09 V vs. RHE, BiOCl exhibited a Faraday efficiency of 69% HCOOH at a current density of 88 mA cm^−2^. When the current density of -1.08 V is 95 mA cm^−2^, between BiOBr and BiOCl, the selectivity of BiOI for HCOOH is 76%. This indicates that in electrocatalytic CO_2_RR, the in situ formed substrate Bi (003) plane has stronger catalytic activity than the stepped Bi (012) site. Furthermore, after the reduction begins, the reconstruction of BiOCl proceeds rapidly, indicating a transient transition state, while the transition states of BiOBr and BiOI last longer. This indicates that during the catalytic process, the halogens in BiOX affect the selective surface exposure of the formed Bi by regulating the reconstruction rate. This work provides new ideas for further understanding the active sites and structural evolution of Bi-based electrocatalysts in the electrocatalytic CO_2_ conversion.

Crystal face engineering optimizes the CO_2_ reduction performance of Bi-based catalysts by directionally exposing highly active crystal planes (such as Bi (110), Bi (104)), and regulates the adsorption energy of key intermediates by specific crystal planes, thereby reducing the reaction energy barrier and improving the selectivity of formic acid. However, high-energy crystal planes have thermodynamic instability (for example, in situ reconstruction of BiOX may cause dynamic changes in crystal planes), and the synthesis relies on precise condition control. The coupling of crystal planes with defects/doping (such as the synergy between oxygen vacancies in Bi_5_O_7_NO_3_-(080)-Vo and the charge transport path of crystal planes), as well as the co-exposure of polycrystalline planes (such as the (012)/(104) in Se–Bi catalysts), can more efficiently synergistically catalyze the CO_2_RR. In subsequent studies, we should reasonably balance the stability of crystal planes and the dynamic reaction microenvironment, and the gradient design of polycrystalline planes can be explored to achieve efficient multiproduct synthesis within a wide potential window.

### Alloying and Polarization Engineering

**Fig. 17:**
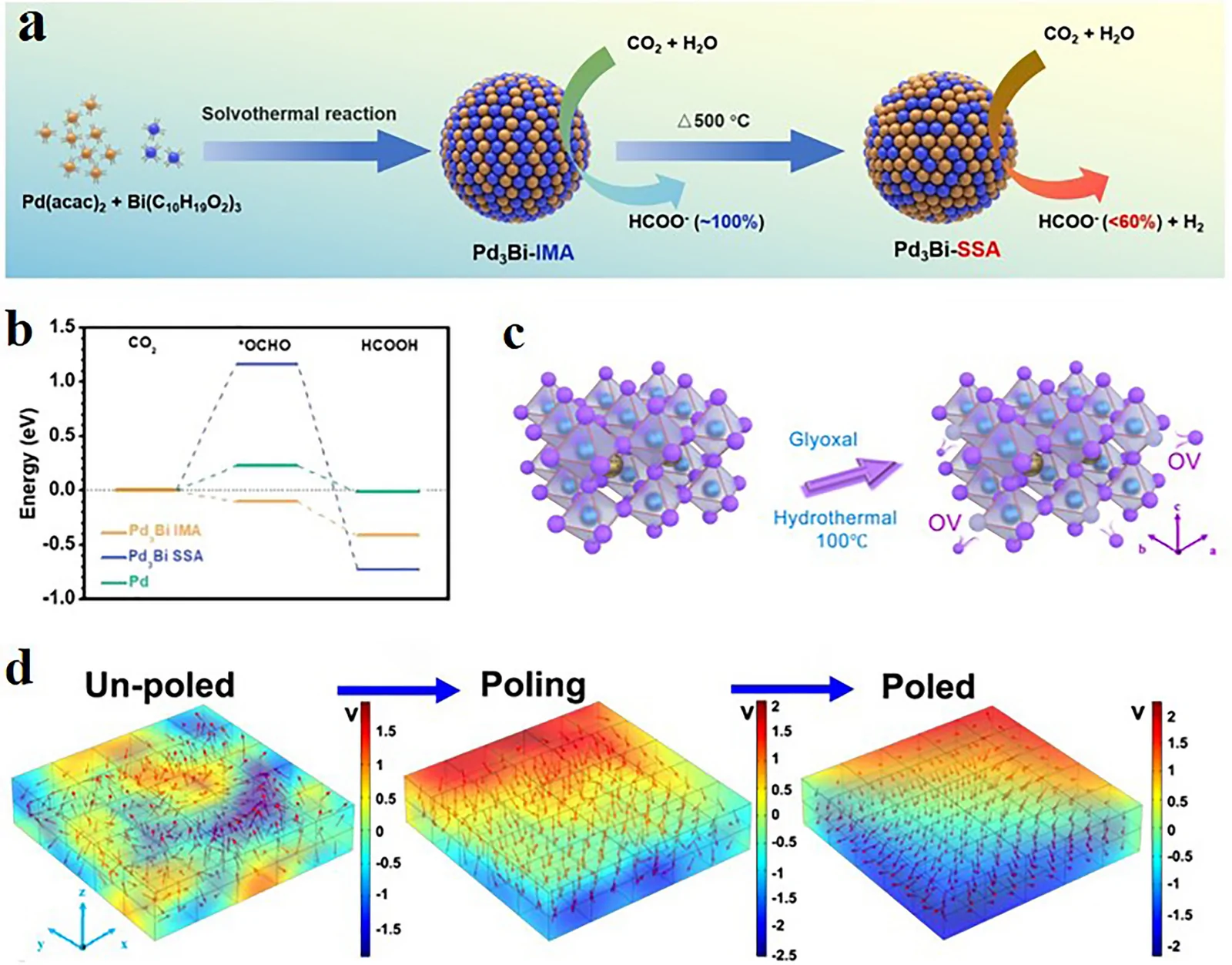
Synergizing alloy synthesis and ferroelectric polarization for efficient CO_2_-to-formate electroreduction. **a** Schematic synthetic procedure for Pd_3_Bi IMA and Pd_3_Bi SSA; **b** energetic trend of CO_2_RR to formic acid on Pd_3_Bi IMA, Pd_3_Bi SSA and pure Pd [144]; © 2021 Wiley‐VCH GmbH. **c** Schematic illustration for the formation of surface oxygen vacancies on Bi_3_TiNbO_9_. Blue, gold, and purple spheres represent Ti/Nb, Bi and O atoms, respectively. **d** COMSOL simulation of polarization-induced electric field on Bi_3_NbTiO_9_ sheets: un-poled, intermediate poled, fully poled. The red arrow represents the polarization direction of a single domain [151]. Copyright © 2021, The Author(s)

Except for the above-mentioned five high-quality structural regulation strategies, the alloying and polarization engineering strategies have further opened up new paths for the reconstruction of electronic state density and the regulation of dynamic responses [141, 142]. Through the atomic orbital hybridization of intermetallic alloys and the interface charge redistribution of polarized materials, a dual-cycle regulation system of “static electronic structure–dynamic charge transport” is formed in combination with the aforementioned spatial structure optimization strategy, achieving full-chain electronic coupling in key steps such as molecular adsorption activation (framework confinement), intermediate stabilization (crystal surface effect), and product desorption (defect enrichment). It provides a cross-scale solution for breaking through the triple trade-off of “activity–selectivity–stability” of CO_2_RR. In contrast to more common solid-solution alloys, intermetallic alloys have well-defined atomic arrangements [143] but are challenging in synthesis. As shown in Fig. 17a, Jia et al. [144] prepared intermetallic Pd_3_Bi nanocrystals with uniform size by a simple solvothermal method. These nanocrystals can be transformed into solid-solution alloys by a thermally annealed phase while maintaining similar composition and size. In 0.1 M KHCO_3_ aqueous solution, compared to a reversible hydrogen electrode, the intermetallic compound Pd_3_Bi can selectively reduce CO_2_ to formate, even at a low temperature. It also has a high selectivity of 100% for the target product and shows good stability under potential conditions of −0.35 V. In contrast, the HCOOH selectivity of solid-solution alloys is only 60%. This unique dependence of material properties on the phase state can be further analyzed with the help of theoretical simulations, as shown in Fig. 17b. The simulation results show that the crystal arrangement of Pd and Bi atoms can effectively inhibit CO_2_ poisoning in intermetallic alloys, and enhance the adsorption of *OCHO intermediates in the process of electrochemical CO_2_ reduction to formic acid, thus improving the reaction efficiency and selectivity.

In recent years, great progress has been made in the study of promoting carrier transfer and inhibiting carrier recombination to enhance photocatalytic activity by using the polarization effect [145–147]. Polarity is a unique physical property that is prevalent in non-centrosymmetric (NCS) materials [148]. Bi-based NCS materials have attracted extensive attention from researchers due to their rich and diverse composition, unique electronic structure, diversified physical and chemical properties, mixed electronic band structure, and other factors, showing potential application value in materials science and photocatalysis research [149]. Li et al. [150] investigated the effect of polarization effect on the activity of photocatalytic CO_2_ reduction. They used the CTAB-assisted hydrothermal method to prepare ultra-thin Bi_2_MoO_6_ nanoplates and then used corona polarization post-treatment to enhance the ferroelectricity of Bi_2_MoO_6_. It was found that corona polarization and the formation of thin layer structure greatly enhanced the separation and transfer of photogenerated charge carriers and the adsorption of CO_2_, which jointly promoted the photocatalytic CO_2_RR performance and produced CO more than 10 times that of bulk Bi_2_MoO_6_ samples. This work provides an effective way to promote charge separation of particle photocatalysts for corona polarization and provides new insights for synergistic enhancement of CO_2_ photoreduction activity. In addition, Yu et al. [151] also improved CO_2_ photoreduction performance through the synergistic effect of ferroelectric polarization and Vo, as shown in Fig. 17c. This group first prepared the ferroelectric Bi_3_TiNbO_9_ nanosheets (BTNO NSs) and enhanced its ferroelectric polarization characteristics by corona polarization technology, which effectively promoted the bulk phase charge separation shown in Fig. 17d. At the same time, by reintroducing surface Vo, the light absorption range of the material is further expanded, and the adsorption and activation ability of CO_2_ molecules on the catalyst surface is enhanced. Most importantly, due to the pinning effect of Vo on ferroelectric domains, BTNO NSs can maintain excellent ferroelectric polarization properties, successfully solving the key challenge in the photocatalytic CO_2_ reduction process. This study not only highlights the importance of ferroelectric properties and controlled defect engineering but also emphasizes the key role of improving CO_2_ photoreduction performance by regulating the bulk phase and surface properties.

**Fig. 18:**
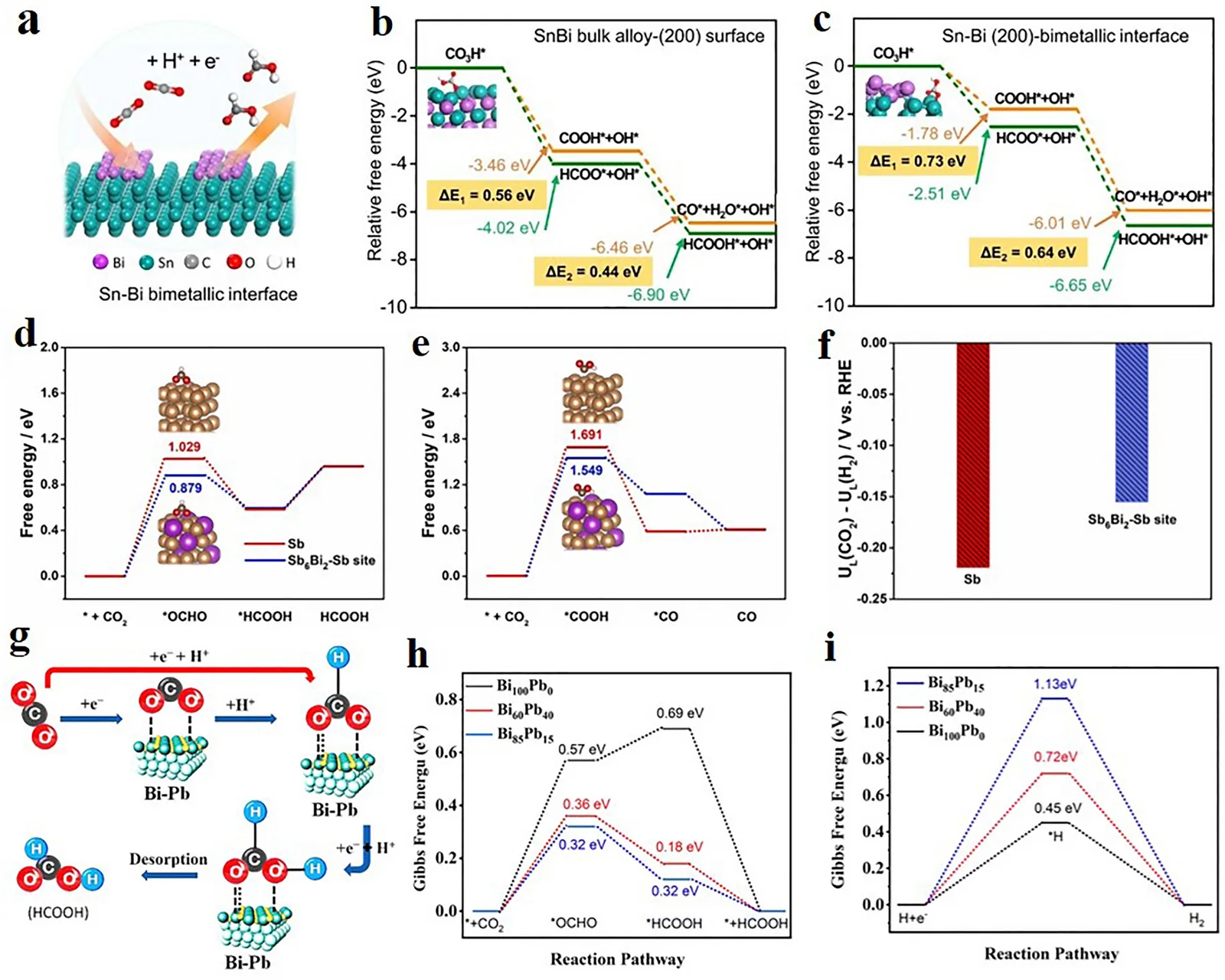
CO_2_RR regulation mechanism by Bi-based bimetallic interface engineering. **a** Scheme of CO_2_RR on Sn–Bi bimetallic interface. Gibbs free energy profiles of CO and HCOOH production pathways on **b** Sn–Bi alloy surface and **c** Sn–Bi bimetallic interface [152]; Copyright © 2022, The Author(s) The calculated Gibbs free energy diagrams for **d** CO_2_-to-HCOOH conversion and **e** CO_2_-to-CO conversion on the Sb site of Sb and Sb_6_Bi_2_ slabs. The gold, purple, red, white and brown spheres represent Sb, Bi, O, H and C atoms, respectively; **f** difference in limiting potentials for CO_2_ reduction and H_2_ evolution [154]; © 2022 Elsevier B.V. All rights reserved. **g** Proposed mechanism for CO_2_ conversion to formate using Bi–Pb electrodes. Calculated Gibbs free energy profiles for **h** formic acid generation and **i** H_2_ formation on Bi_85_Pb_15_, Bi_60_Pb_40_ and Bi_50_Pb_0_ [155]. © The Royal Society of Chemistry 2025

Binary Sn–Bi metal catalysts provide an effective method for adjusting the selectivity and activity of CO_2_–formic acid conversion. To fully understand the catalytic activity of the Sn–Bi system and the relationship between its electronic structure and bimetallic mode, Chen et al. [152] determined through calculation that the interaction of Sn with Bi at the interface is the most favorable structure for the formation of HCOOH. This structure has a weak Sn–C hybridization effect on the competition of COOH* intermediates and a superior Sn–O hybridization effect on the competition of HCOO*. Guided by theoretical discoveries and aiming to expose more active Sn–Bi interfaces, the Sn–Bi interface structure induced by nanofolds was designed through in situ electrodeposition experiments under CO_2_RR conditions. The correlation between the catalytic activity of the Sn–Bi binary system and the electronic properties and atomic modes was preliminarily evaluated by DFT calculations. The combination of Bi atoms and Sn atoms inhibits the generation of H_2_ and CO, thereby promoting the formation of formic acid. Compared with the pure Sn surface, the addition of Bi makes the HCOO* intermediate more easily adsorbed on the Sn–Bi surface, thereby promoting the formation of HCOOH. Nevertheless, the Sn–Bi binary system can be divided into two categories: one is to exhibit bulk ordered alloy crystal phases (referred to as Sn–Bi alloys), and the other is surface alloys whose interactions occur only in surface or subsurface regions (referred to as Sn–Bi bimetallic interfaces, Fig. 18a). Therefore, the Sn(200) surface was established because it was reported to be one of the most thermodynamically stable surfaces [153]. The Sn–Bi bulk alloy was described by using atomically uniform Bi-substituted Sn unit cells, and the (200) alloy surface was established and compared with the Sn–Bi bimetallic interface model. The influence of atomic pattern effect on the electrocatalytic activity of CO_2_RR was investigated and expounded through the bimetallic Sn–Bi interface and alloy (Fig. 18b, c): COOH* and HCOO* are the two main competing intermediates for the production of CO and HCOOH, respectively. Therefore, this study considered two pathways through these two intermediates, which originate from the hydrogenation of adsorbed bicarbonate (CO_3_H*) species. The free energy spectrum indicates that, due to the relatively low free energy of the HCOO* intermediate, forming HCOOH on the two surfaces is a favorable approach. Through the first proto-electron transfer reaction, the free energy difference (0.73 eV) of the intermediate products HCOO* and COOH* at the Sn–Bi bimetallic interface is greater than that (0.56 eV) on the surface of the Sn–Bi alloy. This difference indicates that the formation of the HCOO* intermediate is thermodynamically easier than that of COOH* in the former structure. Furthermore, for the second proton–electron transfer reaction that generates HCOOH* or CO*, the free energy difference (0.64 eV) between the two competing products at the Sn–Bi bimetallic interface is also greater than that on the surface of the Sn–Bi alloy (0.44 eV). XPS and in situ XAFS characterizations indicated that the Sn–Bi bimetallic interface optimally shifted the *p* band center of Sn upwards, and the corresponding valence electron consumption was moderate, resulting in the fragile adsorption of *COOH and the moderate adsorption of HCOO*. Therefore, the bias current density of formate is relatively high (up to 140 mA cm^−2^). High FE_formate_ (> 90%) remained within a wide potential window (-0.74 to -1.14 V vs. RHE), with durability (160 h). This design concept can also be extended to the high-activity and stable interface design of other bimetallic catalytic systems. Zhang et al. [154] reported for the first time the catalytic electroreduction of CO_2_ to formate by nanoporous (np) Sb–Bi solid-solution alloy. They prepared the np-Sb_8-X_Bi_X_ catalyst with adjustable composition (X = 0, 2, 4, 6, 8) by dealloying the rapidly solidifying Mg_92_Sb_8-X_Bi_X_ precursor band in tartaric acid. In the precursors with Mg and Mg_3_(Sb,Bi)_2_ phases, the more active Mg atoms were selectively eliminated, while the miscible Sb and Bi atoms were retained and rearranged to form a nanoporous Sb–Bi solid solution. The Sb–Bi interaction formed in the alloy induced the electrocatalytic conversion from HER to highly selective CO_2_RR. Compared with the single-metal Sb catalyst, the Sb–Bi alloy suppressed HER and promoted the CO_2_-to-HCOOH conversion. DFT calculations were conducted to elucidate the mechanism credited for the excellent CO_2_RR performance toward formate production on the Sb–Bi alloys. The calculation models are the Sb sites on the (012) plane of Sb slab and Sb_6_Bi_2_ solid-solution alloy, which could provide direct evidence for the activation of Sb for CO_2_RR. The Gibbs free energy change for *OCHO formation is 0.66–0.67 eV lower than that for *COOH formation on both the Sb and Sb_6_Bi_2_ slabs (Fig. 18d, e). This scenario indicates that the CO_2_-to-HCOOH conversion tends to occur through CO_2_ → *OCHO → HCOOH instead of CO_2_ → *COOH → HCOOH. The reaction pathway of CO_2_ → *COOH → CO is also suppressed in both models. Compared to the Sb slab, the energy barriers of the rate-determining steps (RDS) for HCOOH, CO, and H_2_ formation all decrease on the Sb_6_Bi_2_ surface, suggesting an easier stabilization of *OCHO, *COOH, and *H intermediates (Fig. 18d, e). Considering the competing reaction of HER, the difference in thermodynamic limiting potentials for CO_2_ reduction and H_2_ evolution has been considered to reflect the selectivity in CO_2_RR. It is denoted as U_L_(CO_2_)-U_L_(H_2_), in which U_L_ = -ΔG/e, and the ΔG is the Gibbs free energy change for RDS. A more positive value of U_L_(CO_2_)-U_L_(H_2_) means a higher selectivity for CO_2_RR. The Sb_6_Bi_2_ slab shows a larger value of U_L_(CO_2_)-U_L_(H_2_) than that on Sb, indicating that the bimetallic interaction-modified Sb sites have a better HCOOH selectivity than the unmodified Sb (Fig. 18f). On the other hand, DFT calculations were also conducted on the Bi sites of Sb_6_Bi_2_ slab. The results demonstrate that bimetallic interaction-modified Bi sites are also favorable for CO_2_-to-HCOOH conversion. Experimental and theoretical studies have shown that the Sb–Bi electron interaction can promote the stability of the *OCHO intermediate at the Sb and Bi sites and facilitate the conversion of CO_2_ to HCOOH. This work reveals that bimetallic interactions have a significant impact on regulating the intrinsic catalytic activity of materials. Farid et al. [155] also utilized the dealloying strategy and based on the inherent chemical properties of the ternary metal components, introduced the synthesis of two different self-supporting Bi–Pb bimetallic catalysts, which have simple bicontinuous structures and compositions. Among them, the reticular Bi_85_Pb_15_ alloy (Bi_85_Pb_15_) has hollow areas, and the lamellar Bi_60_Pb_40_ alloy (Bi_60_Pb_40_) has a rough lamellar morphology. The Bi_50_Pb_40_Sn_10_ alloy electrocatalyst was fabricated by an expandable and controllable electrochemical etching/dealloying process. This bimetallic electrocatalyst is used for the electroreduction of CO_2_, as shown in Fig. 18g. Its catalytic performance is closely related to its unique structure and composition. The reticular form and rough sheetlike structure provide hollow space and inner cavities, while generating a large surface area and high stability. This leads to an increase in the electrochemical surface area and the number of active sites, thereby enhancing the ability to activate carbon dioxide, transfer electrons and bind adsorbents, and promoting the formation of intermediates and products. In order to better understand the electronic states and adsorption behaviors of the surface composition of Bi–Pb electrodes, DFT simulations were carried out. The reaction pathways for the formation of formate were studied by using the optimal Bi–Pb alloy models such as Bi_100_Pb_0_, Bi_85_Pb_15_ and Bi_60_Pb_40_ with different Pb contents. The Gibbs free energy (ΔG) and competitive HER for the conversion of CO_2_ to formic acid intermediates were evaluated (Fig. 18h, i). It can be seen from Fig. 18h that Bi_85_Pb_15_ and Bi_60_Pb_40_ require ΔG of 0.32 and 0.36 eV respectively for the adsorbed CO_2_ to undergo the first protonation to form the *OCHO intermediate. This value is much smaller than that of Bi_100_Pb_0_ (0.57 eV), and its desorption intensity is relatively weak, which inhibits the formation of formate esters. The results show that a higher Bi content further reduces the binding strength of Pb, which is conducive to the effective desorption of the product. Studies have shown that bicarbonate in aqueous solution is the main proton source for the electrocatalytic conversion of carbon dioxide to HCOOH. The rapid bicarbonate equilibrium between CO_2_ molecules and HCO_3_^−^ is of great significance for maintaining the stability of the *OCHO intermediate. Therefore, the changes in atomic arrangement, along with the adjustments in electronic structure and composition, jointly activate the interaction strength with the electrode surface, thereby maximizing the efficiency of the main product (HCOOH). The prepared nanoscale Bi–Pb electrocatalyst exhibits excellent activity, adjustable selectivity, and long-term operation in the CO_2_RR.

Alloying and polarization engineering enhance the CO_2_RR performance by reconstructing the electronic state density of Bi-based catalysts through atomic orbital hybridization and ferroelectric polarization: bimetallic interfaces (such as Sn–Bi, Sb–Bi) optimize the adsorption of intermediates through the upward shift of the *p* band center, achieving high formic acid selectivity and wide potential adaptability. Polarization effect ferroelectrics promote the separation of bulk phase charges through built-in electric fields. In addition, the combination of alloying and polarization (such as Bi_3_TiNbO_9_/Vo) can form a “static electron regulation–dynamic charge transport” double cycle, enhancing CO_2_ adsorption and intermediate stability. Nanoporous structures (such as np-Sb–Bi) enhance the exposure of active sites through high specific surface area and bimetallic synergy. However, some limitations cannot be ignored. For example, the precise atomic arrangement control required for intermetallic compounds makes the synthesis complex. The Sn–Bi interface may have phase separation due to electrochemical corrosion, resulting in a decrease in stability. Moreover, polarized materials, such as ferroelectrics, are prone to degradation due to the oxygen vacancy binding effect. Thereafter, to improve the catalytic performance and stability, further improvements or multistrategy collaboration are needed for researchers to address these issues.

## Conclusion and Perspectives

### Conclusion

This review comprehensively examines the significant progress in the Bi-based catalysts for CO_2_RR. We highlighted their regulation strategies and the corresponding catalytic mechanisms, aiming to enable researchers to fundamentally understand the structure–property relationship of the improved Bi-based catalysts and then realize their controllable preparation and performance control. The main conclusions are as follows:

- Strategy I—defect engineering: the construction of vacancies, such as Vo, can act as the adsorption and activation center of CO_2_, improving the electron–hole separation efficiency, regulating the energy difference between the antibonding state and the Fermi level, strengthening the chemical bond of the adsorbed material on the surface and promoting the stable chemical reaction process, and thus improve the performance of CO_2_RR.
- Strategy II—atomic doping engineering: can change the band structure of the catalysts, intermediate adsorption behavior, reduce the recombination of electrons and holes effectively, introduce specific active sites, improve the electron density, change the chemical properties of the catalyst surface, and reduce the energy barrier for CO_2_ activation and intermediate conversion.
- Strategy III—organic framework engineering: the combination of a Bi-based catalyst with COF/MOF materials with high porosity and large specific surface area can increase the contact area between the catalyst and reactant and improve the reaction rate. The band structure can be adjusted by designing different organic connection units and metal nodes to optimize light absorption and electron transport performance. COF and MOF materials can be also used as carriers or active layers to improve catalytic efficiency by providing more active sites.
- Strategy IV—inorganic heterojunction engineering: constructing an inorganic heterostructure can enhance quantum efficiency, promote charge transfer, inhibit carrier recombination, increase the turnover frequency of CO_2_ molecules, and thus improve the selectivity of photoelectrocatalytic products.
- Strategy V—crystal face engineering: adopting crystal face engineering can uniquely operate surface atoms on a particularly exposed surface, realize the directional design and control of specific crystal faces, optimize the physicochemical properties of Bi-based catalysts at the atomic level, and then improve the CO_2_RR performance.
- Strategy VI—alloying and polarization engineering: intermetallic alloys that have well-defined atomic arrangements can effectively inhibit CO_2_ poisoning; the unique polarization effect of the Bi-based catalysts can also regulate the electronic structure and the adsorption of CO_2_, and synergistically enhance the CO_2_RR activity.

Also, for the convenience of readers, the six structural regulation strategies, their specific implementation methods, and the corresponding catalytic mechanisms are summarized and listed in Table 1.

**Table 1:** A summary of the six structural regulation strategies, their specific implementation methods, and corresponding catalytic mechanisms

Regulation strategies	Implementation methods	Corresponding catalytic mechanism
Defect engineering	Construct vacancies	Serve as adsorption and activation centers for CO_2_, enhance the efficiency of electron–hole separation, regulate the energy difference between the antibonding state and the Fermi level, strengthen the chemical bonds on the surface, and promote a stable chemical reaction process
Atomic doping engineering	Metal doping/Nonmetal doping/Co-doping	Altering the band/electron structure, intermediate adsorption behavior, reducing the recombination of electrons and holes, introducing specific active sites, increasing the electron density, and changing the chemical properties of the catalyst's surface
Organic framework engineering	Combine with COF/MOF materials	Increase the contact area between the catalyst and the reactants, adjust the band structure, optimize the light absorption and electron transport performance, as a carrier or active layer to provide more active sites
Inorganic heterojunction engineering	Construct Z-/S-shaped inorganic heterojunctions	Enhance quantum efficiency, promote charge transfer, inhibit carrier recombination, and increase the turnover frequency of CO_2_ molecules
Crystal face engineering	Expose specific surfaces for directional design	Achieve directional design and control of specific crystal planes, and optimize the physicochemical properties of Bi-based catalysts at the atomic level
Alloying and polarization engineering	Combine with other metals to form alloys and induce a polarized electric field	Optimize the electronic structure of active sites and inhibit CO_2_ poisoning. Regulate the electronic structure and the adsorption of CO_2_

### Perspectives

**Fig. 19:**
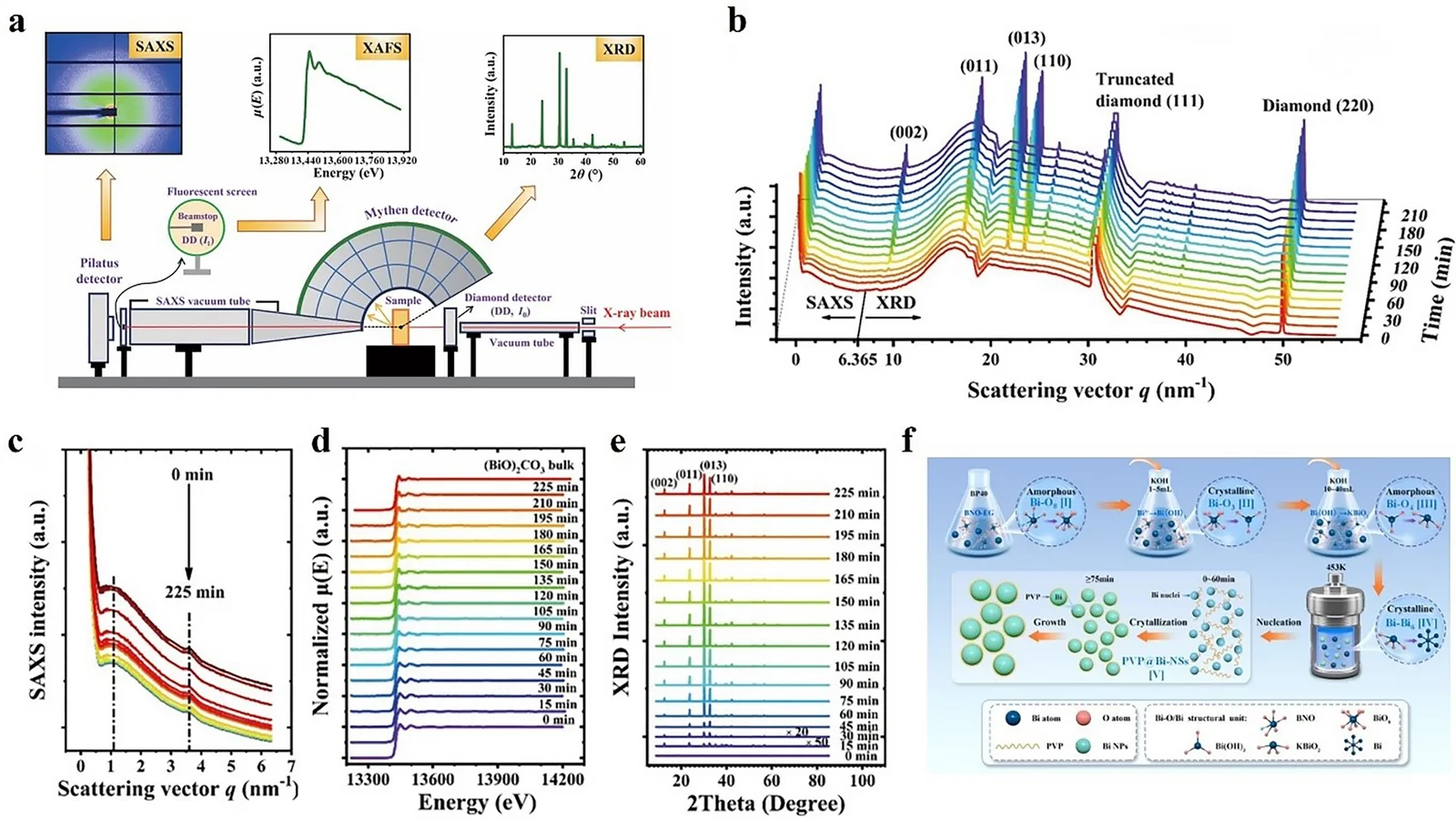
Insights into the dynamic structure evolution and formation mechanism of Bi-based catalysts by SAXS/XRD/XAFS combining technique. **a** Schematic map of the SAXS/XRD/XAFS combined setup [156]; **b-e** in situ SAXS, XRD, and XAFS data collected by the newly developed SAXS/XRD/XAFS combined technique under isothermal isobaric conditions of 423 K and 3 MPa [159]; Copyright © 2024, Science China Press. **f** Schematic illustration of the formation mechanism for the PVP-capped Bi nanospheres (PVP@Bi-NSs) electrocatalyst [160]. © 2024 Elsevier B.V

**Fig. 20:**
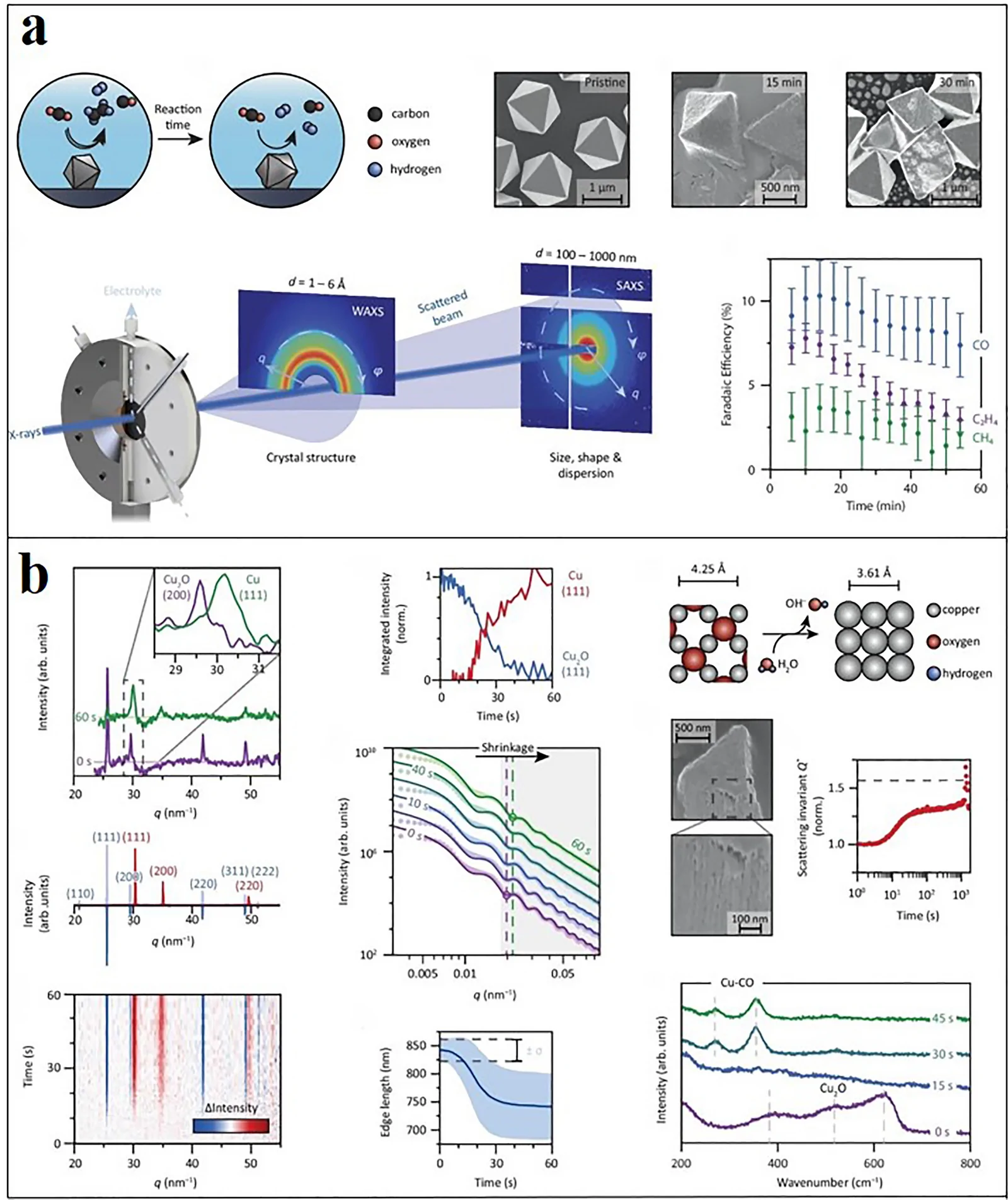
In situ multiscale investigation of activation pathways and uncovering the CO_2_RR mechanism. **a** Electrocatalyst morphology and performance variations after CO_2_ reduction and multiscale in situ X-ray scattering methodology; **b** time-resolved X-ray scattering during the catalyst activation and initial CO_2_RR stage. [161] Copyright © 2025, The Author(s)

Although Bi-based catalysts have unique properties and great potential in CO_2_RR applications, and using the above-mentioned regulation strategies one can also effectively improve their performance, their development still faces many challenges. According to our knowledge and research experiences in Bi-based catalysts, we propose three reasonable prospects or important research directions worthy of further in-depth study:

1. Combination or synergy of multiple regulatory strategies. With the needs of researchers and industrial applications, a single regulation method often cannot meet demand. Two or more regulation strategies have to be combined to try to get the most out of the hierarchical-structured Bi-based catalysts. For example, one can combine defect engineering with organic framework engineering, such as a COF-capped BOC hierarchical-structured Bi-based electrocatalysts can be considered for design. On the one hand, the construction of Vo can accelerate the electron transfer and regulate the electron density of the Bi active site; on the other hand, the COF coating can enhance the adsorption of CO_2_ and improve the hydrophobic properties of the whole catalysts, and the inhibit the HER. The synergistic effect of defect engineering and organic framework engineering will achieve the effect that one plus one is greater than two, which will contribute to the application of catalysts in industrial production.
2. Revealing formation mechanism and realizing controllable synthesis. How to reveal the formation mechanism, and then achieve simple and rapid mass preparation of Bi-based catalysts has been the crucial challenge in this field. In fact, in the processes of synthesis and some dynamic changes, the obtained Bi-based catalysts’ structure is generally hierarchical. Tracking the entire material synthesis process and capturing useful information on all possible metastable precursors and intermediates will indeed facilitate the controllable synthesis of Bi-based catalysts as well as other materials. However, it is difficult for a single technique to meet all these requirements of hierarchical structure characterization. The premise of all this is that advanced in situ characterization techniques are needed to dynamically detect the multiple structural evolutions in the synthesis process. Fortunately, the state-of-the-art combining technique based on synchrotron radiation was proposed and developed and can solve this problem well. Wu's team [156–158] developed a novel small-angle X-ray scattering (SAXS)/X-ray diffraction (XRD)/X-ray absorption fine structure (XAFS) combined technique, as shown in Fig. 19a, which can be used for simultaneous measurements of local atomic structure, nanoscale structure, and microscale structure. Limited by the flux of the first-generation synchrotron radiation (SR) sources, the repeated measurements of the SAXS/XRD/XAFS combined data can usually achieve a time resolution at the second level. In virtue of the multiple advantages of the SAXS/XRD/XAFS combined technique, Liu et al. [159] in situ monitored the isothermal and isobaric synthesis process of CO_2_-assisted BOC photocatalyst using this SAXS/XRD/XAFS combined technique and revealed the evolution process of the initial Bi(OH)_3_ precipitation, early-stage formed KBiO_2_ molecules, intermediate amorphous (BiO)_4_CO_3_(OH)_2_ nanoparticles, and finally crystallized flowerlike BOC particles self-assembled by nanosheets, as shown in Fig. 19b-e. Besides, Liu et al. [160] also unveiled the whole reaction process of PVP-capped Bi nanospheres (PVP@Bi-NSs) electrocatalyst using those combined techniques and found that the formation of PVP@Bi-NSs experienced five stages of Bi-O_8_ (I), Bi-O_3_ (II), Bi-O_4_ (III), Bi–Bi_6_ (IV), and PVP@Bi-NSs (V), as shown in Fig. 19f. The development and application of such a combined technique will very helpful to the rapid mass preparation and controllable synthesis of Bi-based catalysts and other materials. It is worth noting that in the fourth-generation SR sources with high brightness, such as high-energy photon source (HEPS), the time resolution capability of such a combined technique can reach the millisecond or even microsecond level, which will be more conducive to dynamic in situ tracking the rapid synthesis reaction process of catalysts.
3. In situ multiscale investigation of activation pathways and uncovering the catalytic mechanisms. To accurately describe the multistage structural changes in the CO_2_RR process and clarify the catalytic mechanism is an important way to realize the optimal design and performance regulation of the Bi-based catalyst. As we all know, the relationship between catalyst structure and performance (stability, selectivity, and activity) is still heavily studied and is likely affected by dynamic morphological changes under operational conditions. Thus, it is necessary to design an in situ sample cell suitable for photoelectrocatalytic CO_2_ reduction and explore the structural evolution of the catalyst in the catalytic process with the help of in situ multiscale structural characterization techniques in the present and subsequent research. In the latest study, Ruiter et al. [161] presented a multiscale in situ investigation of activation and deactivation pathways of oxide-derived copper electrocatalysts under CO_2_ reduction conditions (Fig. 20a, b). In situ X-ray scattering experiments track morphological changes at small scattering angles and phase transformations at wide angles, with millisecond to second-time resolution and ensemble-scale statistics. These multiscale insights highlight the dynamic and intimate relationship between electrocatalyst structure, surface-adsorbed molecules, and catalytic performance, and the in situ X-ray scattering combined technique serves as an additional tool to elucidate the factors that govern electrocatalyst (de)stabilization. Using advanced technology and data analysis methods to study the catalytic mechanism is very worthy of reference for the research of Bi-based catalysts and other catalytic materials, which can also effectively address the many challenges facing the field of photoelectrocatalysis.
